# Plasma Proteomics Characteristics of Subclinical Vitamin E Deficiency of Dairy Cows During Early Lactation

**DOI:** 10.3389/fvets.2021.723898

**Published:** 2021-12-10

**Authors:** Weidong Qian, Hongyi Yu, Cuiyu Zhang, Hongyou Zhang, Shixin Fu, Cheng Xia

**Affiliations:** Heilongjiang Provincial Key Laboratory of Prevention and Control of Bovine Diseases, College of Animal Science and Veterinary Medicine, Heilongjiang Bayi Agricultural University, Daqing, China

**Keywords:** subclinical VE deficiency, TMT, differentially expressed proteins, biomarkers, plasma

## Abstract

Vitamin E (VE) is an essential fat-soluble nutrient for dairy cows. Vitamin E deficiency leads to immune suppression and oxidative stress and increases the susceptibility of cows to reproductive disorders in the early post-partum period. However, studies on plasma proteomics of VE deficiency have not been reported so far. Therefore, the purpose of this study was to understand the changes of blood protein profile in cows with subclinical VE deficiency in the early post-partum period. In this study, plasma protein levels of 14 healthy cows (>4 μg/ml α-tocopherol) and 13 subclinical VE-deficient cows (2–3 μg/ml α-tocopherol) were analyzed by tandem mass tag (TMT). The results showed that there were 26 differentially expressed proteins (DEPs) in the plasma of cows with subclinical VE deficiency compared with healthy controls. Twenty-one kinds of proteins were downregulated, and five kinds were upregulated, among which eight proteins in protein–protein interactions (PPI) network had direct interaction. These proteins are mainly involved in the MAPK signaling pathway, pantothenic acid and coenzyme A (CoA) biosynthesis, PPAR signaling pathway, and glycosylphosphatidylinositol (GPI)-anchor biosynthesis. The top four DEPs in PPI (APOC3, APOC4, SAA4, PHLD) and one important protein (VNN1) by literature review were further verified by ELISA and Western blot. The expression levels of APOC3, VNN1, and SAA4 were significantly lower than those of healthy controls by ELISA. VNN1 was significantly lower than those of healthy controls by Western blot. VNN1 is closely related to dairy cow subclinical VE deficiency and can be a potential biomarker. It lays a foundation for further research on the lack of pathological mechanism and antioxidative stress of VE.

## Introduction

The development of the dairy industry is closely related to the regulation of vitamin nutrition, which is the key problem in dairy cows' healthy breeding (1). Vitamin E (VE) is one of the most important components of cellular antioxidant systems and involved in maintaining the oxidative stability (2). The main function of VE is to protect lipid peroxidation, scavenge free radicals *in vivo*, so as to maintain the integrity of cell membrane function (3). Dietary vitamin additives, forage, and legume silage are the main sources of VE for dairy cows (4, 5). A large number of studies on dairy cows show that the plasma VE concentration decreased gradually before and after delivery and reached the minimum value before and after calving (6–8).

Although with the continuous progress of dairy cows' feeding and management, serious VE deficiency in dairy cows rarely occurs. However, during the transition period, in order to enhance the antioxidant capacity of cow cells, excessive consumption of vitamin E will be caused to achieve the purpose of scavenging the oxidative free radicals of excess cells (6, 9). This process makes early post-partum cows prone to subclinical VE deficiency and systemic oxidative stress. Plasma concentrations of α-tocopherol in cows at 2–3 μg/ml can be identified as subclinical VE deficiency (10–12). Vitamin E deficiency and oxidative stress are important causes of perinatal dairy cows' susceptibility to productive diseases (13). Studies have found that VE deficiency can increase the risk of diseases such as retention of placenta, hysteritis, and mastitis in cows (13, 14). Because there were no practical early monitoring methods to measure the level of VE, subclinical VE deficiency is difficult to be found in due time, which brings serious economic losses to the dairy industry. Therefore, the search for new characteristic biomarkers of subclinical VE deficiency is a key technical problem to be solved in the early monitoring and rapid diagnosis of subclinical VE deficiency for high-yield dairy cows.

Mass spectrometry (MS) has become the preferred method for large-scale protein identification and characterization due to its sensitivity and specificity (15, 16). The MS analysis has been found to reveal changes in protein expression. These proteins can be identified as intermediate biomarkers of early disease effects (17). Tandem mass tag (TMT) is a kind of chemical label used for molecular recognition and quantification based on MS. The TMT has been established as an effective method for proteome quantification (18). The body fluid often detected in the clinic is blood, which is easy to obtain and contains abundant biological information of physiological and pathological processes (19). In this study, proteomics techniques were used to identify the differentially expressed proteins (DEPs) in the plasma between subclinical VE deficiency and healthy cows in the early post-partum period. To the best of our knowledge, no data have been published on plasma proteins in early lactation with subclinical VE deficiency in dairy cows. Thus, this is a comprehensive study to explore the potential biological significance of DEPs between subclinical VE deficiency and healthy cows, providing valuable insights into subclinical VE deficiency plasma proteins that may be applied for developing diagnostic markers in subclinical VE deficiency.

## Materials and Methods

### Animals and Experimental Design

All animals involved in this study were cared for according to the principles of Heilongjiang Bayi Agricultural University Animal Care and Use Committee (Daqing, China). The experiment was conducted at a large intensive cattle farm in Heilongjiang Province (Suihua, China). All Holstein cows were fed the same total mixed ratio diets (in accordance with NRC 2001 standard) with similar age, parity, body condition score, and milk yield. The cows were fed a total mixed ratio diet during early lactation, which mainly consisted of 39.58% of corn, 19.61% of corn silage, 26.99% of *Leymus chinensis*, 8.48% of soybean meal, and 4.21% of concentrated feed at the early stage of lactation (ingredient, % of DM). The basal diet was formulated to meet the nutrient requirements according to the Feeding Standards of Dairy Cattle in China. Detailed feed composition is shown in Supplementary Table 1.

According to the concentration of α-tocopherol in plasma, the subclinical VE deficiency group (2–3 μg/ml α-tocopherol) and the healthy control group (>4 μg/ml α-tocopherol) were determined (10–12). Finally, after excluding all other cases of perinatal disease, 67 cows were selected as test animals. Thirteen cows were used as the subclinical VE deficiency group (QF), and 14 cows were used as the healthy control group (BQF) for proteomics study. In addition, 40 cows were used for ELISA verification of the screened and identified differential proteins.

### Blood Sample Collection

For this study, blood samples were obtained from the coccygeal veins of 80 transition dairy cows from 0 to 30 days after calving. The blood samples of each cow were collected on an empty stomach in the morning. Plasma was obtained by centrifugation of blood collected in a 10 ml lithium-heparin tube. After centrifugation at 4°C for 10 min (3,000 rpm), the supernatant was collected for secondary centrifugation by high speed (12,000 rpm) for 5 min. The supernatant was placed in a 1.5 ml centrifuge tube and cryopreserved at −80°C until analyzed. All samples used for repetitive analysis were frozen in aliquots, and only vials needed for each assay run were used, to avoid the repetitive thawing and freezing effect.

### Plasma Sample Processing and TMT Labeling

To determine the biomarkers of subclinical VE deficiency, 27 plasma samples were analyzed by protein quality inspection, trypsin digestion, and TMT differential labeling. Total protein concentration determination was assessed using a Bradford method (20) (Enzyme labeled instrument, Thermo: Multiskan MK3, USA). Firstly, the sample was diluted with lysis buffer to make its final concentration fall within the range of standard curve. The diluted sample and standard sample (bovine serum albumin, BSA: Sigma-Aldrich, A2058, AUS; BSA was dissolved into a series of standard protein by lysis buffer) were diluted with 5 and 250 μl protein quantitative dye, respectively, and the light absorption value of standard substance and sample at 595 nm was determined by enzyme label instrument. The standard curve was drawn, and the sample concentration was calculated. Then, the protein concentration of each sample was calculated according to the curve formula, and the protein concentration of each sample could meet the requirements of the next experiment.

After protein quantification, 100 μg of protein per sample solution was put into a centrifuge tube, and the final volume was 100 μl with Dissolution Buffer (Thermo Scientific, PN: 1861436). Then, 25 μl of 100 mM reducing reagent (Thermo Scientific, PN:1861438) was added and incubated at 55°C for 1 h, and 5 μl of 375 mM iodoacetamide solution (Thermo Scientific, PN: 1861445) was added and incubated for 30 min in a dark room. The processed samples were transferred to a 10 kDa ultrafiltration tube (Sartorius, PN: VN01H02), and 200 μl of 100 mM dissolution buffer was added, centrifuged at 12,000 *g* for 20 min, discarded the solution at the bottom of the collecting tube, and repeated four times (pH value should be measured at 8.0). Trypsin (Thermo Scientific, PN: 1862748) was added to the sample (2.5 μl/per sample, 37°C for 14 h). On the next day, the samples were washed with ultrapure water three times, and the bottom of the enrichment tube was lyophilized and then redissolved with 100 mM dissolution buffer.

TandemMassTag™ (TMT™) technology is an *in vitro* peptide labeling technology developed by Thermo Scientific. In this experiment, 27 serum samples were divided into three groups by labeling with 10 isotopes. The amino groups of peptides were specifically labeled and then analyzed by tandem MS. The relative protein content of 10 different samples in each group could be compared simultaneously. After thawing at room temperature, the TMT reagent (10 standard TMT Kit, Thermo Scientific, PN: 90111) was opened, and 0.8 mg of TMT reagent and 41 μl of absolute ethanol were added into each tube and vibrated for 5 min. Then, 100 μl of enzyme digested sample was added (100 μl/sample) and reacted for 1 h at room temperature. Next, 8 μl of 5% of quenching reagent (Thermo Scientific, PN: 1861439) was added and incubated for 15 min to terminate the reaction. After the labeled samples were mixed, vortex oscillation was performed and centrifuged to the bottom of the tube. The sample after vacuum freeze centrifugation is frozen and stored for use (Vacuum freeze dryer, Thermo: SPD2010-230).

### Pre-separation of Enzymatic Peptides and LC–MS/MS Analysis

Rigoll-3000 high performance liquid chromatography system was used to separate the samples at high pH (Beijing Puyuan Jingdian Technology Co., Ltd). The experimental methods in this part refer to the proteomic studies published by Zhao et al. (21). The mixed labeled samples were dissolved in 100 μl mobile phase A (98% ddH_2_O, 2% acetonitrile, pH 10) and centrifuged at 14,000*g* for 20 min, and the supernatant was taken for use. Firstly, the system condition was detected, and 400 μl BSA was used for separation (column temperature 45°C, detection wavelength 214 nm). Then, 100 μl of the prepared sample was separated in mobile phase B (98% acetonitrile, 2% ddH_2_O, pH 10) with a linear gradient of 5–95% over 72 min at the flow rate of 0.7 ml/min, in detail Supplementary Table 2.

Each fraction was injected for nanoLC–MS/MS analysis (high performance liquid chromatography: Thermo Scientific EASY-nLC 1000 System, Nano HPLC; MS system: Thermo, Orbitrap Fusion Lumos). The components obtained by reverse phase separation at high pH were redissolved with 20 μl of 2% methanol and 0.1% formic acid (centrifuged at 12,000 rpm for 10 min), 10 μl of supernatant was aspirated, and the sample was loaded by sandwich method (loading pump flow rate to 350 nl/min for 15 min) and mobile phase B (100% acetonitrile, 0.1% formic acid) with a linear gradient of 6–95% over 75 min at the flow rate of 600 nl/min, in detail Supplementary Table 3.

### Protein Identification and Quantification

The obtained data were processed by UniProt_Bovin (2019.07.16 Download) database for MS. Maxquant, the commercial software supporting Thermo Company, was used to process the original MS file to obtain the quantitative value of the sample. The detailed parameters are retrieved in Supplementary Table 4.

### Bioinformatics Analysis of DEPs

Clusters of Orthologous Groups (COG) analysis is realized by Blast to kyva sequence, and then statistical analysis is carried out to draw the corresponding graph. Gene Ontology (GO) is a standard vocabulary describing the function, location, and activity of genes. It has a tree structure and is the most widely used ontology in molecular biology. KEGG is a Kyoto Encyclopedia of Genes and Genomes (http://www.genome.jp/kegg).

### Enzyme-Linked Immunosorbent Assay

According to the α-tocopherol concentration in plasma, 40 cows were divided into the subclinical VE deficiency group and healthy control group (the same as the Animals and Experimental Design section). Plasma levels of apolipoprotein C3 (APOC3), apolipoprotein A4 (APOC4), serum amyloid protein A4 (SAA4), phosphatidylinositol-glycan-specific phospholipase D (PHLD), and pantetheinase-1 (VNN1) were determined by enzyme-linked immunosorbent assays according to the manufacturer's instructions (ELISA kits, Shanghai Sinovac Biotechnology Co., Ltd., China). Optical density was measured at 450 nm using a microplate reader (Thermo Multiskan FC microplate reader).

### Western Blotting Analysis

Lysates from plasma samples from normal or subclinical VE deficiency were separated on a 10% SDS-PAGE gel, and the proteins were then transferred to a nitrocellulose membrane. The membrane was blocked in TBST containing 5% non-fat milk powder for 1 h and then incubated overnight with primary antibodies against VNN1 protein; the primary antibody used was anti-VNN-1 (dilution 1:1,000, rabbit, LSBio, USA, LS-C374585) and washed three times with TBST (5 min each), and then the membrane was incubated for 1 h at room temperature with horseradish peroxidase conjugated rabbit IgG. Antibody binding was detected using enhanced chemiluminescence ECL Plus Western blotting detection reagents (GE).

### Statistical Analysis

The basic information analyses were performed using SPSS statistical software (V18.0), which was considered significant when the *p*-value was below 0.05. MS data analyses used the Uniprot_BOVIN (2019.07.16 download) database. The original MS of TMT file is processed by Maxquant, a commercial software of Thermo Company. When the *p*-value is 0.05 and fold change 1.2 times, the difference protein is determined to have significant difference (21).

## Results

### Proteomics of TMT Method to Determine Protein Expression

**Figure 1:**
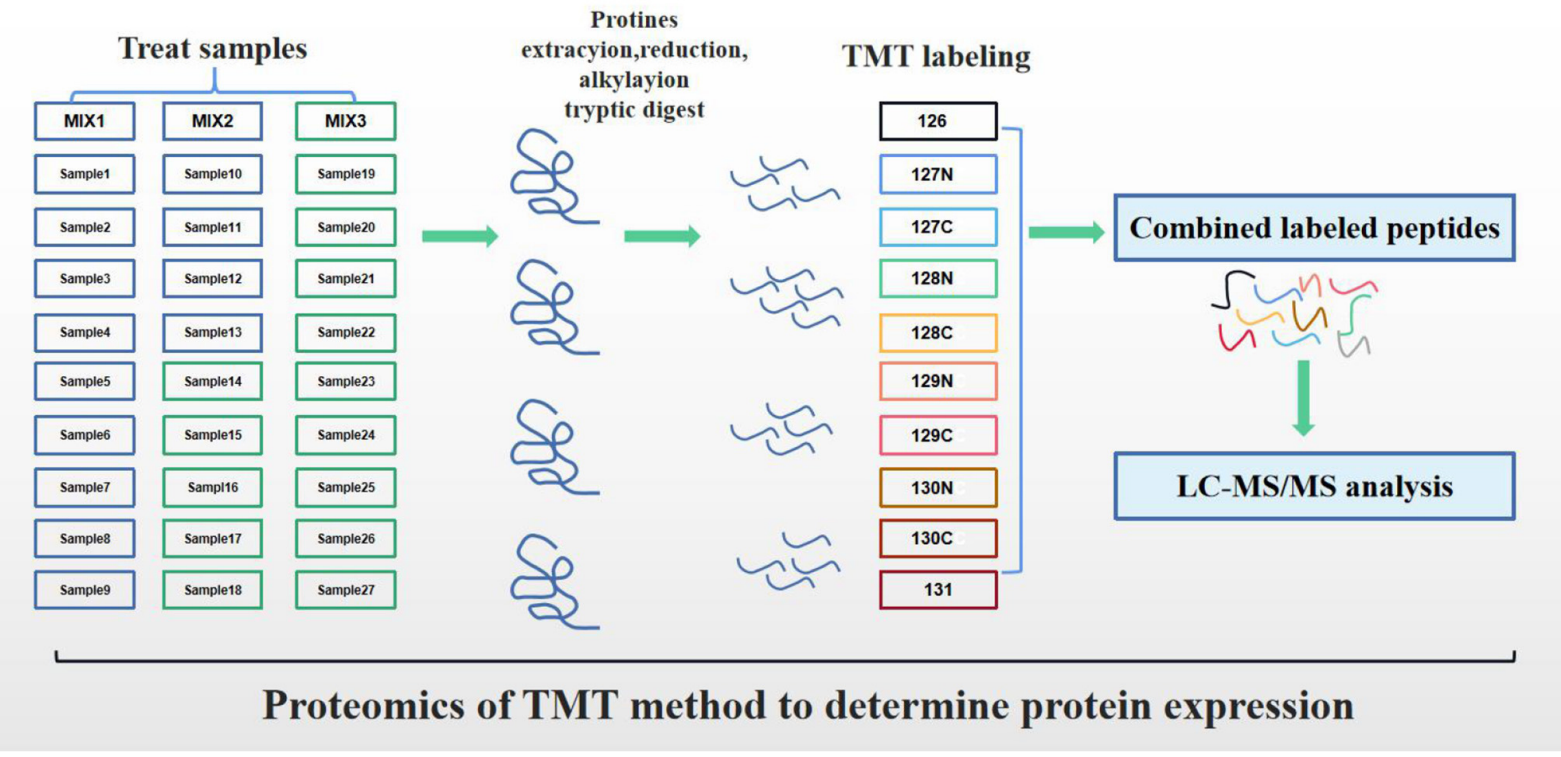
Experimental workflow for proteome analysis.

**Figure 2:**
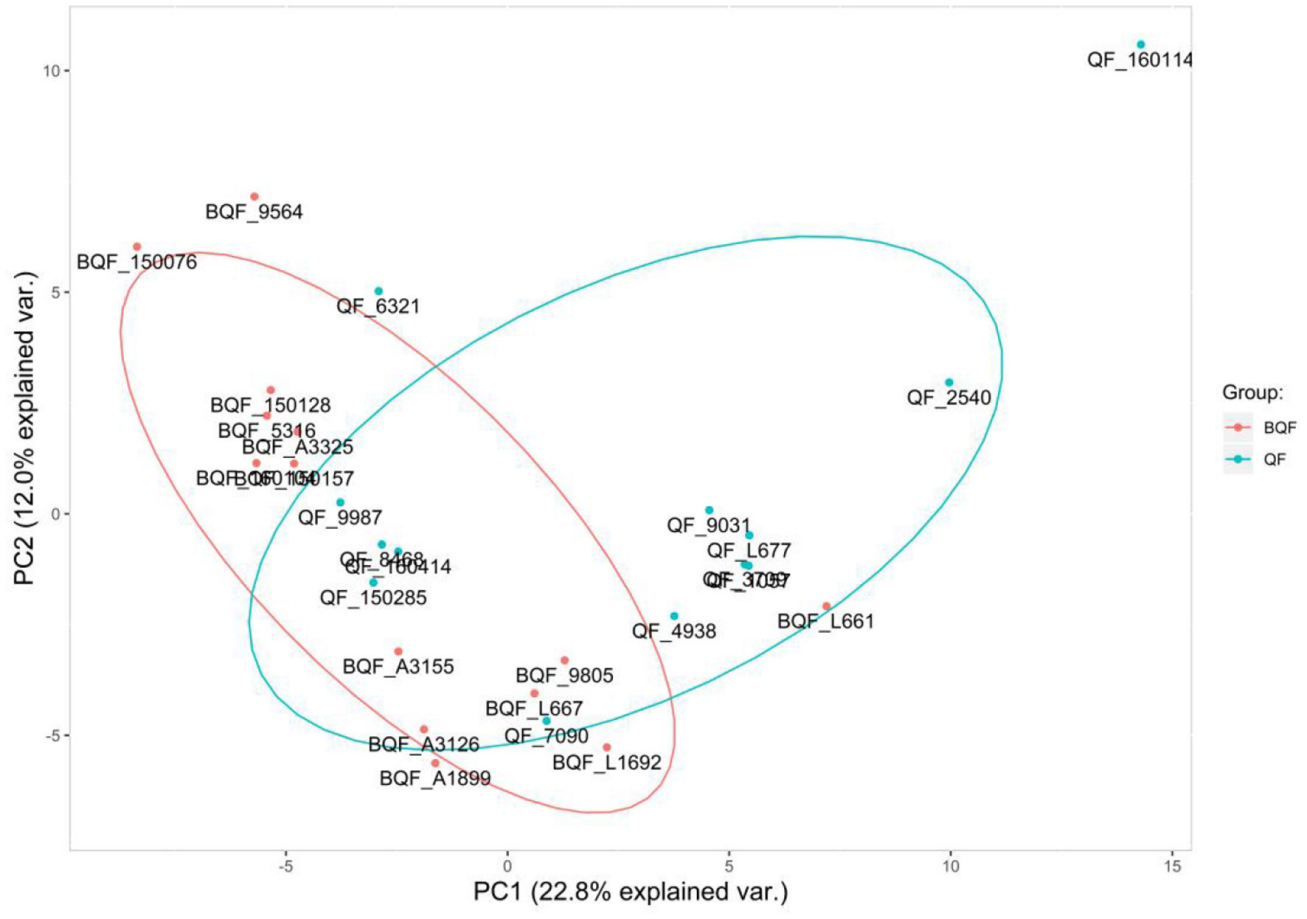
Principal component analysis. The abscissa is the PC1 result, and the ordinate is the PC2 result. Red represents the healthy controls (BQF group), and blue represents the subclinical VE deficiency cows (QF group). It can be seen that there is a certain separation trend between the two groups of samples.

**Figure 3:**
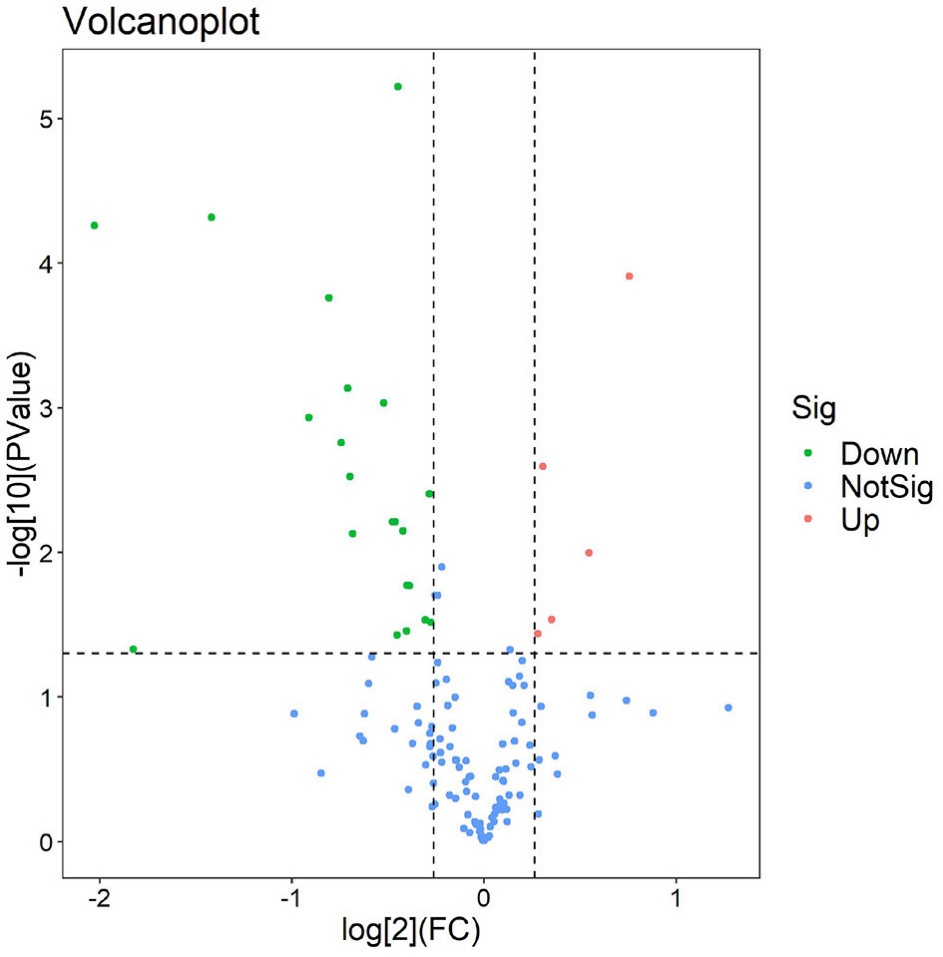
Volcano plot of subclinical vitamin E deficiency vs. healthy control. The volcanic map was drawn using two factors, the fold change (Log2) between the two groups of samples and the *p*-value (–Log10) obtained by the *t*-test, to show the significant difference in the data of the two groups of samples. Red and green dots in the figure are proteins that are significantly differently expressed (1.2 times of fold change and 0.05 of *p*-value). Green dots are downregulated proteins, red dots are upregulated proteins, and blue dots are proteins that have no significant difference.

The experimental workflow of proteomic analysis is shown in Figure 1. The clinical characteristics of the subclinical VE deficiency group (QF) and normal control group (BQF) samples for proteomic analysis are shown in Supplementary Figure 1. According to the Uniprot_BOVIN database, 3,614 peptides (Supplementary Table 5) and 270 proteins (Supplementary Table 6) were identified in the protein qualitative results, which revealed 26 DEPs. The specific information concerning the DEPs is shown in Table 1. Compared with the healthy control group, the DEPs in the plasma of the subclinical VE deficiency group were 21 downregulated proteins and 5 upregulated proteins (Figures 2, 3).

**Table 1:** Proteins showing significant differences in abundance between plasma from cows with subclinical vitamin E deficiency and healthy control.

**ID^a^**	**Gene name**	**Protein name^b^**	**FDR- corrected *p*-value**	**Fold_change^c^**	**Change^d^**
C4T8B4_BOVIN	CRP	Pentraxin	0.000055	−2.030082929	↓
G3X6K8_BOVIN	HP	Haptoglobin	0.046888	−1.826079462	↓
APOC3_BOVIN	APOC3	Apolipoprotein C-III	0.000048	−1.418673822	↓
Q1RMN9_BOVIN	–	C4b-binding protein alpha-like	0.001169	−0.912143442	↓
APOC4_BOVIN	APOC4	Apolipoprotein C-IV	0.000173	−0.808904848	↓
VNN1_BOVIN	VNN1	Pantetheinase	0.001734	−0.743950694	↓
F1MJK3_BOVIN	–	Uncharacterized protein	0.000734	−0.711301256	↓
F1N0H3_BOVIN	CA2	Carbonic anhydrase 2	0.002981	−0.698077617	↓
SAA4_BOVIN	SAA4	Serum amyloid A-4 protein	0.007418	−0.683821812	↓
Q32PA1_BOVIN	CD59	CD59 molecule	0.000926	−0.522529897	↓
Q2KIW1_BOVIN	PON1	Paraoxonase 1	0.00612	−0.476851592	↓
F1MRD0_BOVIN	ACTB	Actin, cytoplasmic 1	0.006124	−0.463760059	↓
LBP_BOVIN	LBP	Lipopolysaccharide-binding protein	0.037565	−0.453306513	↓
PHLD_BOVIN	GPLD1	Phosphatidylinositol-glycan-specific phospholipase D	0.000006	−0.447579644	↓
E1B805_BOVIN	–	Uncharacterized protein	0.007075	−0.422350268	↓
A0A3B0IZF8_BOVIN	C1QC	Adiponectin B	0.035116	−0.403812868	↓
A0A3Q1LU84_BOVIN		Uncharacterized protein	0.01683	−0.400493668	↓
A0A3Q1LL04_BOVIN	YIPF1	Protein YIPF	0.017006	−0.387877785	↓
A0A3Q1LS74_BOVIN	CFH	Complement factor H	0.029566	−0.305647518	↓
F6PSK5_BOVIN	IL1RAP	Interleukin 1 receptor accessory protein	0.003925	−0.285444103	↓
F1MMP5_BOVIN	ITIH1	Inter-alpha-trypsin inhibitor heavy chain H1	0.030626	−0.27646765	↓
FA10_BOVIN	F10	Coagulation factor X	0.036694	0.280035783	↑
HABP2_BOVIN	HABP2	Hyaluronan-binding protein 2	0.002546	0.306909645	↑
COMP_BOVIN	COMP	Cartilage oligomeric matrix protein	0.029325	0.350653389	↑
REG1_BOVIN	–	Regakine-1	0.01005	0.546086942	↑
CD14_BOVIN	CD14	Monocyte differentiation antigen CD14	0.000123	0.756306843	↑

a *ID from NCBI protein database for BOVIN*.

b *Displays the protein name of the comment in the Fasta header column*.

c *Fold changes calculated as: –log2 (mean disease/mean control), mean value of peak area obtained from the QF group/mean value of peak area obtained from the BQF group. Choose p-value 0.05 and fold change 1.2 times as significant difference (21)*.

d *“↑”, upregulated plasma proteins; “↓”, downregulated plasma proteins*.

### Functional Annotation and Analysis

**Figure 4:**
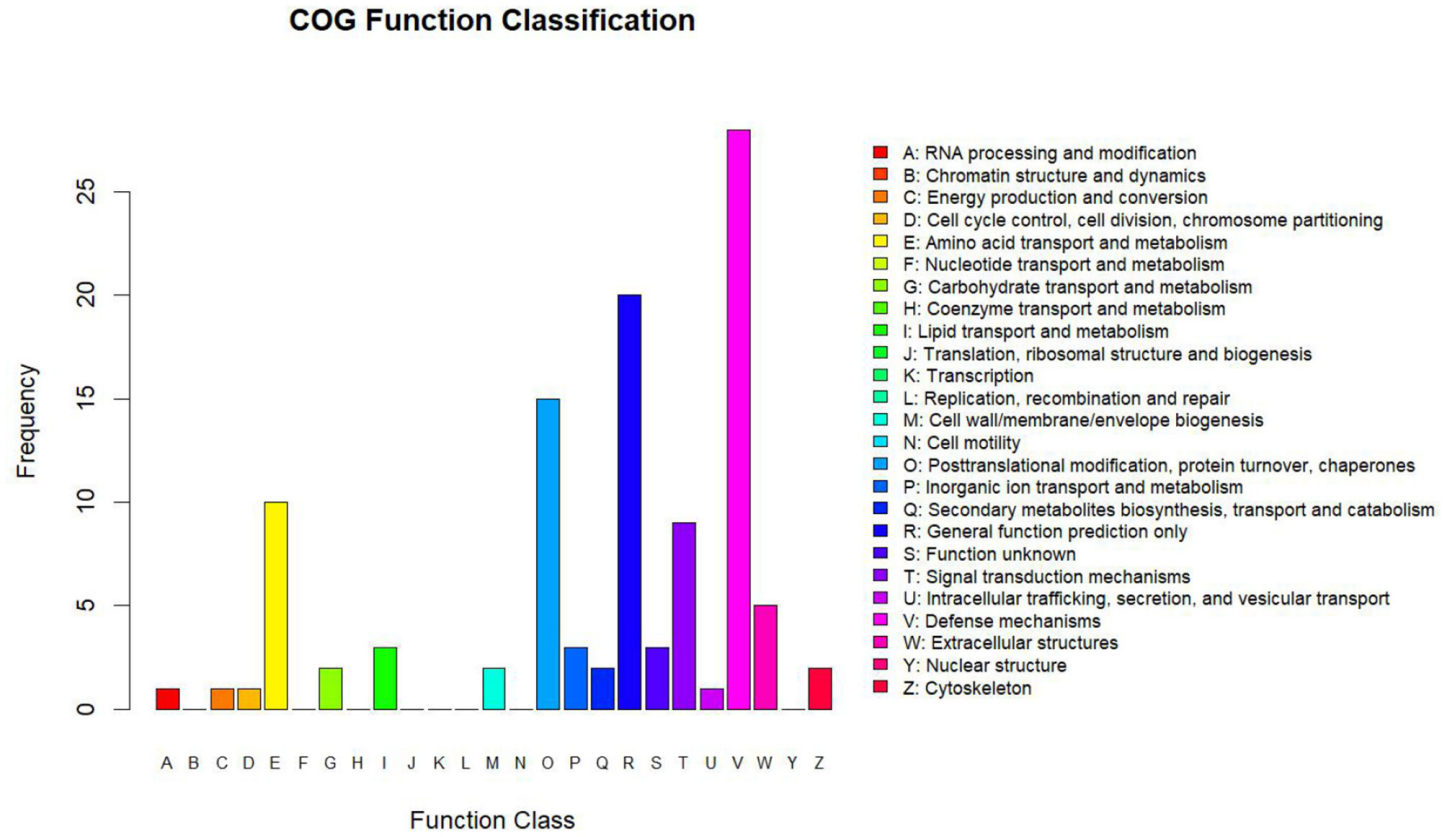
Statistics of COG function classification. The abscissa shows the COG function classification, and the ordinate shows the number of proteins by functional classification.

Clusters of Orthologous Groups analysis was based on the homologous classification of gene products based on the COG database. The analysis identified protein ortholog classifications *via* the COG database, allowing us to predict the possible functions of these proteins and potentially uncover further functional classifications. The highest protein functional classifications were defense mechanisms with 28 proteins, general function prediction with 20 proteins, post-translational modification, protein turnover, and chaperones with 15 proteins, amino acid transport and metabolism with 10 proteins, and signal transduction mechanisms with 15 proteins (Figure 4).

**Figure 5:**
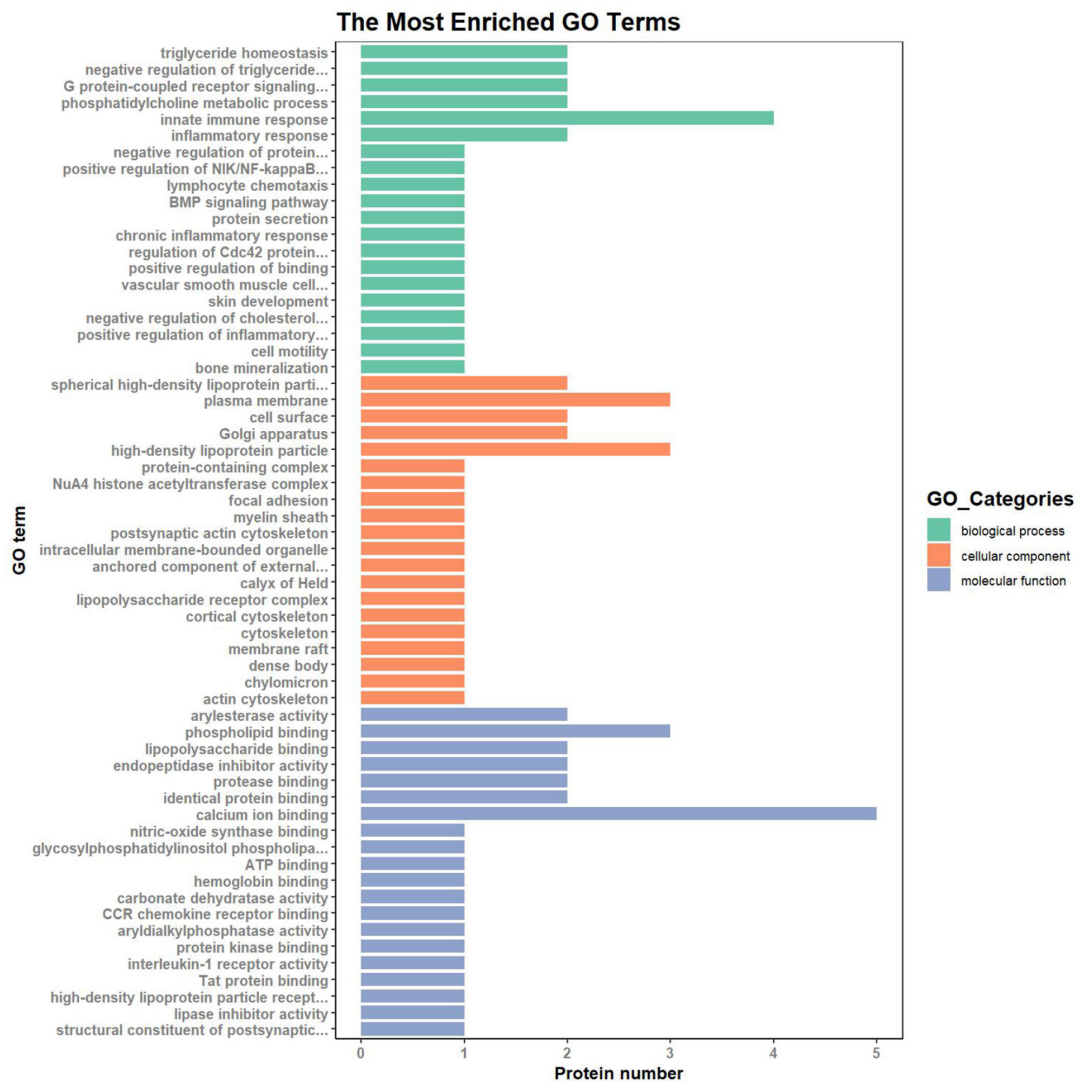
GO (Gene Ontology) analysis of subclinical VE deficiency-related proteins. Green represents biological process, orange represents cellular process, and blue represents molecular process. Classification of 26 DEPs based on biological process, molecular function, and subcellular localization. The abscissa represents the number of differential proteins in each functional category. Based on BP, these subclinical VE deficiency-related proteins were mainly involved in innate immune response and triglyceride homeostasis. The higher enrichment of CC was the plasma membrane and high-density lipoprotein particle, and the functions of higher MF enrichment were calcium ion binding and phospholipid binding.

To understand the cellular and molecular functions (MF) of subclinical VE deficiency-related proteins, GO analysis of these related proteins was performed based on biological processes (BP), cellular components (CC), and MF. In this analysis, the protein number was used to assess the importance of subclinical VE deficiency-related proteins in regulating cellular and molecular functions (Figure 5). Based on BP, these subclinical VE deficiency-related proteins were mainly involved in innate immune response, triglyceride homeostasis, negative regulation of triglyceride, G protein-coupled receptor signaling, inflammatory response, and phosphatidylcholine metabolic process. The higher enrichment of CC was the plasma membrane and high-density lipoprotein (HDL) particle, and the functions of higher MF enrichment were calcium ion binding and phospholipid binding.

**Figure 6:**
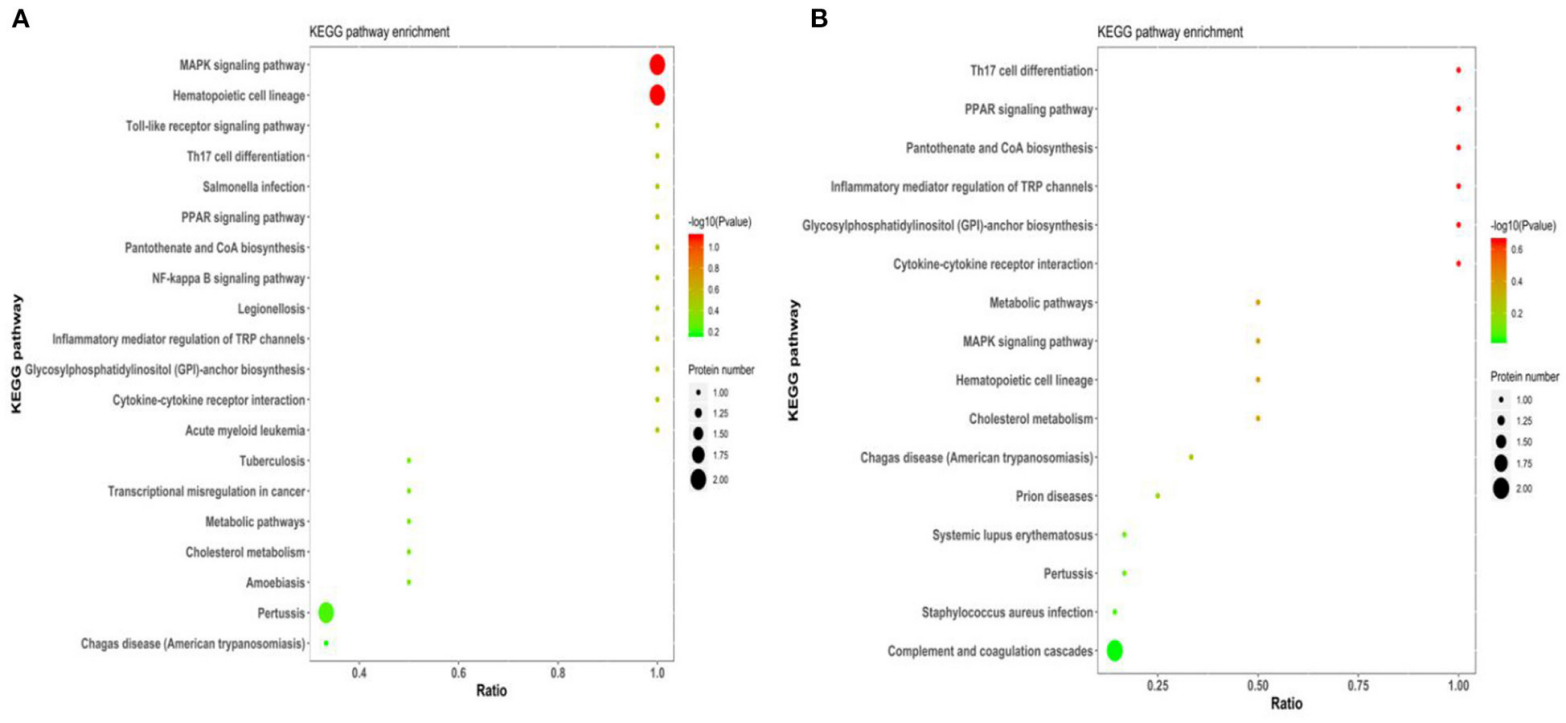
KEGG pathway of subclinical VE deficiency-related proteins enrichment results. Abscissa is ratio, ordinate is each KEGG pathway entry, color represents enrichment [–log10 (*p*-value)], and circle size represents protein number. **(A)** The enrichment results of all differential proteins. **(B)** The enrichment results of downregulated differential proteins.

For KEGG signaling pathway analysis, these subclinical VE deficiency-related proteins are mainly involved in the MAPK signaling pathway, and these subclinical VE deficiency downregulated differential-related proteins are mainly involved in pantothenate and CoA biosynthesis, PPAR signaling pathway, and glycosylphosphatidylinositol (GPI)-anchor biosynthesis (Figure 6).

### Interaction Network Between Proteins

**Figure 7:**
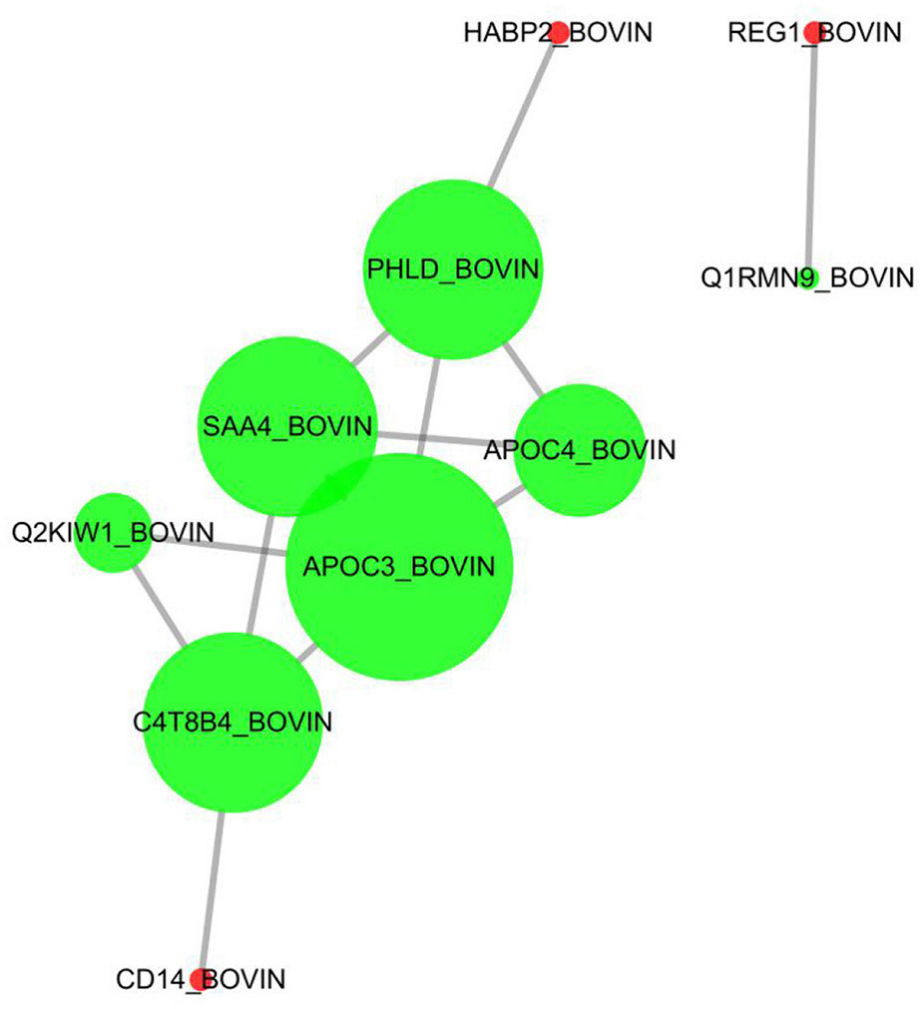
Protein–protein directive interaction network. Ten among 26 DEPs were predicted to have directive protein–protein interactions. The interactions were based on “evidence” mode and of medium confidence. Nodes represent proteins, and edges represent protein–protein interactions. Degree determines the node size, and combined_score determines the edge size.

In this study, 26 differential proteins were obtained through high-throughput screening. Based on the STRING PPI (protein–protein interaction) database and Cytoscape tools, we established a PPI network and found that 10 of these proteins have direct interactions. In the network, the number of proteins that directly interact with a certain protein A is called the connection degree of protein A. Generally speaking, the greater the connection degree of a protein, the greater the disturbance to the entire system when the protein changes; this protein may be the key to maintaining the balance and stability of the system. The Cytoscape was used as a tool to set the size of the node to reflect the degree of the node. A larger node indicates a higher degree of the node. The top four proteins were selected in the degree of the node in this network as candidate proteins for subsequent experimental verification, namely, APOC3, APOC4, SAA4, and PHLD (Figure 7).

### Reduced Plasma Levels of VNN1, SAA4, APOC3, APOC4, and PHLD in Cows With Subclinical VE Deficiency

**Figure 8:**
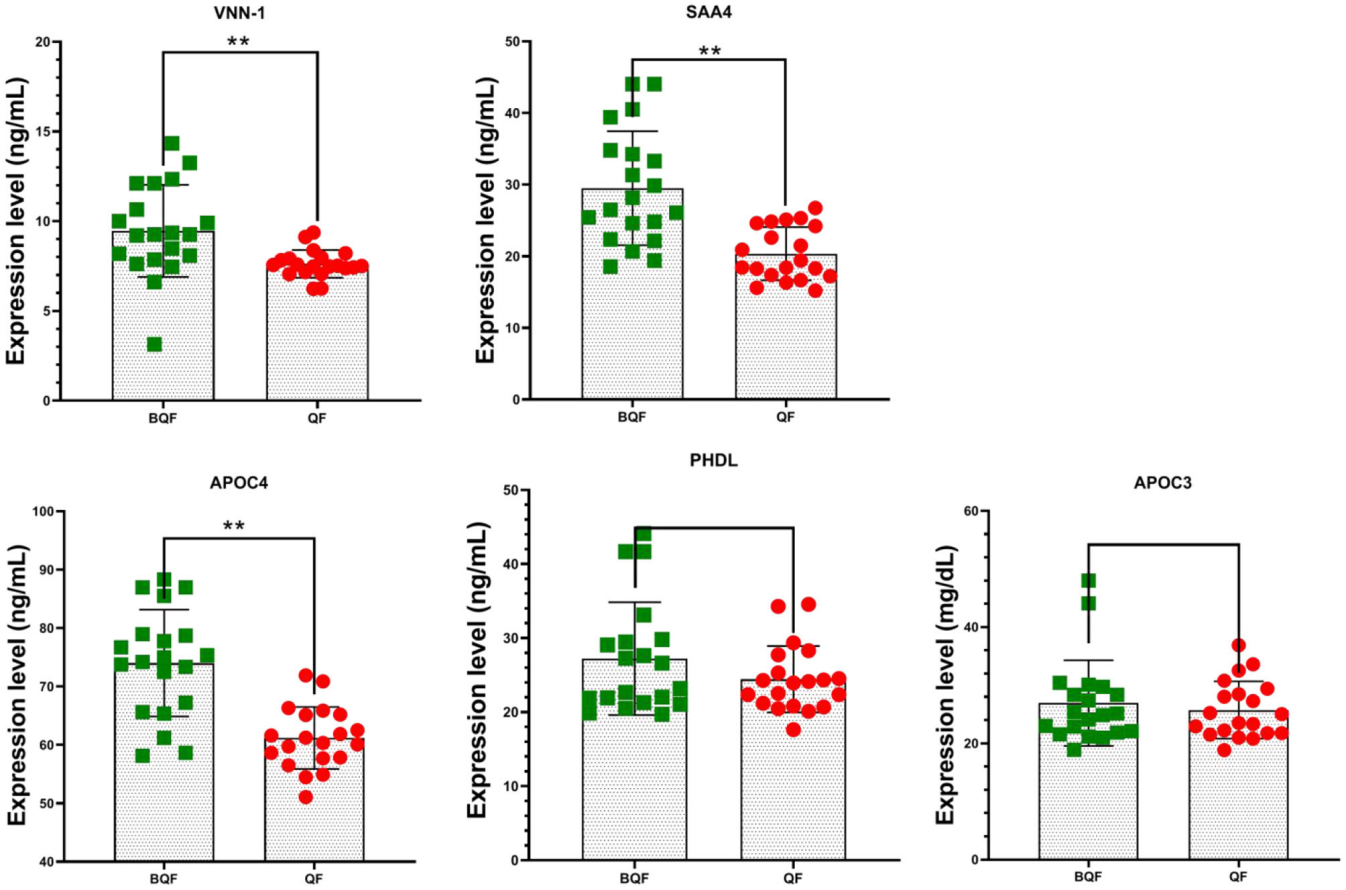
Five plasma protein levels in subclinical VE deficiency cows (QF) and healthy controls (BQF) were selected for verification using enzyme-linked immunosorbent assay in subclinical VE deficiency cows (*n* = 20) and healthy controls (*n* = 20). Plasma levels of VNN-1, SAA4, and APOC4 were significantly lower in subclinical VE deficiency cows than those of the control group (*p < 0.05, **p < 0.01), while no significant differences were detected in the expression of APOC3 and PHDL (*p* > 0.05).

To validate differentially expressed candidate proteins between the subclinical VE deficiency group and the healthy control group, 40 plasma samples were verified (20 subclinical VE deficiency and 20 healthy plasma) by ELISA. The top four DEPs in PPI (APOC3, APOC4, SAA4, PHLD) and one important protein (VNN1) by literature review were further verified by ELISA. The results showed that VNN1, SAA4, and APOC4 were significantly downregulated in subclinical VE deficiency plasma samples (*p* < 0.05; Figure 8), while the expression levels of PHLD and APOC3, protein were not significantly changed (*p*>0.05).

### Confirmation of Differentially Regulated Proteins by Western Blot

**Figure 9:**
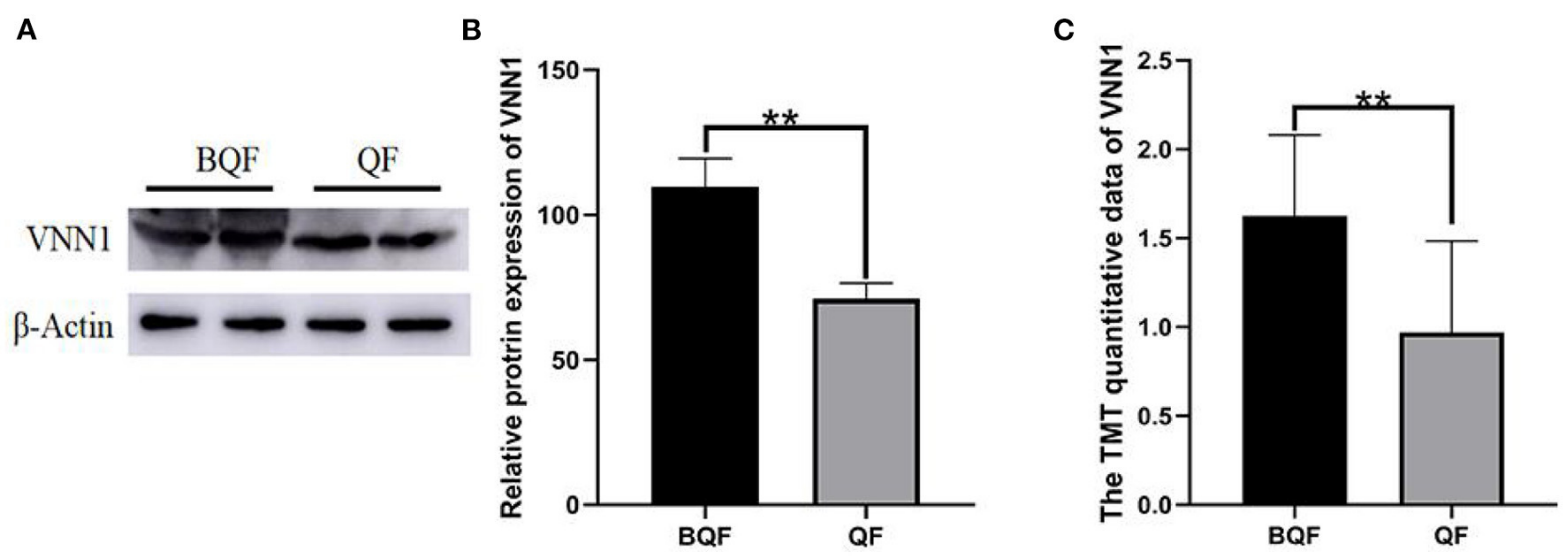
Validation of VNN-1 by immunoblot analysis. **(A)** Representative immunoblotting of DEPs between subclinical VE deficiency cows (QF) and controls (BQF). **(B)** Quantification of relative gray values of VNN-1 compared with actin (**p < 0.01). **(C)** The TMT quantitative data of VNN1 between QF group and BQF (**p < 0.01).

Four samples (the subclinical VE deficiency group and the healthy control group) were selected from the collected plasma samples, and Western blot analysis was performed on one important protein, VNN-1 to verify the results of LC–MS/MS. Figure 9 shows a Western blot image that can quantify proteins. The results of Western blot provide reliable evidence for TMT proteomics.

## Discussion

The aim of this study was to report a comprehensive analysis of DEPs in the plasma of early subclinical VE deficiency and healthy cows using TMT-labeled quantitative proteomics. Twenty-six plasma proteins were changed in the subclinical VE deficiency group, of which 21 proteins were downregulated, and 5 proteins were upregulated. This is a comprehensive study to explore the potential biological significance of DEPs between subclinical VE deficiency and healthy cows, providing valuable insights into subclinical VE deficiency plasma proteins that may be applied for developing diagnostic markers in subclinical VE deficiency.

Those DEPs are mainly involved in innate immune response, triglyceride homeostasis, and negative triglyceride regulation according to GO analysis. The changes of regakine-1, coagulation factor X (F10), CD59, haptoglobin (HP), lipopolysaccharide-binding protein (LBP), and serum amyloid A-4 protein (SAA4) involved in congenital immune response are noteworthy in the DEPs. Synergistic effects of regakine-1 with other neutrophil chemokines suggest that it also enhances inflammatory responses to infection (22). F10 regulates inflammatory signaling by inducing the expression of interleukin (IL)-6, IL-8, monocyte chemotactic protein-1, and intracellular adhesion molecules (23, 24). CD59 is a key regulator of the complement system, which inhibits the formation of the MAC terminal pathway by binding to C9 or C5B-8 to prevent complement attack (25). Haptoglobin is an acute phase protein released by hepatocytes in response to the production of pro-inflammatory cytokines in an inflammatory state (26). Lipopolysaccharide-binding protein is an acute phase protein synthesized in the liver that is involved in the host response to both Gram-negative and Gram-positive pathogens (27); it also promotes the presentation of lipopolysaccharide LPS to CD14 and induces the release of pro-inflammatory cytokines (28). Serum amyloid A-4 protein is a secondary apolipoprotein on HDL in plasma (29). Studies have shown that the concentration of SAA4 decreases when the body is under inflammation (30, 31). The expression of regakine-1 and F10 was upregulated. It indicates that subclinical VE deficiency may aggravate the inflammatory response of the body and increase the risk of infection with other diseases. The expression of CD59, CRP, HP, LBP, and SAA4 was downregulated in the subclinical deficiency group, suggesting that the subclinical deficiency of VE may impair the innate immune response of the body, leading to inflammation and immunosuppression. However, its molecular mechanism needs to be further studied.

The involvement of hyaluronan-binding protein 2 (HABP2), apolipoprotein C4 (APOC4), paraoxonase 1 (PON1), complement factor H (CFH), inter-alpha-trypsin inhibitor heavy chain H1 (ITIH1), and APOC3 in triglyceride homeostasis and negative triglyceride regulation is significant in DEPs. Hyaluronan-binding protein 2 is a calcium-dependent serine protease that provides structural and functional integrity for cells and plays an important role in blood coagulation and fibrinolysis (32). Apolipoprotein C-IV expression is regulated by the oxisome proliferation-activated receptor complex and is associated with hepatic steatosis (33). Paraoxonase 1 is a mammalian antioxidant/anti-inflammatory enzyme synthesized in the liver and secreted into the blood (34). Complement factor H is a major MDA binding protein that can induce the pro-inflammatory effects of MDA (35). Inter-alpha-trypsin inhibitor heavy chain H1 is one of the heavy chains of serine protease inhibitors that carry hyaluronic acid in plasma and play a role in inflammation and carcinogenesis (36). APOC3 is recognized as one of the most important regulators of plasma triglyceride (37). APOC3 of bovine is a low molecular weight protein mainly synthesized by the liver and mainly distributed in HDL (38, 39). Under normal conditions, APOC3 concentration in bovine plasma was the lowest in the non-lactation period and gradually increased in the early lactation stage. In the early lactation period, the plasma APOC3 concentration of cows with fatty liver and ketosis was lower than that of healthy cows (40, 41). In the subclinical VE deficiency group, HABP2 was upregulated, while CFH, ITIH1, and APOC3 were downregulated, indicating that subclinical VE deficiency can aggravate lipid peroxidation, cause oxidative stress induced by inflammatory response, and may cause hyaluronic acid synthesis and transport disorders at the cellular level, thus affecting extracellular matrix synthesis and changing cell structural integrity (42). Paraoxonase 1 is involved in the hydrolysis of lipid hydroperoxides and phospholipids produced during oxidative stress (43), which may be the main reason for the downregulation of VE in subclinical deficiency.

The main typical pathways were identified between subclinical VE deficient and control cows by KEGG. For KEGG signaling pathway analysis, these subclinical VE deficiency-related proteins are mainly involved in the MAPK signaling pathway, and these subclinical VE deficiency downregulated differential-related proteins are mainly involved in pantothenate and CoA biosynthesis, PPAR signaling pathway, GPI-anchor biosynthesis. Mitogen-activated protein kinases (MAPKs) are a kind of protein kinases that regulate cell proliferation, differentiation, apoptosis, and migration (44). Mitogen-activated protein kinase activation interacts with protein lipase to change cell behavior rapidly in response to environmental changes (45). The upregulated protein monocyte differentiation antigen CD14 (CD14) and downregulated protein interleukin 1 receptor accessory protein (IL-1RAP) were related to the MAPK signaling pathway. CD14 is a pattern recognition receptor (PRR) of the innate immune system. After recognizing pathogen associated molecular pattern (PAMP), CD14 transmits signals to cells to activate transcription factors and initiate inflammatory reaction (46). Interleukin 1 receptor accessory protein is a kind of auxiliary protein IL-1 signaling pathways, involved in the IL-1 functional receptors (IL-1R I) that both belong to the immunoglobulin superfamily member (47). Interleukin 1 receptor accessory protein increases the supply of cysteine through uptake and biogeneration and controls cysteine metabolism to participate in regulating oxidative stress (48). The downregulation of plasma IL1RAP in dairy cows with subclinical VE deficiency may be due to its involvement in the regulation of cysteine metabolism. Cysteine metabolism is a key substrate and determinant of antioxidant glutathione (GSH) synthesis (48). Plasma CD14 protein expression was up-regulated and IL-1RAP protein expression was down-regulated, suggesting that stress and inflammation were more serious in cows with subclinical VE deficiency.

Glycosylphosphatidylinositol-anchored modification is one of the most common post-translational modifications of eukaryotic cell membrane proteins (49, 50). Glycosylphosphatidylinositol-anchored biosynthesis pathway related protein phosphatidylinositol glycosyl specific phospholipase D (GPLD) was downregulated in post-partum VE-deficient dairy cows. Glycosylated phosphatidylinositol specific phospholipase D (GPI-PLD) in plasma can specifically act on the GPI-anchored substrate in the presence of detergents, thus releasing anchored proteins and phospholipid acids (51). Liver is an important source of plasma GPI-PLD in both human and bovine (52, 53). The lysosomes of hepatocytes are rich in GPI-PLD, which plays an important role in the degradation of GPI and GPI-anchored proteins in hepatocytes. It is speculated that GPI-PLD in hepatocytes may enter plasma with HDL secreted by hepatocytes (54). Therefore, liver diseases may affect the activity of GPI-PLD in plasma. Downregulation of plasma GPLD1 in dairy cows with subclinical VE deficiency indicated that oxidative stress aggravates abnormal liver metabolism and abnormal degradation of GPI and GPI-anchored protein of dairy cows with subclinical VE deficiency.

Pantothenic acid (PA) and its salts, as a component of coenzyme A (CoA) or acyl carrier protein (ACP), play an important role in many metabolic reactions (55). Coenzyme A-bound PA is involved in the energy release of carbohydrates, fatty acids, and amino acids. The PA binding to ACP is related to the synthesis of fatty acids (56). In the post-partum subclinical VE deficiency group, PA and CoA biosynthesis pathway-related protein pantothenyl thioglycolaminase (VNN1) were downregulated. VNN1 is a kind of oxidative stress sensor rich in the liver, which is a GPI-anchored pantothenase. It is involved in the regulation of multiple metabolic pathways and is highly expressed in the liver, intestine, and kidney (57, 58). Its pantothenase activity hydrolyzes PA into PA (vitamin B5) and cysteamine (59). Some studies have shown that VNN1 deficiency can increase liver GSH levels (60, 61). In this study, VNN1 was downregulated in subclinical VE-deficient dairy cows after middle production, which may be due to the increase of liver GSH level to resist oxidative stress.

Subclinical VE deficiency in cows in the early post-partum period can aggravate oxidative stress and inflammation and aggravate abnormal lipid metabolism in cows (11, 62, 63). Vitamin E is a major protective agent for circulation and intracellular lipid peroxidation, which can reduce the level of cellular oxidative stress and improve the functional environment of intracellular signaling pathways. It has anti-inflammatory and antioxidant effects (64). The five candidate proteins (APOC3, APOC4, SAA4, PHLD, and VNN1) were identified by interaction network analysis and literature review. The DEPs were further verified by enzyme-linked immunosorbent assay and Western blot. APOC3, VNN1, and SAA4 all had significantly lower expression levels than the healthy control group. APOC3 plays a role in PPARα metabolism by controlling lipolysis of PPARα ligands (65). VNN1 is an important target gene of PPARα, which participates in regulating its activity (66–68). VNN1 is involved in oxidative stress and inflammation by regulating the synthesis of cysteamine and GSH (69, 70). Under physiological conditions, SAA4 accounts for more than 90% of the total SAA (29). However, its concentration did not increase in the inflammatory state, but showed a downward trend (30, 31). As a new molecule of concern, SAA4 may be one of the diagnostic markers of post-partum cow subclinical VE deficiency. More research is needed to explore the regulatory mechanisms of APOC3, VNN1, and SAA4 proteins against the dairy cow subclinical VE deficiency and how the three proteins interact.

## Conclusions

In this study, proteomic TMT methods were used to reveal the subclinical deficiency of cow VE in the early post-partum period and changes in plasma protein abundance in healthy control. Subclinical VE-deficient cows aggravate oxidative stress, abnormal lipid metabolism, and immunosuppression. The top canonical pathways and biological functions identified by KEGG and GO indicate this. Based on the different abundance of proteins in these pathways, fat mobilization, ROS production, and inflammatory immune response of subclinical VE-deficient dairy cows increased, which would cause the body to be susceptible to infection. These changes of oxidative stress and inflammation-related proteins may be related to early lactation diseases and slow recovery of reproductive performance. These findings contribute to further research to better understand the molecular mechanism of protein changes that may promote inflammation and oxidative stress.

## Data Availability Statement

The data presented in the study are deposited in the IPROX repository, ProteomeXchange ID: PXD026856. The link is https://www.iprox.cn/page/PSV023.html;?url=1624347711767BLKj view password is jWOm.

## Ethics Statement

The animal study was reviewed and approvexsd by Heilongjiang Bayi Agricultural University Animal Care and Use Committee.

## Author Contributions

WQ, SF, and CX conceived the study, interpreted the data, and wrote the manuscript. WQ, HY, CZ, XS, HZ, SF, and CX carried out experiments and data analysis. All authors approved the final version.

## Funding

This work was supported by the Natural Science Foundation of Heilongjiang Province (ZD2021C006), National Natural Science Foundation of China (Beijing, China; Grant Number 31873028), and Daqing Guiding Science and Technology Plan Project (zd-2020-49). Heilongjiang Bayi Agricultural University's scientific research start plan for introducing talents(XYB202101); Heilongjiang Natural Science Foundation joint guide project (LH2020C085) and supported by the special Fund for the construction of modern agricultural (dairy cow) industrial technology system (CARS-36).

## Conflict of Interest

The authors declare that the research was conducted in the absence of any commercial or financial relationships that could be construed as a potential conflict of interest.

## Publisher's Note

All claims expressed in this article are solely those of the authors and do not necessarily represent those of their affiliated organizations, or those of the publisher, the editors and the reviewers. Any product that may be evaluated in this article, or claim that may be made by its manufacturer, is not guaranteed or endorsed by the publisher.

## References

[1] National Research Council. Nutrient Requirements of Dairy Cattle. 7th Rev Ed. (2001). Washington, DC: The National Academies Press. 10.17226/9825

[2] Vagni S Saccone F Pinotti L Baldi A. Vitamin E bioavailability: past and present insights. Food Nutr Sci. (2011) 2, 16361. 10.4236/fns.2011.210146

[3] Halliwell B. Free radicals and antioxidants - quo vadis? Trends Pharmacol Sci. (2011) 32:125–30. 10.1016/j.tips.2010.12.002 21216018

[4] Herdt TH Stowe HD. Fat-soluble vitamin nutrition for dairy cattle. Vet Clin North Am Food Anim Pract. (1991) 7:391–415. 10.1016/S0749-0720(15)30796-9 1893278

[5] Beeckman A Vicca J Van Ranst G Janssens GPJ Fievez V. Monitoring of vitamin E status of dry, early and mid-late lactating organic dairy cows fed conserved roughages during the indoor period and factors influencing forage vitamin E levels. J Anim Physiol Anim Nutr (Berl). (2010) 94:736–46. 10.1111/j.1439-0396.2009.00956.x 20050949

[6] Pinotti L Manoni M Fumagalli F Rovere N Tretola M Baldi A. The role of micronutrients in high-yielding dairy ruminants: choline and vitamin E. Ankara Univ Vet Fak Derg. (2020) 67:209–14. 10.33988/auvfd.695432

[7] Politis I. Reevaluation of vitamin E supplementation of dairy cows: bioavailability, animal health and milk quality. Animal. (2012) 6:1427–34. 10.1017/S1751731112000225 23031515

[8] Weiss WP. Requirements of fat-soluble vitamins for dairy cows: a review. J Dairy Sci. (1998) 81:2493–501. 10.3168/jds.S0022-0302(98)70141-9 9785241

[9] Sun X Chang R Tang Y Luo S Jiang C Jia H. Transcription factor EB (TFEB)-mediated autophagy protects bovine mammary epithelial cells against H_2_O_2_-induced oxidative damage *in vitro*. J Anim Sci Biotechnol. (2021) 12:35. 10.1186/s40104-021-00561-7 33685494 PMC7941962

[10] Bayfield RF Mylrea PJ. Carotenoid and tocopherol levels in the serum of apparently healthy dairy cattle. J Dairy Res. (1969) 36:137–44. 10.1017/S002202990001262030886898

[11] Frye TM Williams SN Graham TW. Vitamin deficiencies in cattle. Vet Clin North Am Food Anim Pract. (1991) 7:217. 10.1016/S0749-0720(15)30817-3 2049667

[12] Weiss WP Hogan JS Smith KL Hoblet KH. Relationships among selenium, vitamin E, and mammary gland health in commercial dairy herds. J Dairy Sci. (1990) 73:381–90. 10.3168/jds.S0022-0302(90)78684-5 2329203

[13] Naderi M Keyvanshokooh S Salati AP Ghaedi A. Proteomic analysis of liver tissue from rainbow trout (*Oncorhynchus mykiss*) under high rearing density after administration of dietary vitamin E and selenium nanoparticles. Comp Biochem Physiol D Genomics Proteomics. (2017) 22:10–9. 10.1016/j.cbd.2017.02.001 28187310

[14] Weiss WP. A 100-Year Review: From ascorbic acid to zinc-mineral and vitamin nutrition of dairy cows. J Dairy Sci. (2017) 100:10045–60. 10.3168/jds.2017-12935 29153154

[15] Aebersold R Mann M. Mass spectrometry-based proteomics. Nature. (2003) 422:198–207. 10.1038/nature01511 12634793

[16] Cravatt BF Simon GM Yates JR III. The biological impact of mass-spectrometry-based proteomics. Nature. (2007) 450:991–1000. 10.1038/nature06525 18075578

[17] Xu Q Li X Ma L Loor JJ Coleman DN Jia H. Adipose tissue proteomic analysis in ketotic or healthy Holstein cows in early lactation1. J Anim Sci. (2019) 97:2837–49. 10.1093/jas/skz132 31267132 PMC6606492

[18] Thompson AJ Schäfer Kuhn K Kienle S Schwarz J Schmidt G. Tandem mass tags: a novel quantification strategy for comparative analysis of complex protein mixtures by MS/MS. Anal Chem. (2003) 75:1895–904. 10.1021/ac060310l 12713048

[19] Wang T Shen H Deng H Pan H He Q Ni H. Quantitative proteomic analysis of human plasma using tandem mass tags to identify novel biomarkers for herpes zoster. J Proteomics. (2020) 225:103879. 10.1016/j.jprot.2020.103879 32585426

[20] Walker JM. The Bradford method for protein quantitation. In: Walker JM. (eds), The Protein Protocols Handjournal. Springer Protocols Handjournals. Totowa, NJ: Humana Press. (2009). p. 17–24. 10.1007/978-1-59745-198-7_4

[21] Zhao C Bai Y Fu S Wu L Xu C Xia C. Follicular fluid proteomic profiling of dairy cows with anestrus caused by negative energy balance. Ital J Anim Sci. (2021) 20:650–63. 10.1080/1828051X.2021.1899855

[22] Gouwy M Struyf S Mahieu F Put W Proost P Van Damme J. The unique property of the CC chemokine regakine-1 to synergize with other plasma-derived inflammatory mediators in neutrophil chemotaxis does not reside in its NH2-terminal structure. Mol Pharmacol. (2002) 62:173–80. 10.1124/mol.62.1.173 12065768

[23] Parker AL Waddington SN Nicol CG Shayakhmetov DM Buckley SM Denby L. Multiple vitamin K-dependent coagulation zymogens promote adenovirus-mediated gene delivery to hepatocytes. Blood. (2006) 108:2554–61. 10.1182/blood-2006-04-008532 16788098

[24] Bukowska A Zacharias I Weinert S Skopp K Hartmann C Huth C. Coagulation factor Xa induces an inflammatory signalling by activation of protease-activated receptors in human atrial tissue. Eur J Pharmacol. (2013) 718:114–23. 10.1016/j.ejphar.2013.09.006 24041930

[25] Vedeler C Ulvestad E Bjørge L Conti G Williams K Mørk S. The expression of CD59 in normal human nervous tissue. Immunology. (1994) 82:542–7. 7530684 PMC1414919

[26] Eckersall PD Bell R. Acute phase proteins: Biomarkers of infection and inflammation in veterinary medicine. Vet J. (2010) 185:23–7. 10.1016/j.tvjl.2010.04.009 20621712

[27] Rahman MM Lecchi C Avallone G Roccabianca P Sartorelli P Ceciliani F. Lipopolysaccharide-binding protein: local expression in bovine extrahepatic tissues. Vet Immunol Immunopathol. (2010) 137:28–35. 10.1016/j.vetimm.2010.04.006 20452064

[28] Schumann RR Leong SR Flaggs GW Gray PW Wright SD Mathison JC. Structure and function of lipopolysaccharide binding protein. Science. (1990) 249:1429–429 10.1126/science.2402637 2402637

[29] Beer MCD Yuan T Kindy MS Asztalos BF Roheim PS Beer FCD. Characterization of constitutive human serum amyloid A protein (SAA4) as an apolipoprotein. J Lipid Res. (1995) 36:526opro 7775864

[30] Husby G Marhaug G Dowtor B Sletten K Sipe JD. Serum amyloid A (SAA): Biochemistry, genetics and the pathogenesis of AA amyloidosis. Amyloid. (2009) 1:119–37. 10.3109/13506129409148635

[31] Yamada T Kluve-Beckerman B Kuster WM Liepnieks JJ Benson MD. Measurement of serum amyloid A4 (SAA4): Its constitutive presence in serum. Amyloid. (1994) 1:114–8. 10.3109/13506129409148634

[32] Stavenuiter F Ebberink EHTM Mertens K Meijer AB. Role of glycine 221 in catalytic activity of hyaluronan-binding protein 2. J Biol Chem. (2017) 292:6381–38 10.1074/jbc.M116.757849 28246168 PMC5391765

[33] Kim E Li K Lieu C Tong S Kawai S Fukutomi T. Expression of apolipoprotein C-IV is regulated by Ku antigen/peroxisome proliferator-activated receptor gamma complex and correlates with liver steatosis. J Hepatol. (2008) 49:787lis. 10.1016/j.jhep.2008.06.029 18809223 PMC2644636

[34] Mackness MI Mackness B Durrington PN Connelly PW Hegele RA. Paraoxonase: biochemistry, genetics and relationship to plasma lipoproteins. Curr Opin Lipidol. (1996) 7:6996lt 10.1097/00041433-199604000-00004 8743898

[35] Weismann D Hartvigsen K Lauer N Bennett KL Scholl HP Charbel Issa P. Complement factor H binds malondialdehyde epitopes and protects from oxidative stress. Nature. (2011) 478:76.ved 10.1038/nature10449 21979047 PMC4826616

[36] Hamm A Veeck J Bektas N Wild PJ Hartmann A Heindrichs U. Frequent expression loss of Inter-alpha-trypsin inhibitor heavy chain (ITIH) genes in multiple human solid tumors: a systematic expression analysis. BMC Cancer. (2008) 8:25. 10.1186/1471-2407-8-25 18226209 PMC2268946

[37] Katoh N. Relevance of apolipoproteins in the development of fatty liver and fatty liver-related peripartum diseases in dairy cows. J Vet Med Sci. (2002) 64:293. 10.1292/jvms.64.293 12014573

[38] Katoh N. In addition to the high-density lipoprotein fraction, apolipoprotein C-III is detected in chylomicrons and the very low-density lipoprotein fraction from serum of normolipidemic cows. J Vet Med Sci. (2001) 63:95. 10.1292/jvms.63.95 11217073

[39] Yamamoto M Katoh N Adachi Y Oikawa S. Identification and purification of apolipoprotein C-III from the serum of cows. Am J Vet Res. (1998) 59:667opro 10.1007/s001220000455 9622732

[40] Yamamoto M Katoh N Oikawa S. Evaluation of serum apolipoprotein C-III concentration by enzyme-linked immunosorbent assay and its higher concentration in cows during midlactation than during the nonlactating stage. Am J Vet Res. (1998) 59:1358–358 10.1016/S0165-2427(98)00189-5 9829390

[41] Yamamoto M Nakagawa-Ueta H Katoh N Oikawa S. Decreased concentration of serum apolipoprotein C-III in cows with fatty liver, ketosis, left displacement of the abomasum, milk fever and retained placenta. J Vet Med Sci. (2001) 63:227ya.e 10.1292/jvms.63.227 11307920

[42] Csoka AB Frost GI Stern R. The six hyaluronidase-like genes in the human and mouse genomes. Matrix Biol. (2001) 20: 499–508. 10.1016/s0945-053x(01)00172-x 11731267

[43] FolnoŽić I Turk R Ðuričić D Vince S Pleadin J Flegar-Meštrić Z. Influence of body condition on serum metabolic indicators of lipid mobilization and oxidative stress in dairy cows during the transition period. Reprod Domest Anim. (2015) 50:910–7. 10.1111/rda.12608 26403271

[44] Seternes OM Kidger AM Keyse SM. Dual-specificity MAP kinase phosphatases in health and disease. Biochim Biophys Acta Mol Cell Res. (2019) 1866:124–43. 10.1016/j.bbamcr.2018.09.002 30401534 PMC6227380

[45] Saklatvala J. The p38 MAP kinase pathway as a therapeutic target in inflammatory disease. Curr Opin Pharmacol. (2004) 4:372–7. 10.1016/j.coph.2004.03.009 15251131

[46] Joffre O Nolte MA Spörri R. Reis e Sousa C. Inflammatory signals in dendritic cell activation and the induction of adaptive immunity. Immunol Rev. (2010) 227:234–47. 10.1111/j.1600-065X.2008.00718.x 19120488

[47] Boraschi D Italiani P Weil S Martin MU. The family of the interleukin-1 receptors. Immunol Rev. (2018) 281:197–232. 10.1111/imr.12606 29248002

[48] Zhang H-F El-Naggar AM Cheng H Prudova A Delaidelli A He J-Z. IL1RAP augments cysteine metabolism and drives oxidative stress adaptation and lung metastasis in Ewing sarcoma. Cancer Res. (2020):6080-6080. 10.1158/1538-7445

[49] Orlean P Menon AK. Thematic review series: lipid posttranslational modifications. GPI anchoring of protein in yeast and mammalian cells, or: how we learned to stop worrying and love glycophospholipids. J Lipid Res. (2007) 48:993–1011. 10.1194/jlr.R700002-JLR200 17361015

[50] Menon AK. Glycosylphosphatidylinositol anchors. In: Lennarz WJ, Lane MD, editors. Encyclopedia of Biological Chemistry. London: Academic Press. (2013): 476–8. 10.1016/B978-0-12-378630-2.00211-5

[51] Jones DR Avila MA Sanz C Varela-Nieto I. Glycosyl- phosphatidylinositol-phospholipase type D: a possible candidate for the generation of second messengers. Biochem Biophys Res Commun. (1997) 233:432–7. 10.1006/bbrc.1997.6475 9144552

[52] Rhode H Lopatta E Schulze M Pascual C Schulze HP Schubert K. Glycosylphosphatidylinositol-specific phospholipase D in blood serum: is the liver the only source of the enzyme?. Clin Chim Acta. (1999) 281:127–45. 10.1016/S0009-8981(98)00218-6 10217634

[53] GPI PLD. In: Schwab M, editors. Encyclopedia of Cancer. Berlin; Heidelberg: Springer (2011). 1587 p. 10.1007/978-3-642-16483-5_2489

[54] Hoener MC Brodbeck U. Phosphatidylinositol-glycan-specific phospholipase D is an amphiphilic glycoprotein that in serum is associated with high density lipoproteins. Eur J Biochem. (2010) 206:747–57. 10.1111/j.1432-1033.1992.tb16981.x 1606959

[55] Ragaller V Lebzien P Südekum KH Hüther L Flachowsky G. Pantothenic acid in ruminant nutrition: a review. J Anim Physiol Anim Nutr. (2011) 95:6–16. 10.1111/j.1439-0396.2010.01004.x 20579186

[56] Ball GFM. Vitamins in Foods: Analysis. Bioavailability, and Stability. Boca Raton, FL: CRC Press (2005).

[57] Chen S Zhang W Tang C Tang X Liu L Liu C. Vanin-1 is a key activator for hepatic gluconeogenesis. Diabetes. (2014) 63:2073–85. 10.2337/db13-0788 24550194

[58] Pitari G Malergue F Martin F Philippe JM Massucci MT Chabret C. Pantetheinase activity of membrane-bound Vanin-1: lack of free cysteamine in tissues of vanin-1 deficient mice. FEBS Lett. (2000) 483:149–54. 10.1016/S0014-5793(00)02110-4 11042271

[59] Maras B Barra D Duprè S. Pitari G. Is pantetheinase the actual identity of mouse and human vanin-1 proteins? Febs Letters. (1999) 461:149–52. 10.1016/S0014-5793(99)01439-8 10567687

[60] Hayes JD Mclellan LI. Glutathione and glutathione-dependent enzymes represent a coordinately regulated defence against oxidative stress. Free Radic Res Commun. (1999) 31:273–300. 10.1080/10715769900300851 10517533

[61] Berruyer C Martin FM Castellano R Macone A Malergue F Garrido-Urbani S. Vanin-1–/– mice exhibit a glutathione-mediated tissue resistance to oxidative stress. Mol Cell Biol. (2004) 24:7214–24. 10.1128/MCB.24.16.7214-7224.2004 15282320 PMC479710

[62] Baldi A. Vitamin E in dairy cows. Livestock Product Sci. (2005) 98:117–22. 10.1016/j.livprodsci.2005.10.004

[63] Spears JW Weiss WP. Role of antioxidants and trace elements in health and immunity of transition dairy cow. Vet J. (2008) 176:70–6. 10.1016/j.tvjl.2007.12.015 18325801

[64] Joy M Chakraborty K. Antioxidative and anti-inflammatory pyranoids and isochromenyl analogues from Corbiculid bivalve clam, Villorita cyprinoides. Food Chem. (2018) 251:125–34. 10.1016/j.foodchem.2018.01.059 29426418

[65] Ziouzenkova O Plutzky J. Lipolytic PPAR activation: new insights into the intersection of triglycerides and inflammation? Curr Opin Clin Nutr Metab Care. (2004) 7:369. 10.1097/01.mco.0000134358.46159.61 15192437

[66] Moffit JS Koza-Taylor PH Holland RD Thibodeau MS Beger RD Lawton MP. Differential gene expression in mouse liver associated with the hepatoprotective effect of clofibrate. Toxicol Appl Pharmacol. (2007) 222:169–79. 10.1016/j.taap.2007.04.008 17585979 PMC1989769

[67] Rommelaere S Millet V Gensollen T Bourges C Eeckhoute J Hennuyer N. PPARalpha regulates the production of serum Vanin-1 by liver. FEBS Lett. (2013) 587:3742–8. 10.1016/j.febslet.2013.09.046 24140347

[68] Rakhsh M Knoch B Müller M Kersten E. Peroxisome proliferator-activated receptor alpha target genes. PPAR Res. (2010) 2010:393–416. 10.1155/2010/612089 20936127 PMC2948931

[69] Evans JL Goldfine ID Maddux BA Grodsky GM. Oxidative stress and stress-activated signaling pathways: a unifying hypothesis of type 2 diabetes. Endocr Rev. (2002) 23:599–622. 10.1210/er.2001-0039 12372842

[70] Tan KS Lee KO Low KC Gamage AM Liu Y Tan GYG. Glutathione deficiency in type 2 diabetes impairs cytokine responses and control of intracellular bacteria. J Clin Invest. (2012) 122:2289. 10.1172/JCI57817 22546856 PMC3366396

