# Supplementary material for: Plasma Proteomics Characteristics of Subclinical Vitamin E Deficiency of Dairy Cows During Early Lactation
Source: Front Vet Sci. 2021 Dec 10;8:723898. doi: 10.3389/fvets.2021.723898 (PMC8703030; doi:10.3389/fvets.2021.723898)
Supplement: Supplementary file 1 [file Data_Sheet_1.pdf]

Supplementary Material

Supplementary Table 1 Dietary composition and nutritional level of experimental dairy cows in early postpartum period

**Item**	**EP**[1]
Ingredient (% of DM)	
Corn silage	19.61
Leymus chinensis	26.99
Concentrated feed	4.21
Corn	39.58
Soybean meal	8.48
Calcium hydrogen phosphate	0.22
Sodium bicarbonate	0.49
Salt	0.32
Vitamin and Se[2]	0.10
Main Chemical analysis	
DM (% as fed)	75.6
Net energy of lactation(Mcal/kg)	1.65

Crude protein(% of DM)	16.5
Crude fat(% of DM)	5.6
Calcium(% of DM)	0.65
Phosphorus(% of DM)	0.43
Magnesium(% of DM)	0.28
Potassium(% of DM)	0.71

[1]EP=The early postpartum period

[2]Provided (per kg of DM): 0.9 g of Se , 65000 IU/kg of vitamin A, 12700 IU of vitamin D, 300 IU/kg of vitamin E.

Supplementary Table 2 Detailed separation gradient of reversed phase chromatography at high pH

**Time（minute）**	**Mobile phase B[1] ratio（%）**
0	5
35	18
62	32
64	95
68	95
72	5

[1]Mobile phase B:98% acetonitrile, 2% ddH2O, pH 10.

Supplemental Table 3 Detailed separation gradient of nanoLC-MS/MS analysis

**Time（minute）**	**Mobile phase B[1] ratio（%）**
0	6
11	12
48	20
68	32
69	95
75	95

[1]Mobile phase B:100 % acetonitrile, 0.1 % formic acid.

Supplemental Table 4 Specific retrieval parameter Settings handled by Maxquant software

**Parameters**	**Value**
Enzyme	Trypsin
Static Modification	C carboxyamidomethylation（57.021 Da）
Dynamic Modification	M Oxidation（15.995Da）；N-terminal
Species	BOVIN
Precursor ion mass tolerance	± 15 ppm
Fragment ion mass tolerance	± 0.5 Da
Max Missed Cleavages	2

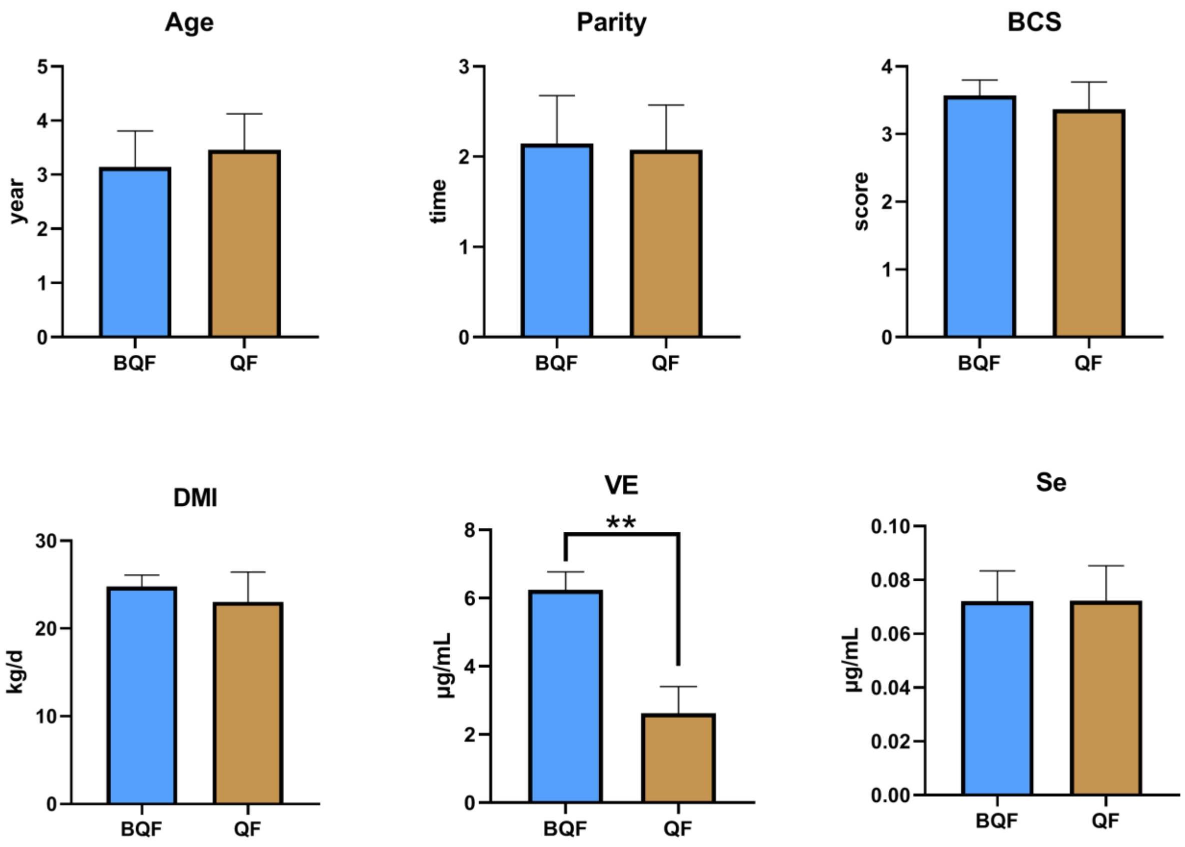

Supplementary Figures 1 The clinical characteristics of the subclinical VE deficiency group (QF) and normal control group (BQF) ,**P <0.05.
