# Supplementary material for: Plasma Proteomics Characteristics of Subclinical Vitamin E Deficiency of Dairy Cows During Early Lactation
Source: Front Vet Sci. 2021 Dec 10;8:723898. doi: 10.3389/fvets.2021.723898 (PMC8703030; doi:10.3389/fvets.2021.723898)
Supplement: Supplementary file 5 [file Data_Sheet_2.zip › Bioinformatics analysis related attachments/KEGG/Sample/bta00260.html]

Èç¹ûÒÔÏÂÍ¼Æ¬´ò¿ª³öÏÖÍ¼Æ¬²»È«µÈÎÊÌâ£¬ÇëÖ±½Ó´ÓKEGGÍøÉÏÏÂÔØ´ËÒ³Ãæ£¬µã»÷ÕâÀï¡£
