# Supplementary material for: Plasma Proteomics Characteristics of Subclinical Vitamin E Deficiency of Dairy Cows During Early Lactation
Source: Front Vet Sci. 2021 Dec 10;8:723898. doi: 10.3389/fvets.2021.723898 (PMC8703030; doi:10.3389/fvets.2021.723898)
Supplement: Supplementary file 5 [file Data_Sheet_2.zip › Bioinformatics analysis related attachments/KEGG/Sample/bta01100.html]

�������ͼƬ�򿪳���ͼƬ��ȫ�����⣬��ֱ�Ӵ�KEGG�������ش�ҳ�棬���������


 
 
 
 
 
 
 
 
 
 
 
 
 
 
 
 
 
 
 
 
 
 
 
 
 
 
 
 
 
 
 
 
 
 
 
 
 
 
 
 
 
 
 
 
 
 
 
 
 
 
 
 
 
 
 
 
 
 
 
 
 
 
 
 
 
 
 
 
 
 
 
 
 
 
 
 
 
 
 
 
 
 
 
 
 
 
 
 
 
 
 
 
 
 
 
 
 
 
 
 
 
 
 
 
 
 
 
 
 
 
 
 
 
 
 
 
 
 
 
 
 
 
 
 
 
 
 
 
 
 
 
 
 
 
 
 
 
 
 
 
 
 
 
 
 
 
 
 
 
 
 
 
 
 
 
 
 
 
 
 
 
 
 
 
 
 
 
 
 
 
 
 
 
 
 
 
 
 
 
 
 
 
 
 
 
 
 
 
 
 
 
 
 
 
 
 
 
 
 
 
 
 
 
 
 
 
 
 
 
 
 
 
 
 
 
 
 
 
 
 
 
 
 
 
 
 
 
 
 
 
 
 
 
 
 
 
 
 
 
 
 
 
 
 
 
 
 
 
 
 
 
 
 
 
 
 
 
 
 
 
 
 
 
 
 
 
 
 
 
 
 
 
 
 
 
 
 
 
 
 
 
 
 
 
 
 
 
 
 
 
 
 
 
 
 
 
 
 
 
 
 
 
 
 
 
 
 
 
 
 
 
 
 
 
 
 
 
 
