# Supplementary figures and images for: Plasma Proteomics Characteristics of Subclinical Vitamin E Deficiency of Dairy Cows During Early Lactation
Source: Front Vet Sci. 2021 Dec 10;8:723898. doi: 10.3389/fvets.2021.723898 (PMC8703030; doi:10.3389/fvets.2021.723898)

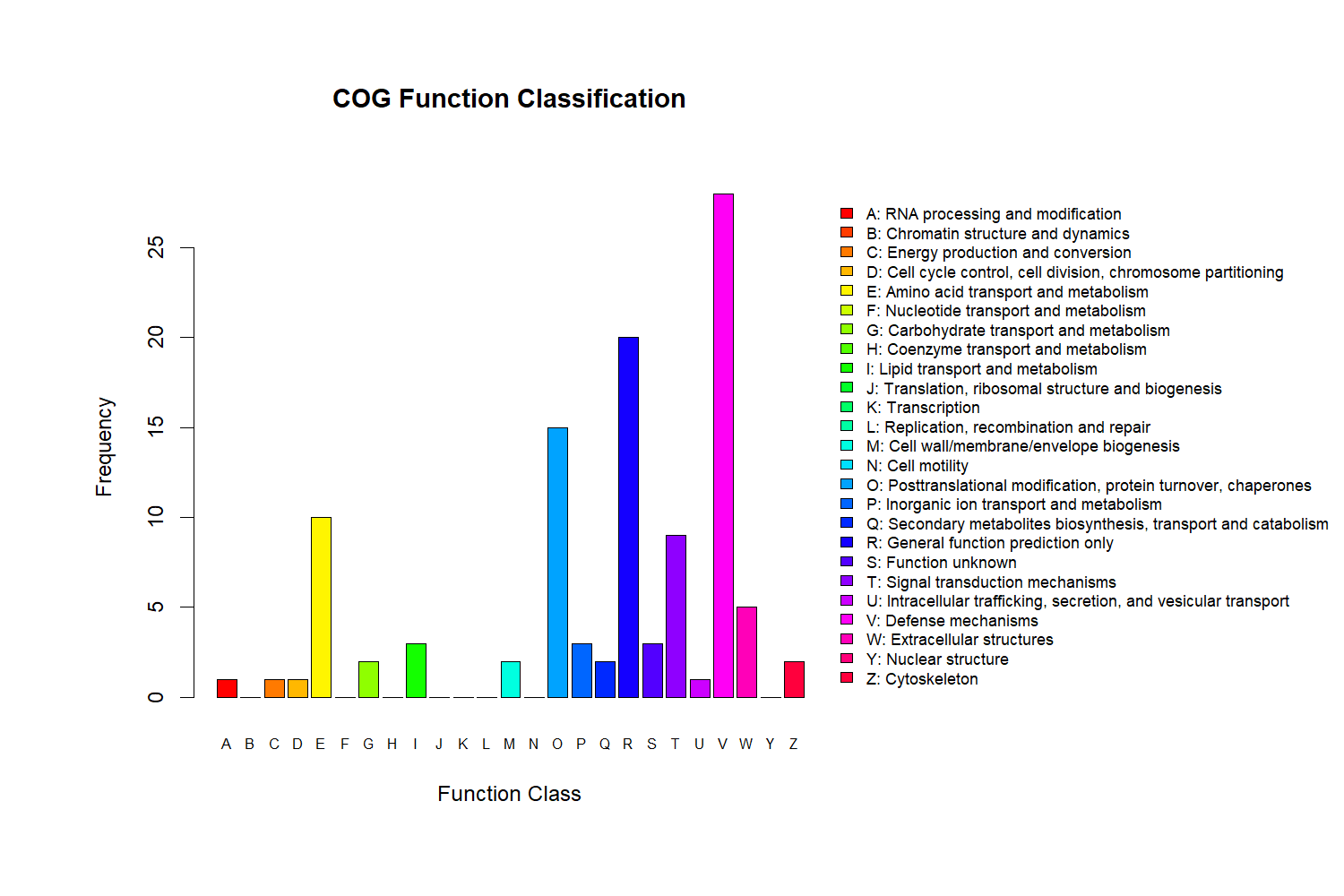

Supplement: Supplementary file 5 [file Data_Sheet_2.zip › Bioinformatics analysis related attachments/COG/cog.png]

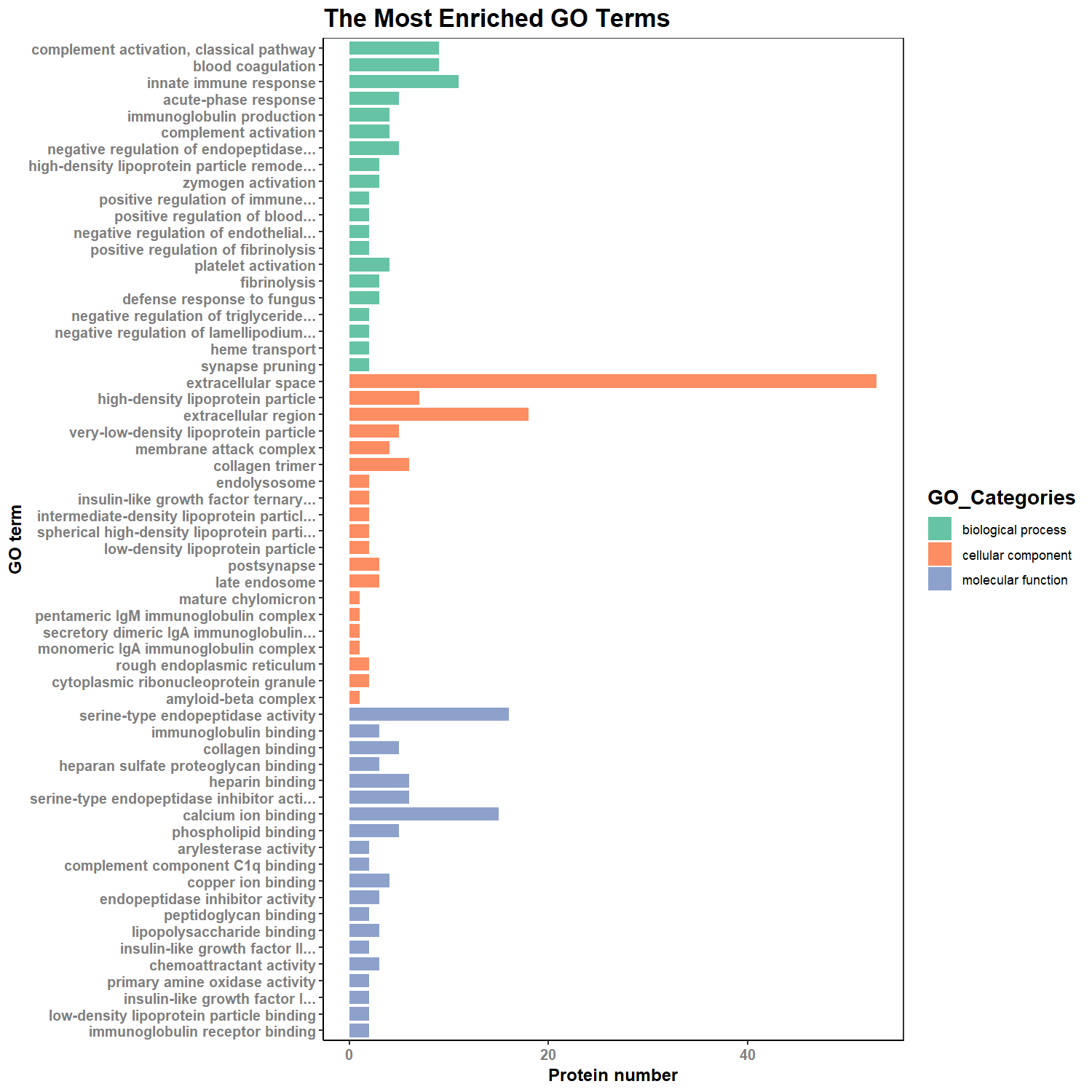

Supplement: Supplementary file 5 [file Data_Sheet_2.zip › Bioinformatics analysis related attachments/GO/Sample.EnrichedGOTerm.png]

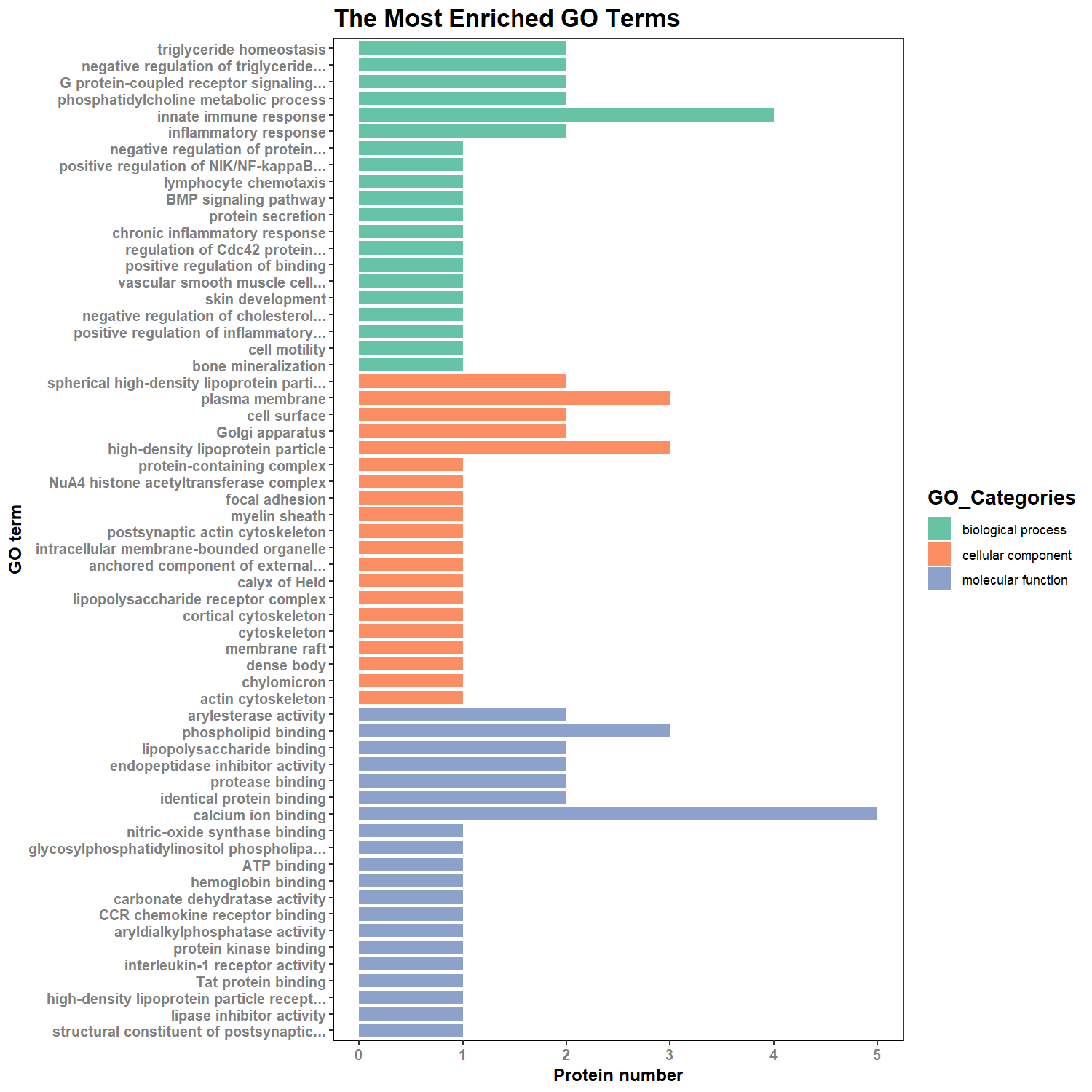

Supplement: Supplementary file 5 [file Data_Sheet_2.zip › Bioinformatics analysis related attachments/GO/Sig(QF_BQF).AllDiff.EnrichedGOTerm.png]

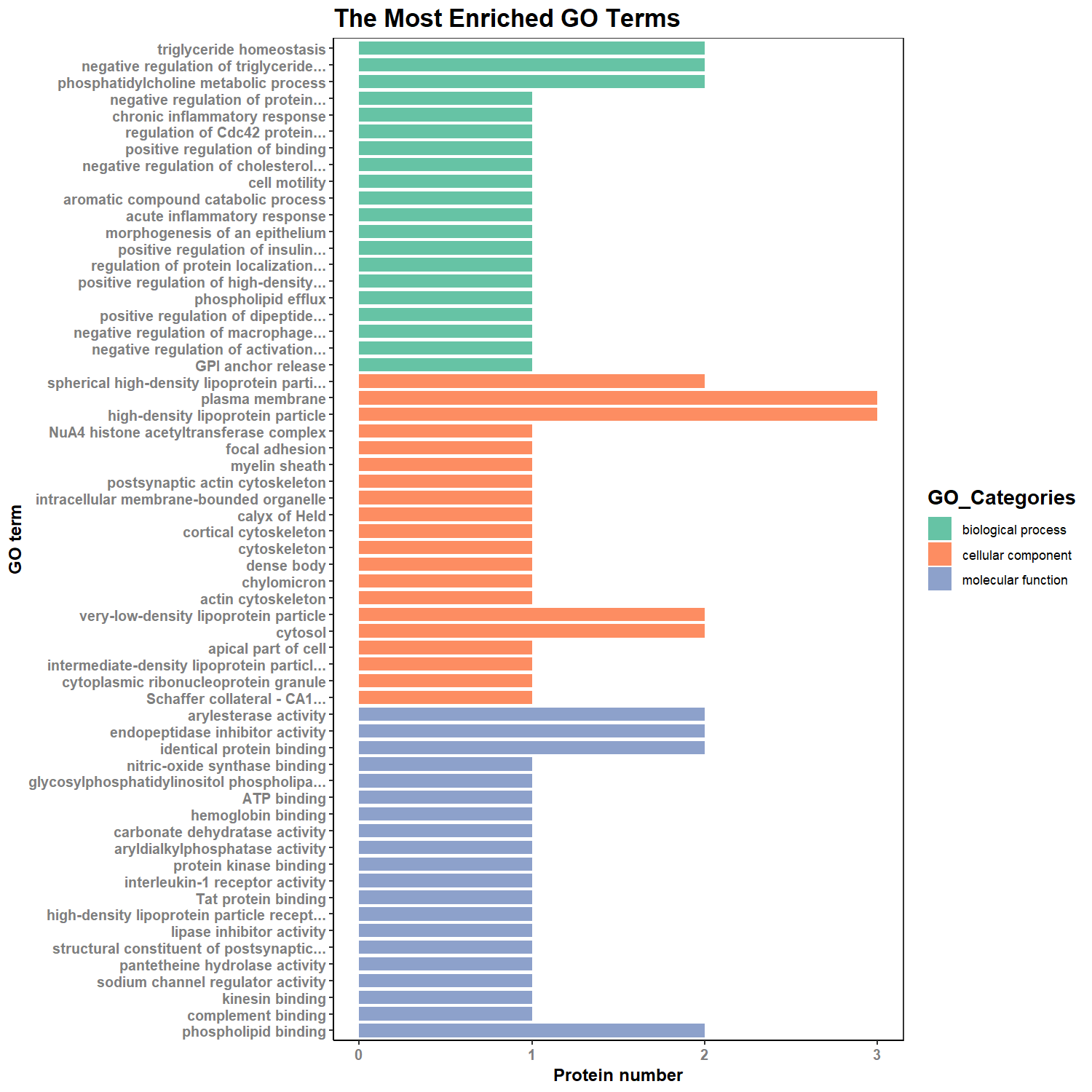

Supplement: Supplementary file 5 [file Data_Sheet_2.zip › Bioinformatics analysis related attachments/GO/Sig(QF_BQF).Down.EnrichedGOTerm.png]

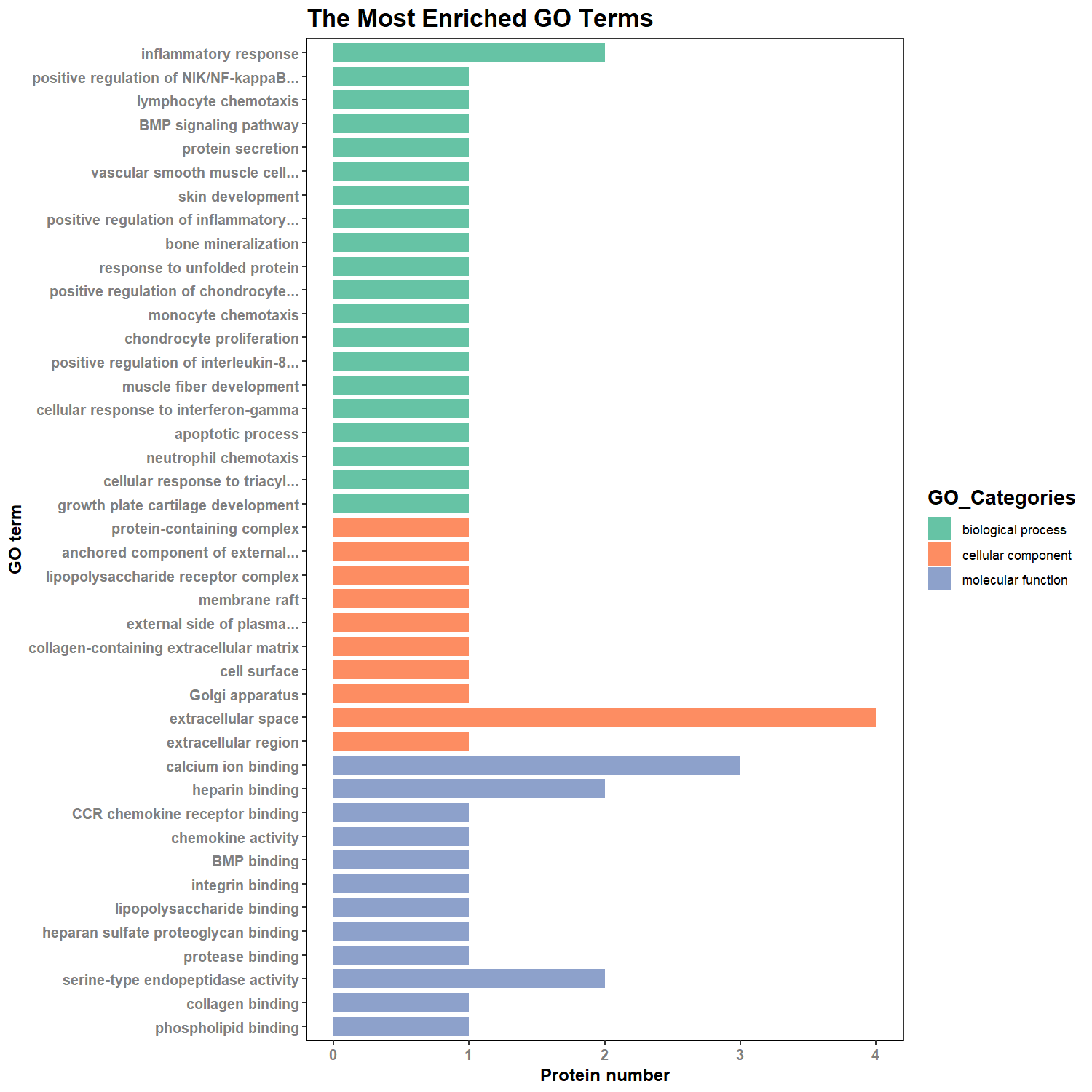

Supplement: Supplementary file 5 [file Data_Sheet_2.zip › Bioinformatics analysis related attachments/GO/Sig(QF_BQF).Up.EnrichedGOTerm.png]

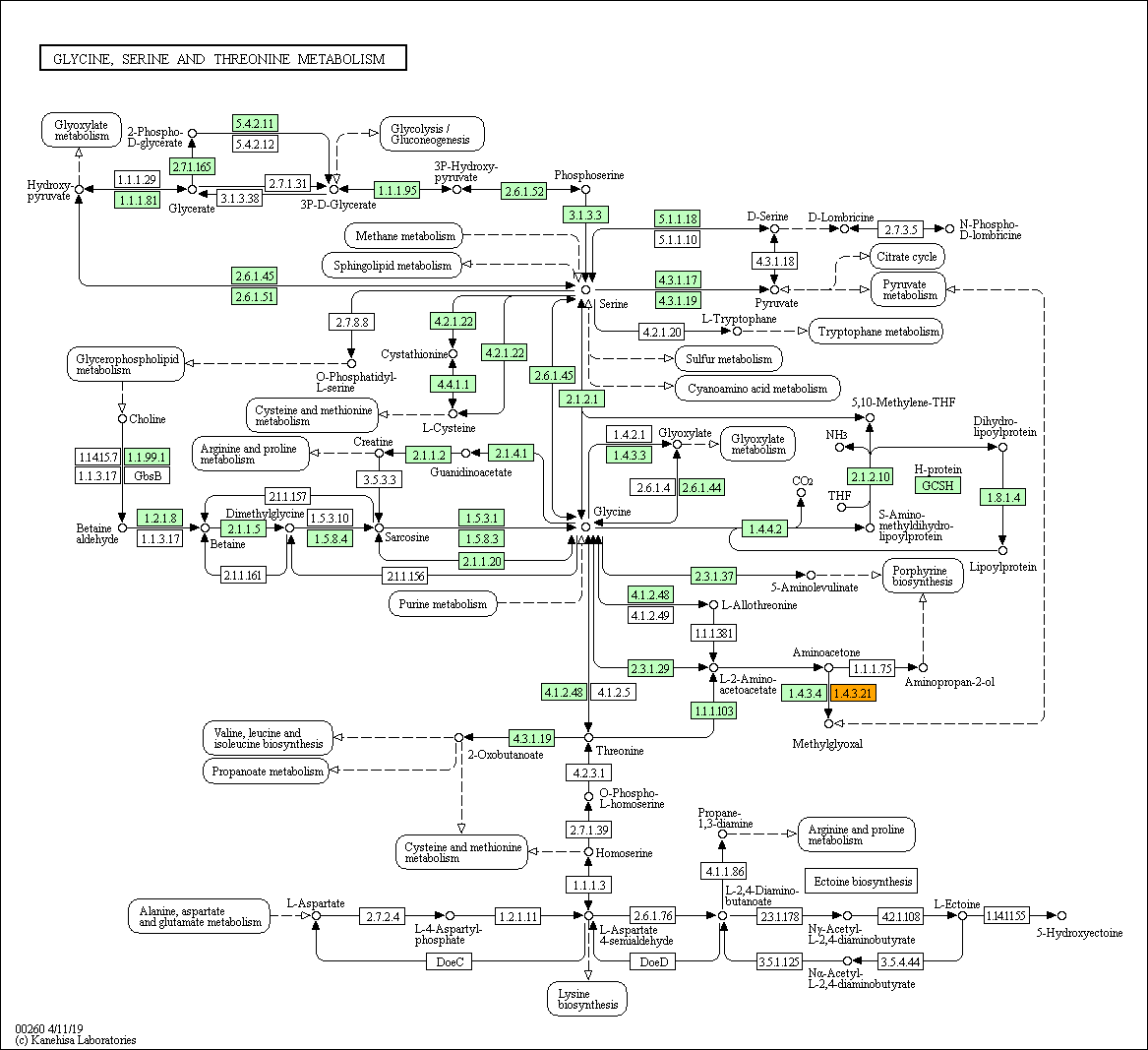

Supplement: Supplementary file 5 [file Data_Sheet_2.zip › Bioinformatics analysis related attachments/KEGG/Sample/png/bta00260.png]

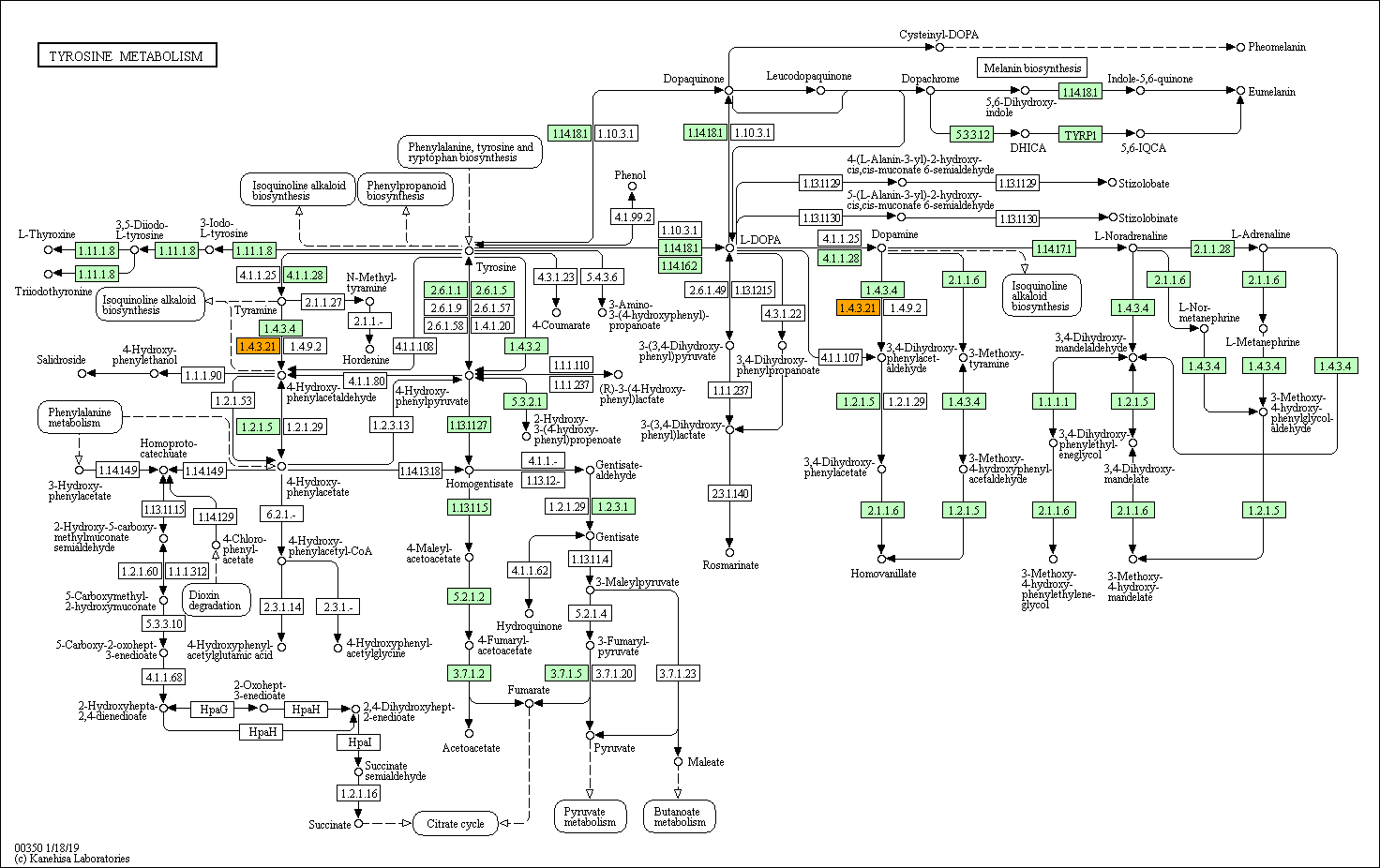

Supplement: Supplementary file 5 [file Data_Sheet_2.zip › Bioinformatics analysis related attachments/KEGG/Sample/png/bta00350.png]

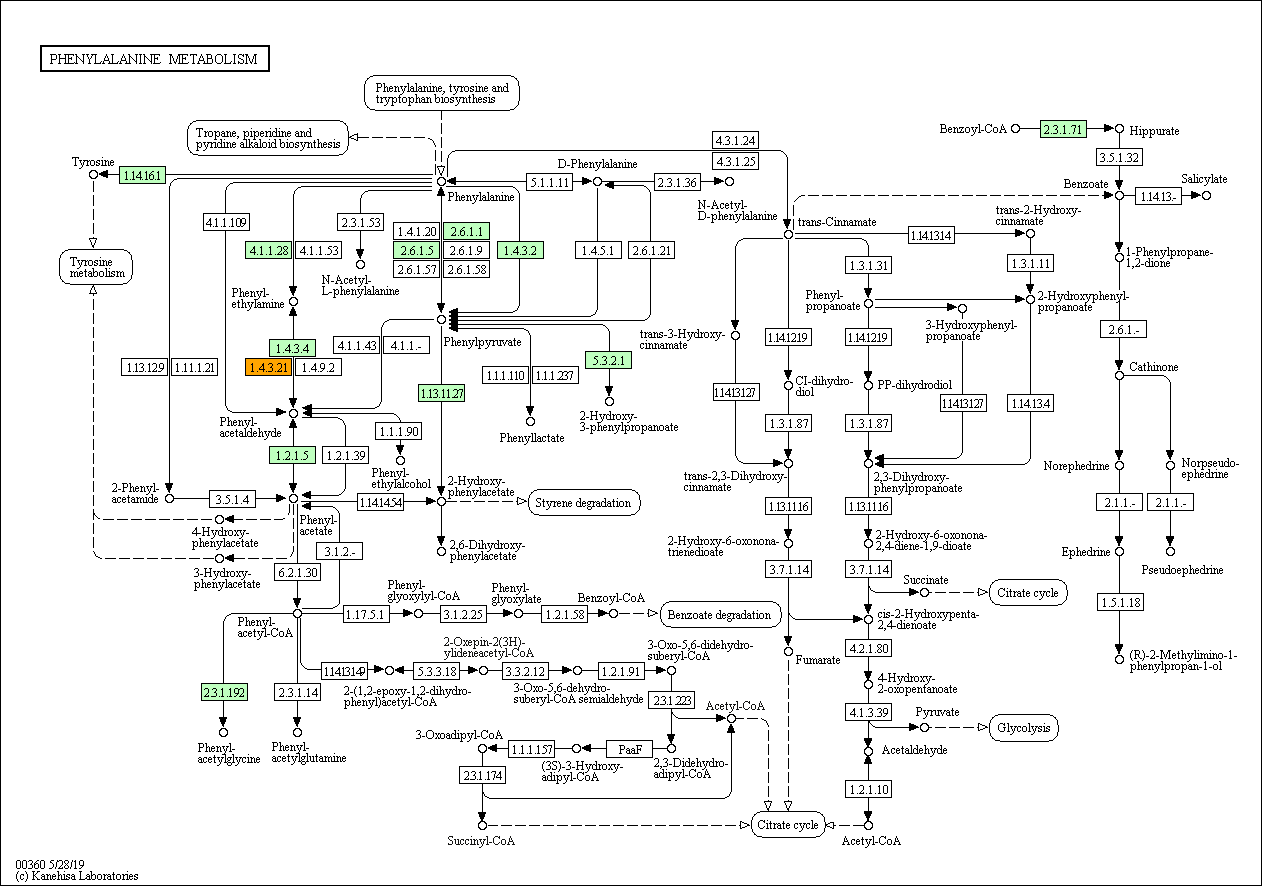

Supplement: Supplementary file 5 [file Data_Sheet_2.zip › Bioinformatics analysis related attachments/KEGG/Sample/png/bta00360.png]

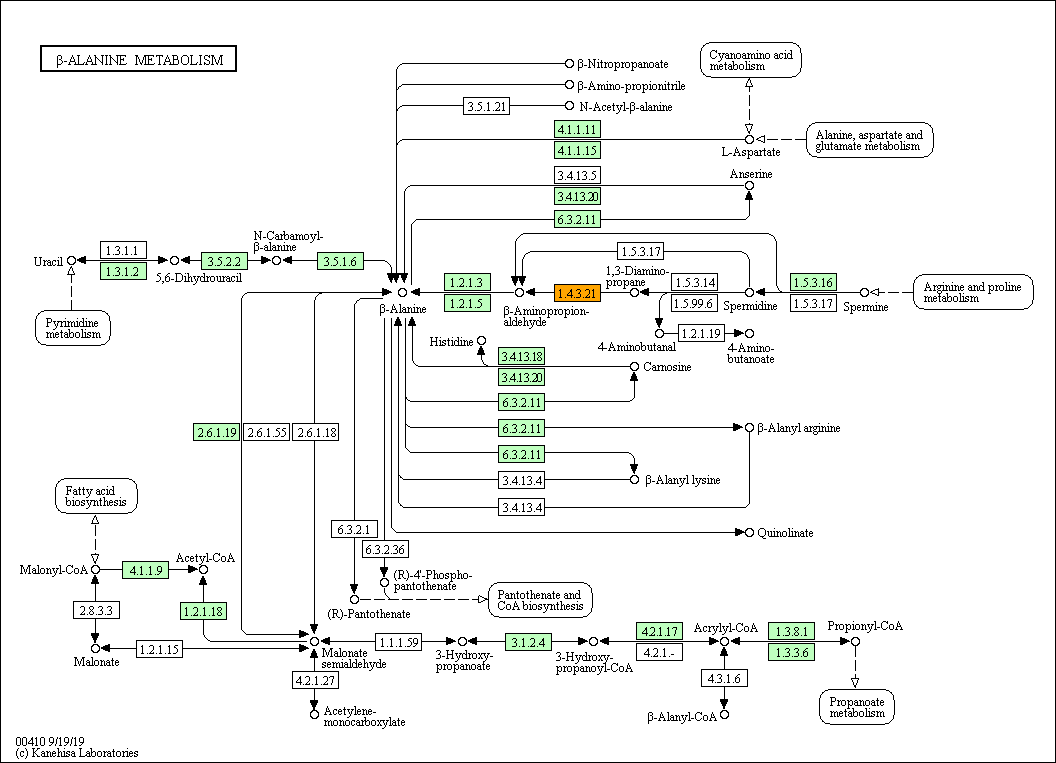

Supplement: Supplementary file 5 [file Data_Sheet_2.zip › Bioinformatics analysis related attachments/KEGG/Sample/png/bta00410.png]

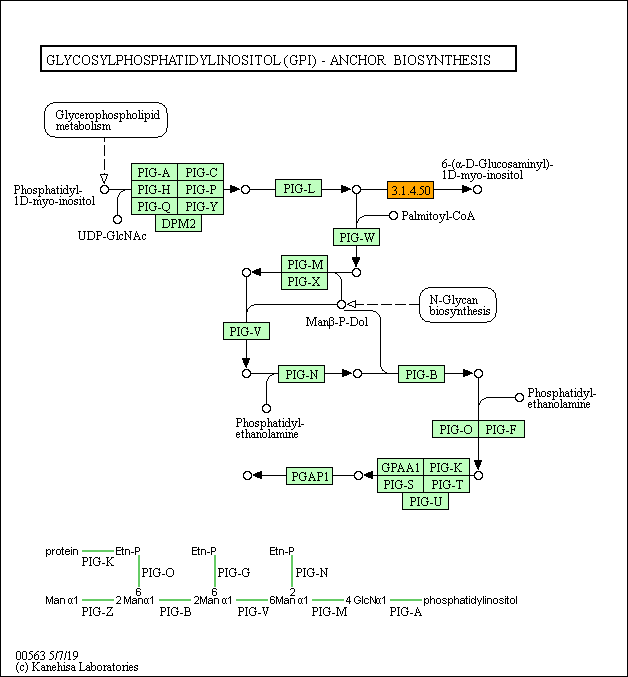

Supplement: Supplementary file 5 [file Data_Sheet_2.zip › Bioinformatics analysis related attachments/KEGG/Sample/png/bta00563.png]

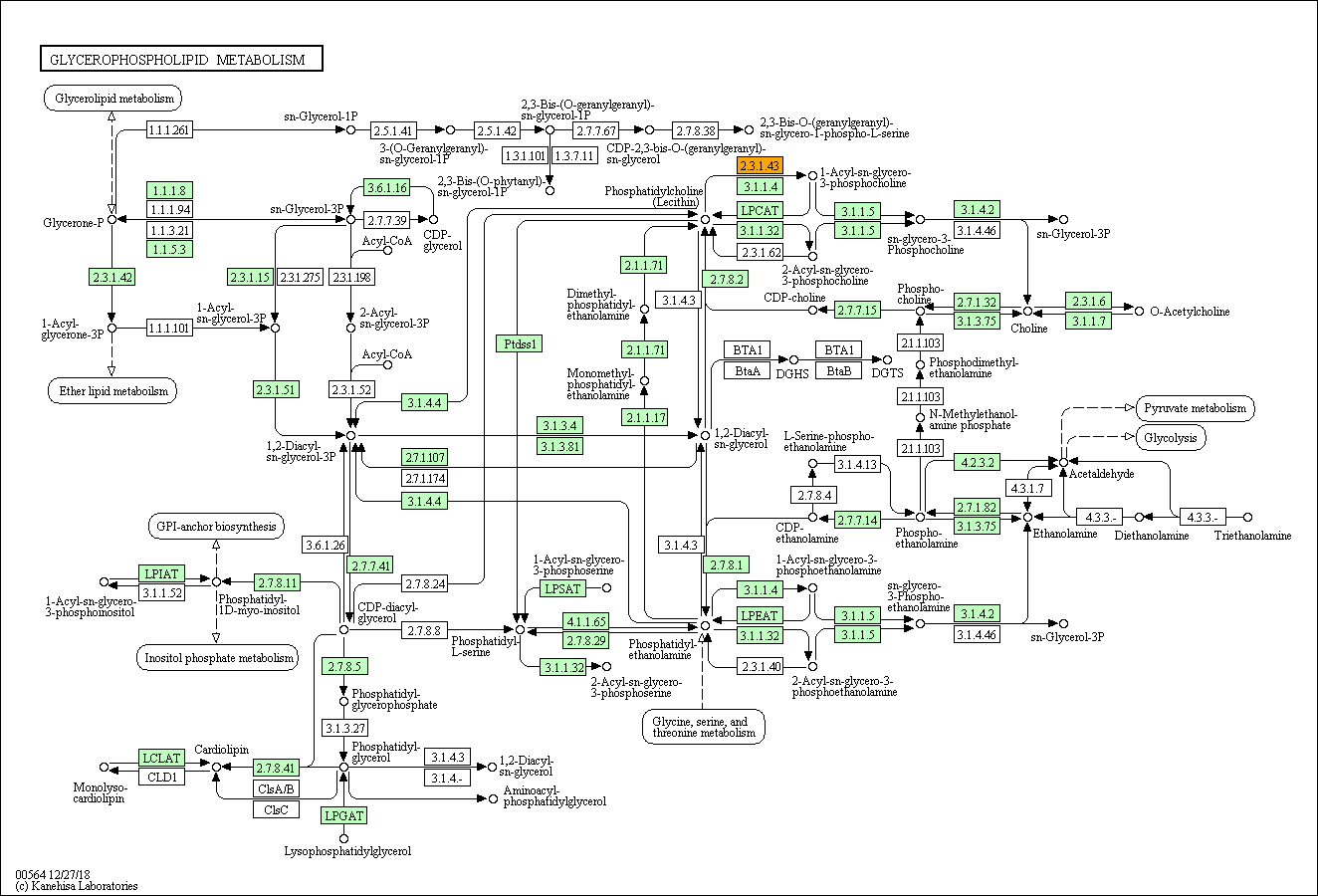

Supplement: Supplementary file 5 [file Data_Sheet_2.zip › Bioinformatics analysis related attachments/KEGG/Sample/png/bta00564.png]

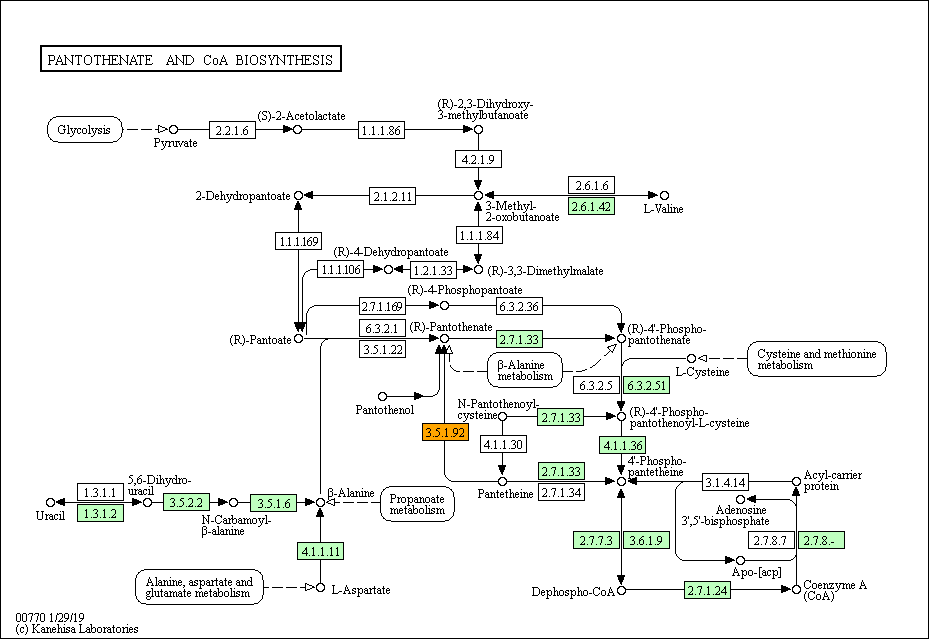

Supplement: Supplementary file 5 [file Data_Sheet_2.zip › Bioinformatics analysis related attachments/KEGG/Sample/png/bta00770.png]

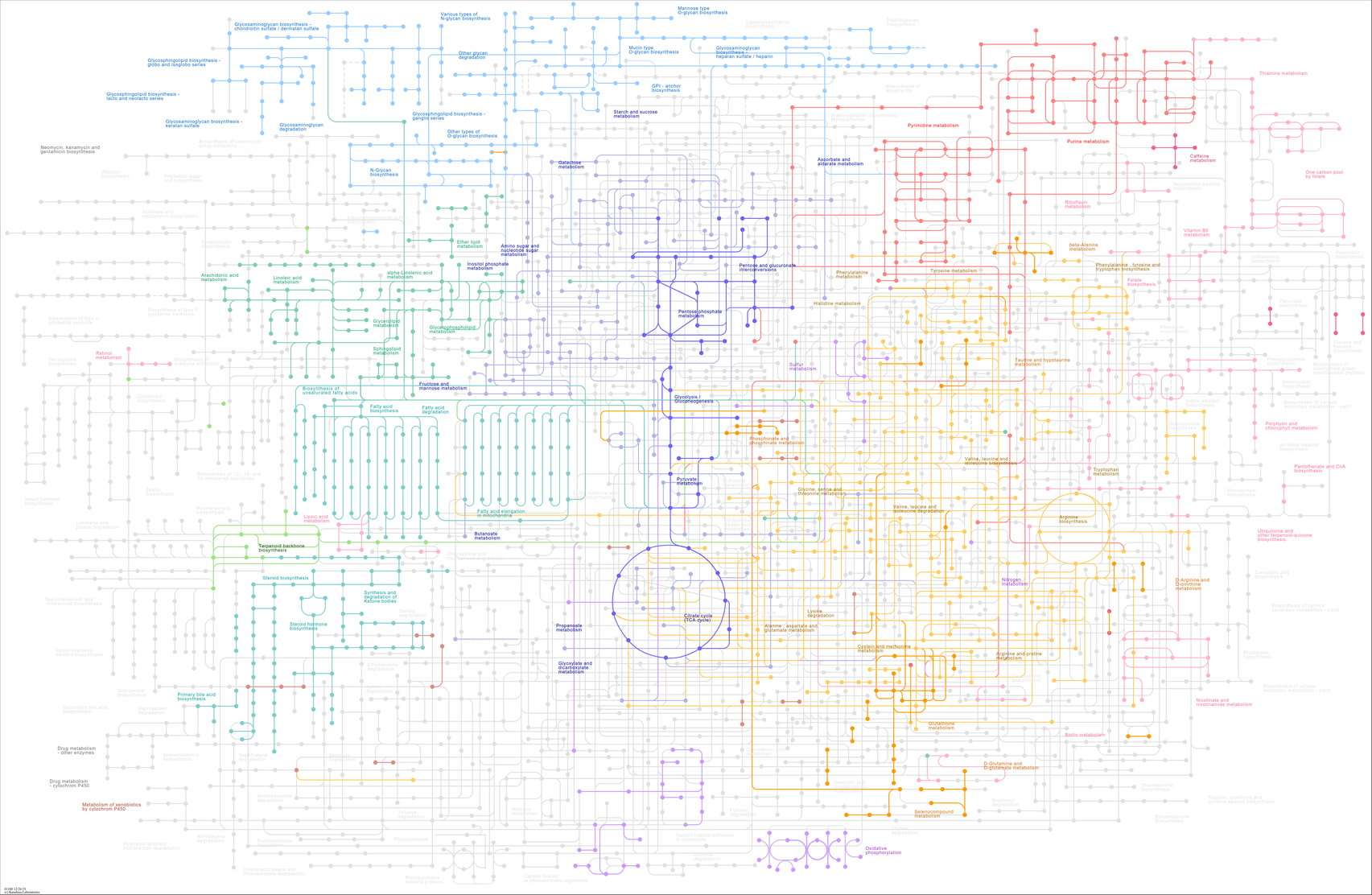

Supplement: Supplementary file 5 [file Data_Sheet_2.zip › Bioinformatics analysis related attachments/KEGG/Sample/png/bta01100.png]

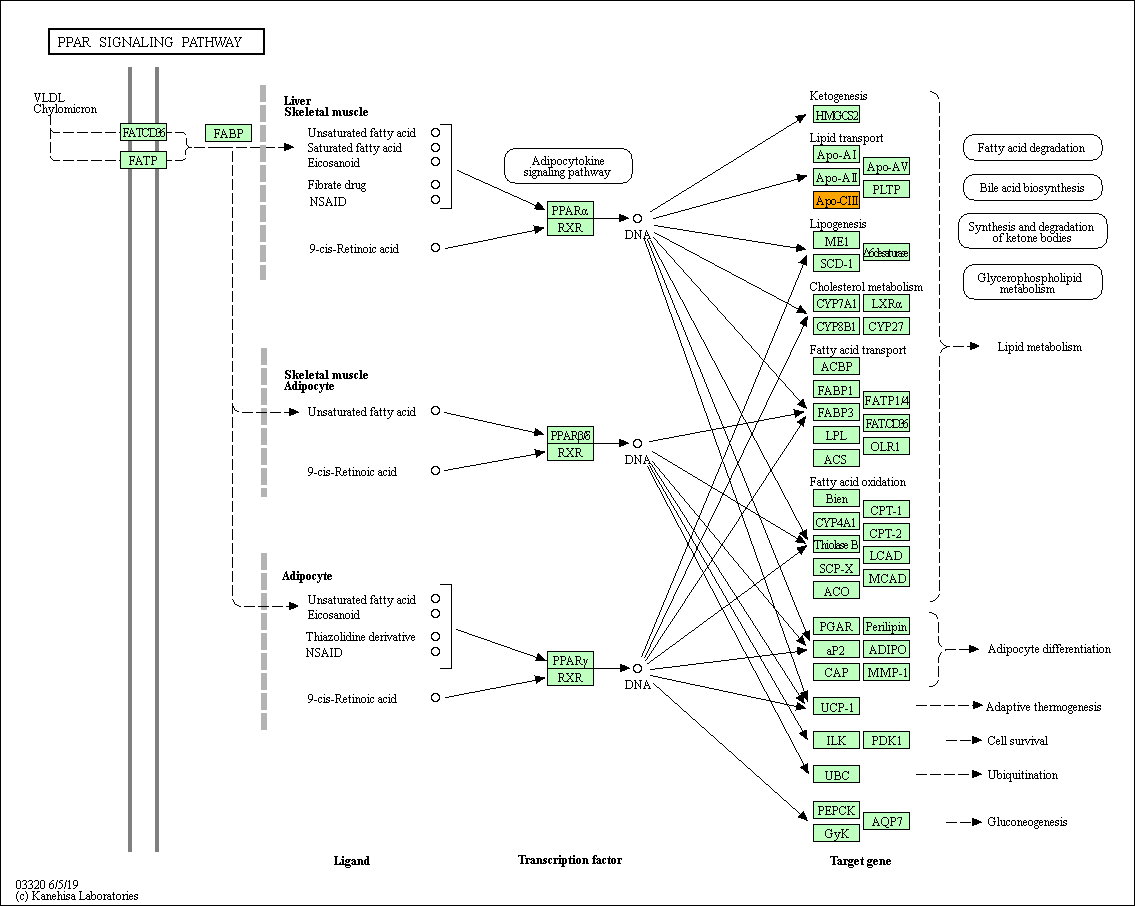

Supplement: Supplementary file 5 [file Data_Sheet_2.zip › Bioinformatics analysis related attachments/KEGG/Sample/png/bta03320.png]

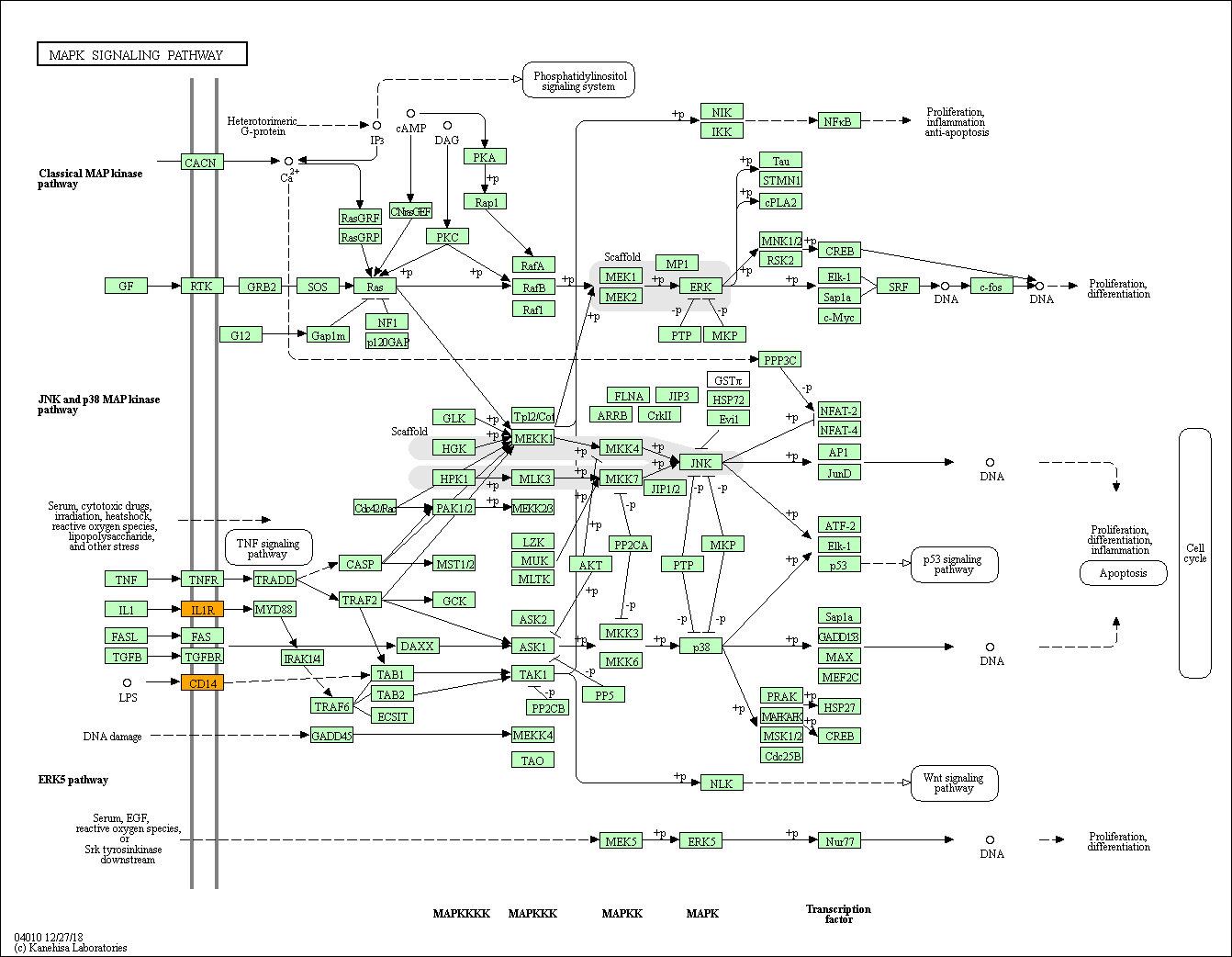

Supplement: Supplementary file 5 [file Data_Sheet_2.zip › Bioinformatics analysis related attachments/KEGG/Sample/png/bta04010.png]

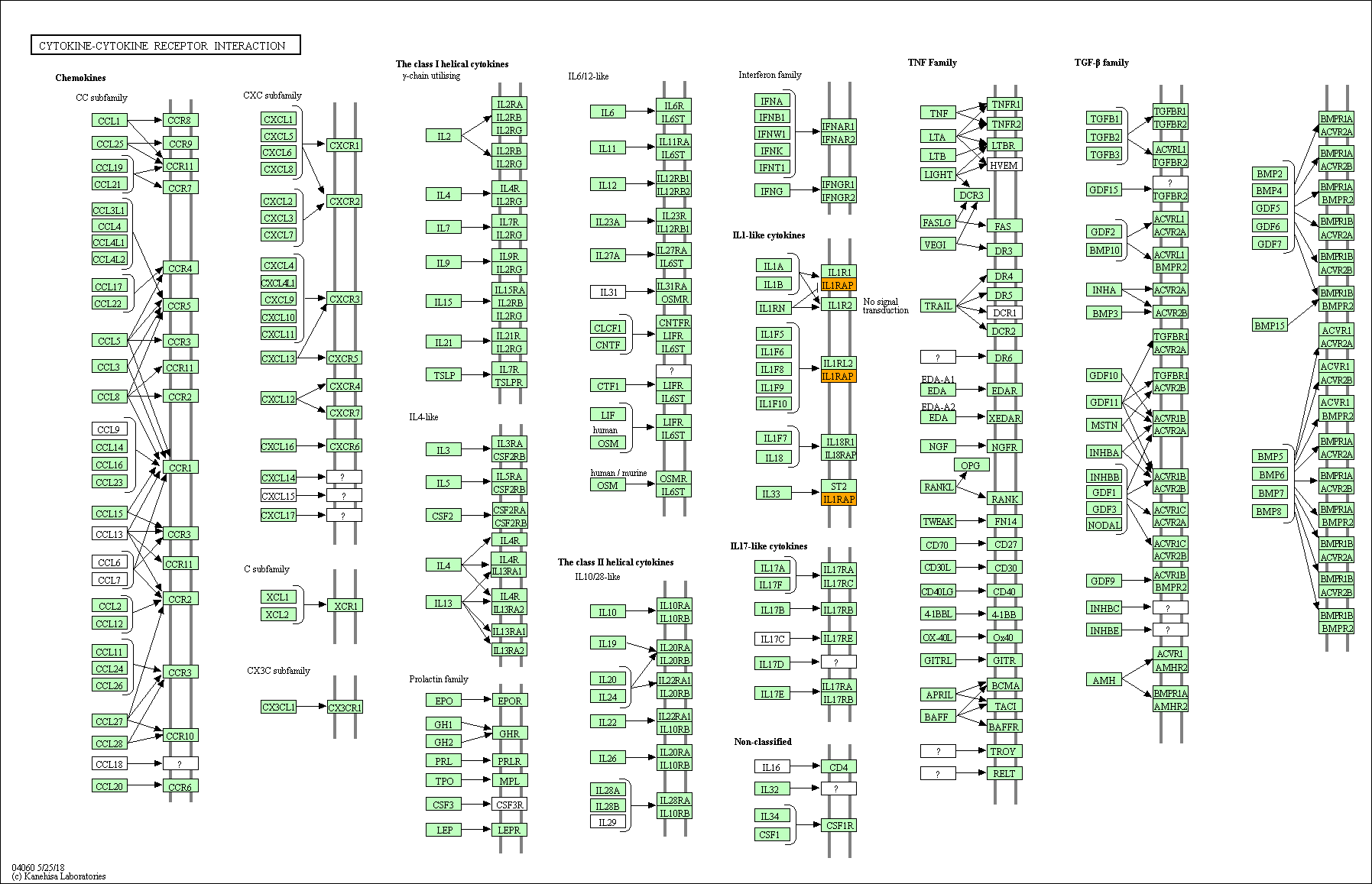

Supplement: Supplementary file 5 [file Data_Sheet_2.zip › Bioinformatics analysis related attachments/KEGG/Sample/png/bta04060.png]

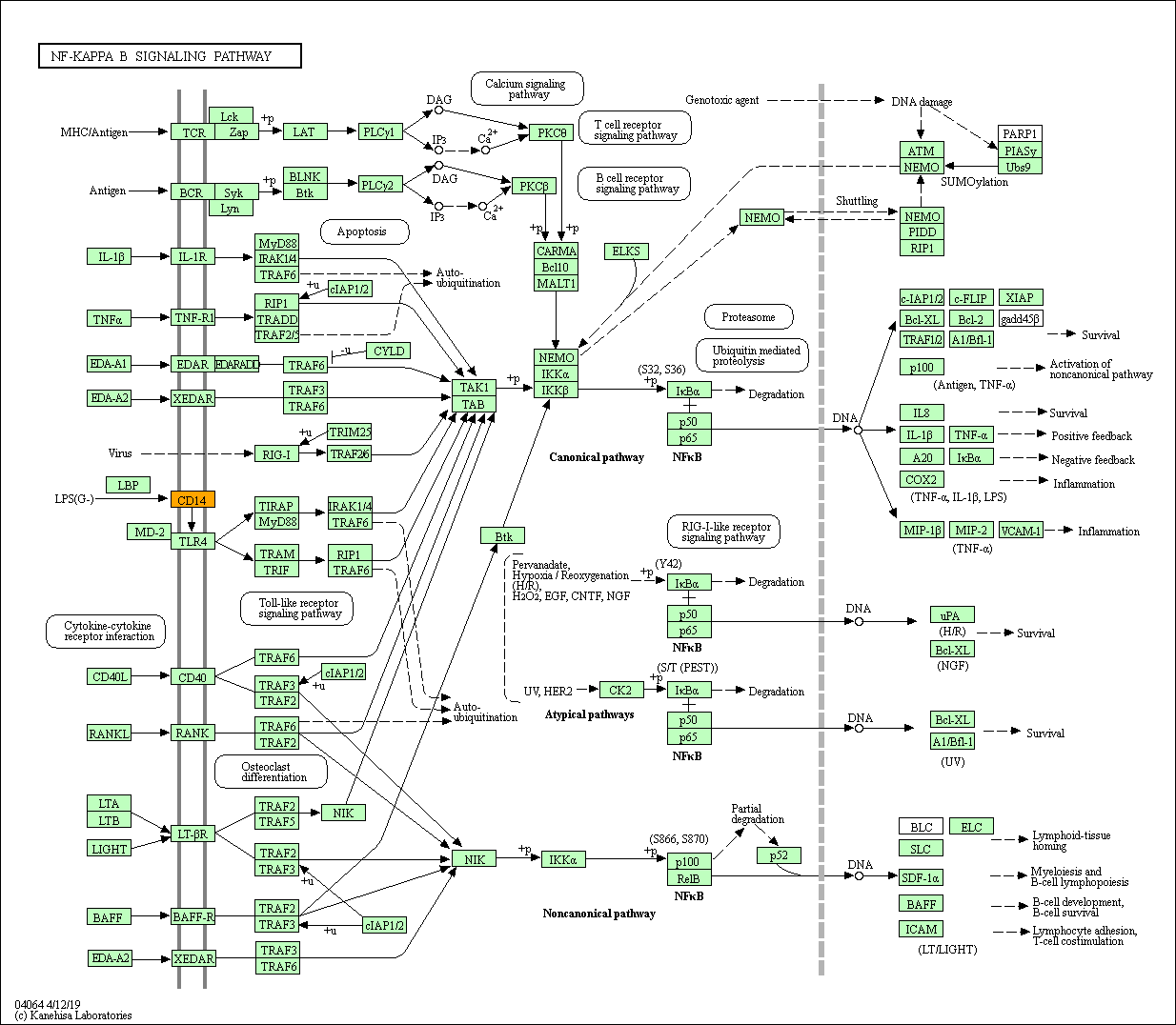

Supplement: Supplementary file 5 [file Data_Sheet_2.zip › Bioinformatics analysis related attachments/KEGG/Sample/png/bta04064.png]

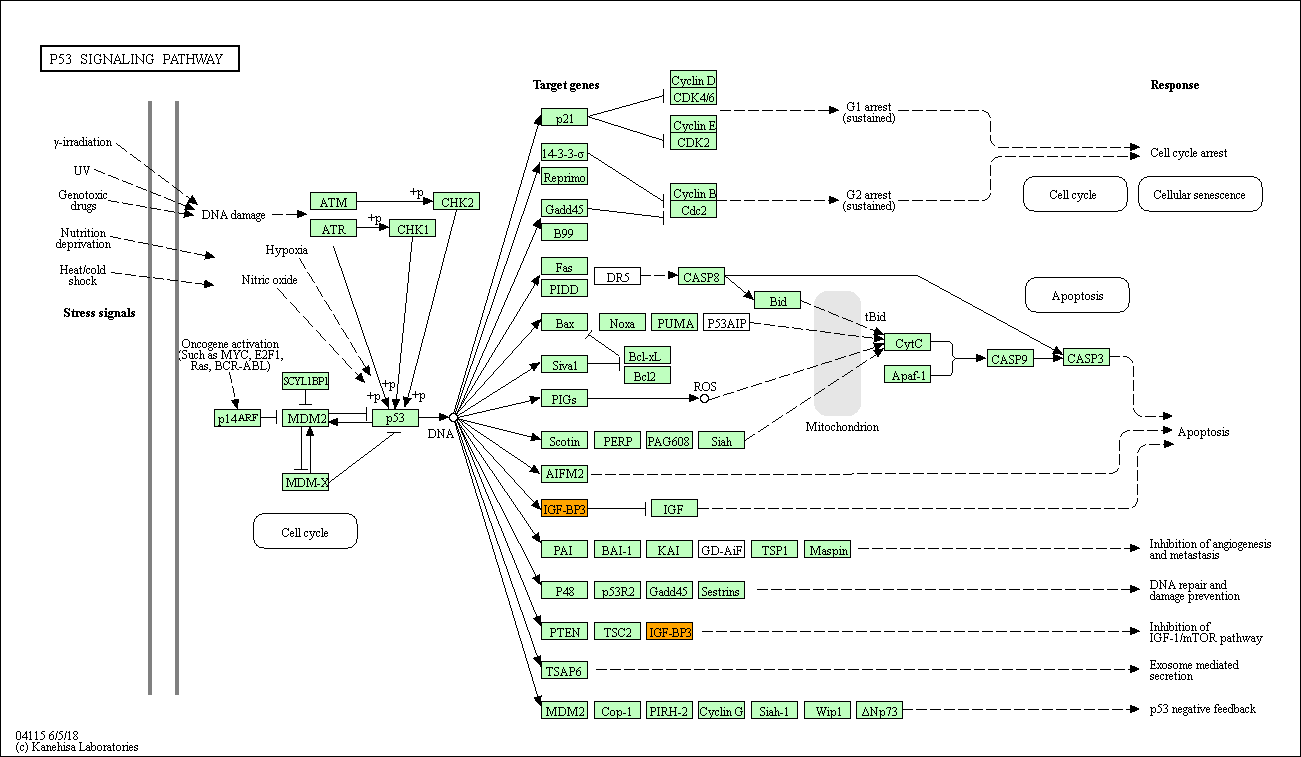

Supplement: Supplementary file 5 [file Data_Sheet_2.zip › Bioinformatics analysis related attachments/KEGG/Sample/png/bta04115.png]

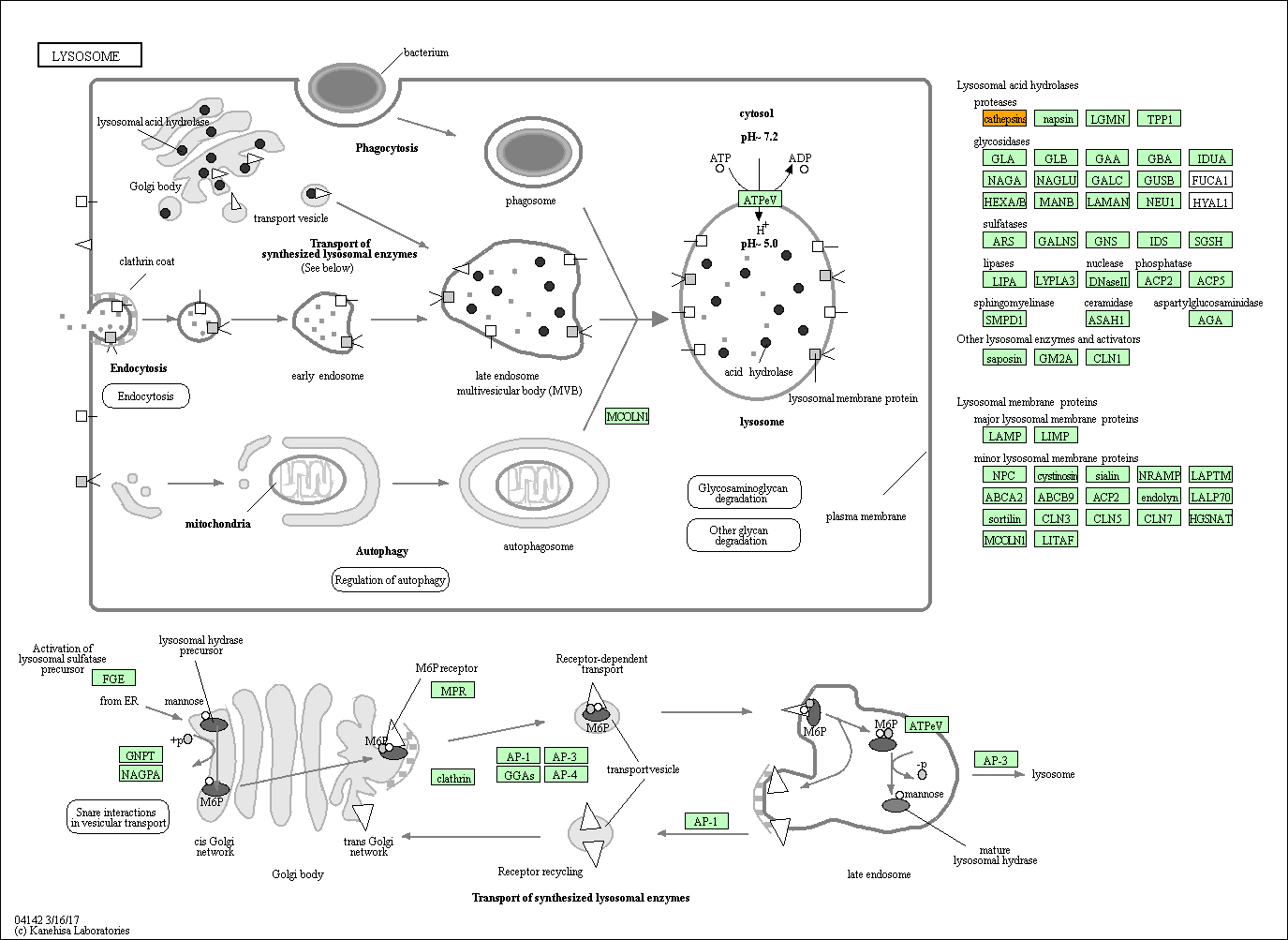

Supplement: Supplementary file 5 [file Data_Sheet_2.zip › Bioinformatics analysis related attachments/KEGG/Sample/png/bta04142.png]

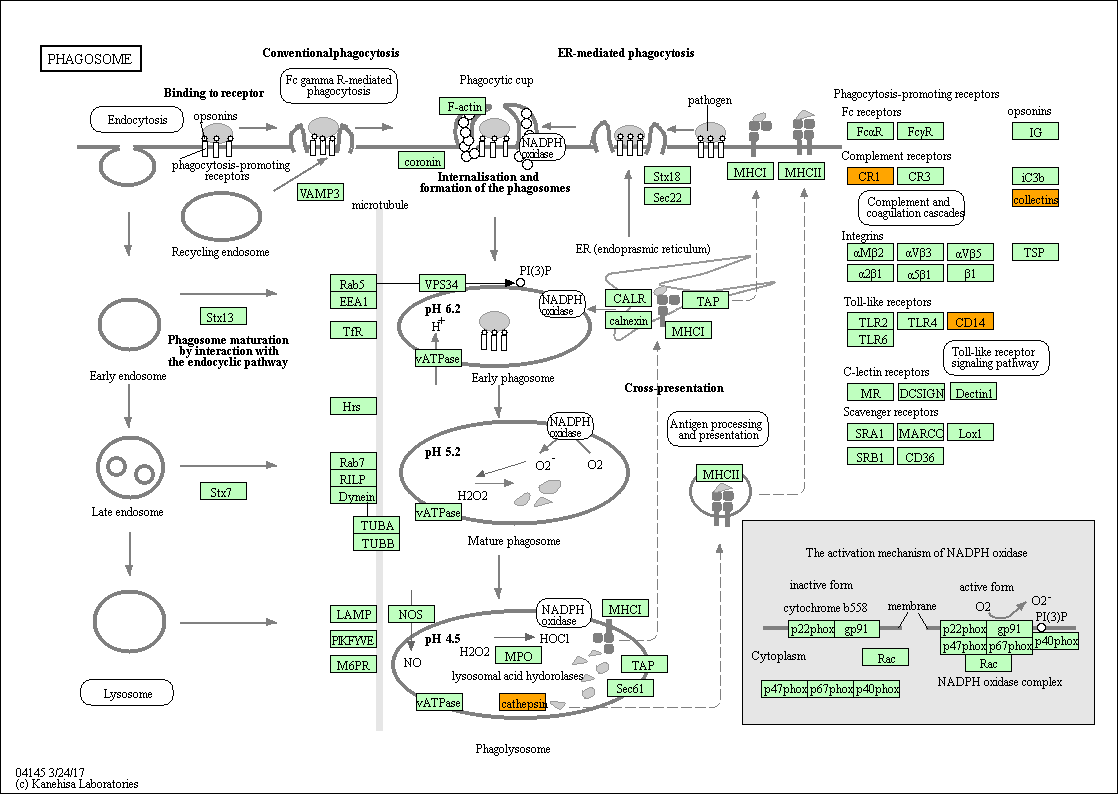

Supplement: Supplementary file 5 [file Data_Sheet_2.zip › Bioinformatics analysis related attachments/KEGG/Sample/png/bta04145.png]

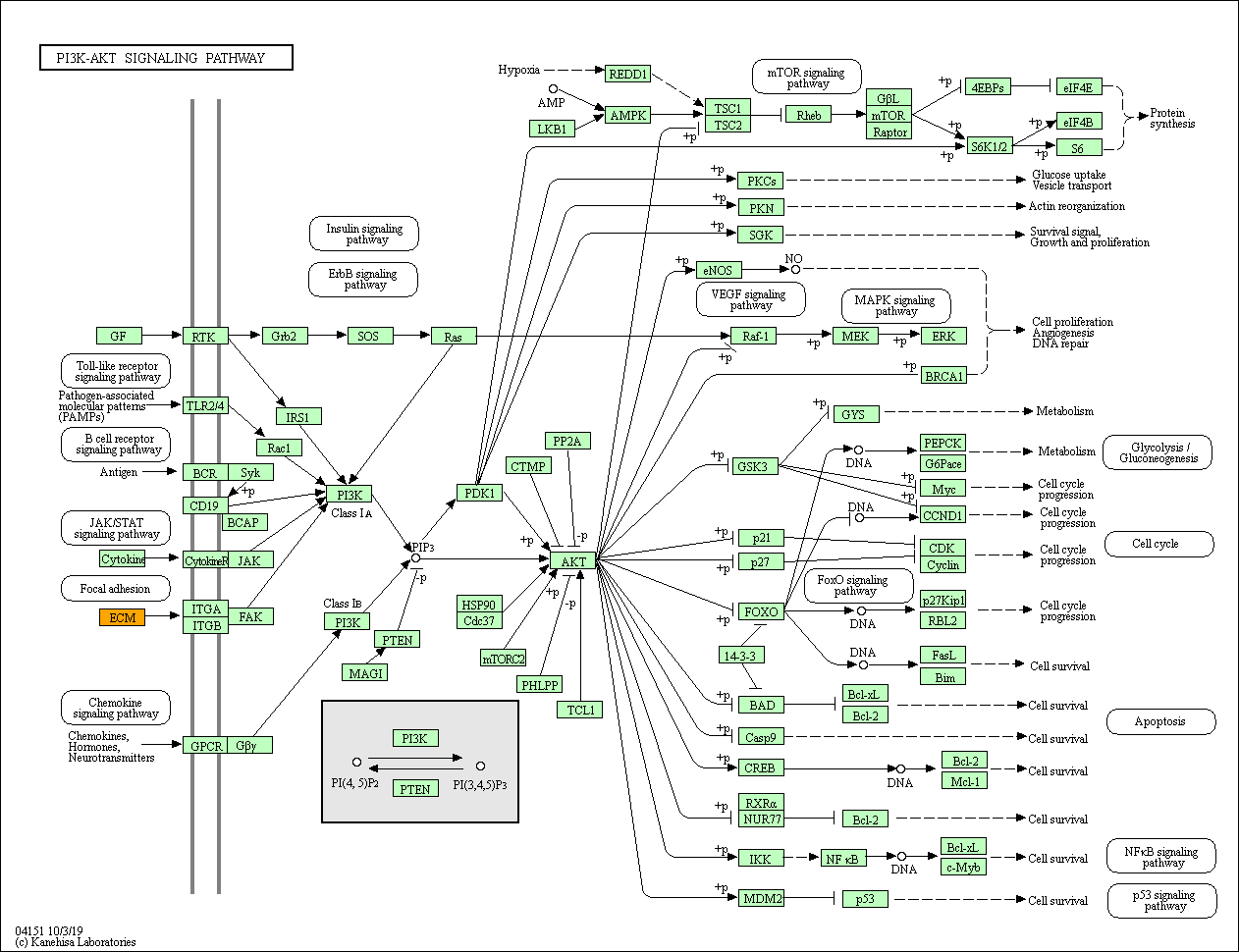

Supplement: Supplementary file 5 [file Data_Sheet_2.zip › Bioinformatics analysis related attachments/KEGG/Sample/png/bta04151.png]

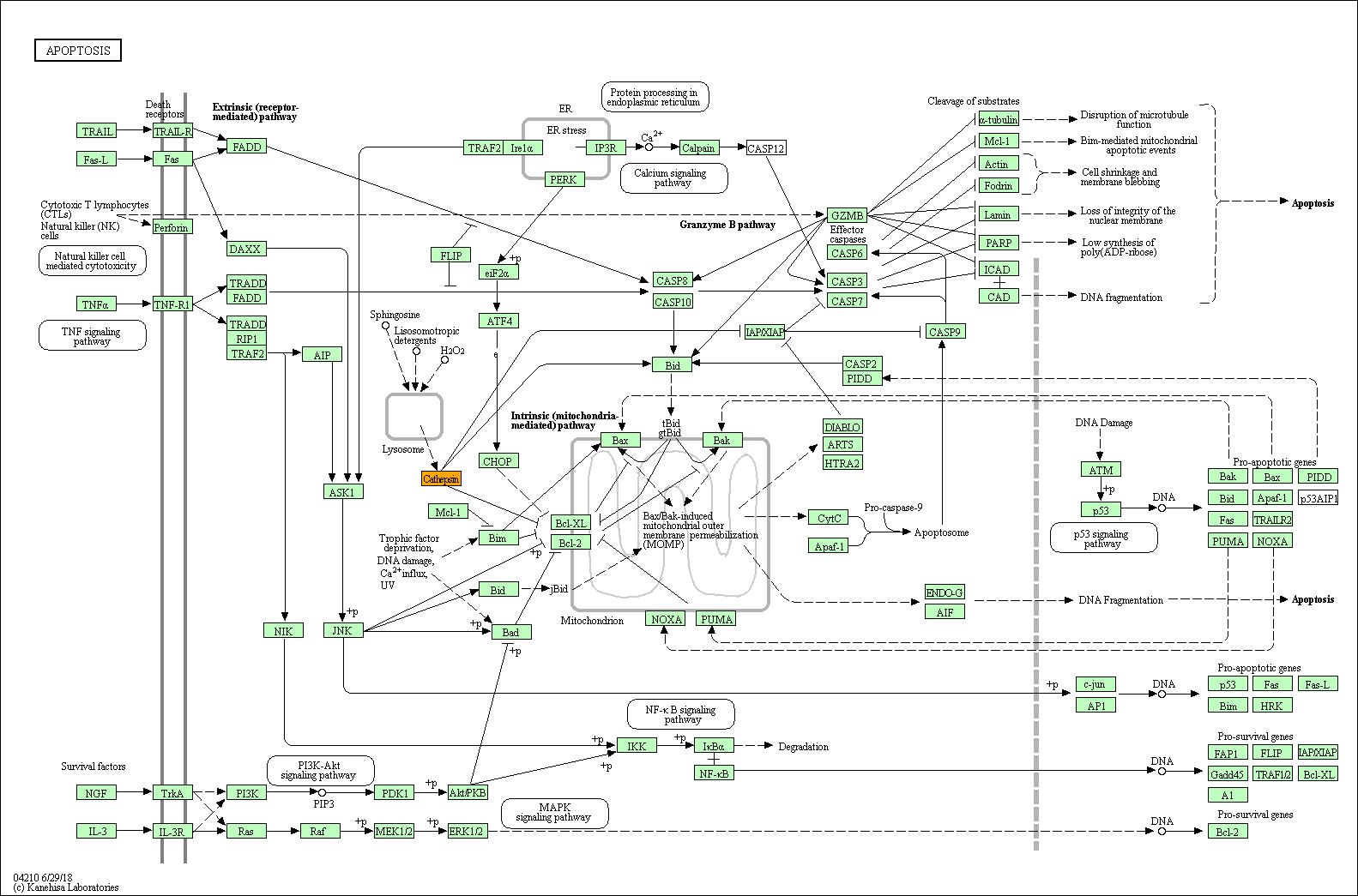

Supplement: Supplementary file 5 [file Data_Sheet_2.zip › Bioinformatics analysis related attachments/KEGG/Sample/png/bta04210.png]

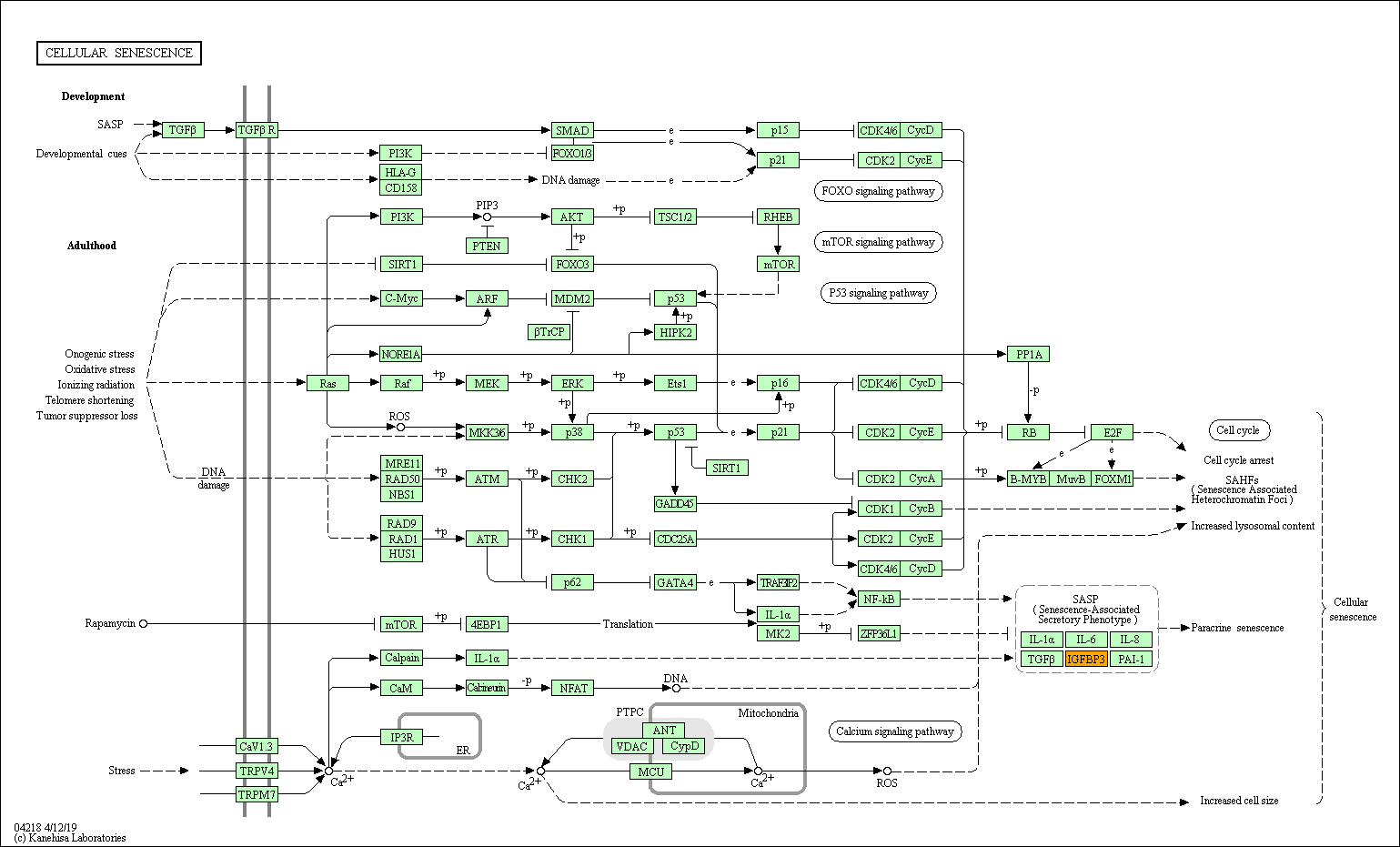

Supplement: Supplementary file 5 [file Data_Sheet_2.zip › Bioinformatics analysis related attachments/KEGG/Sample/png/bta04218.png]

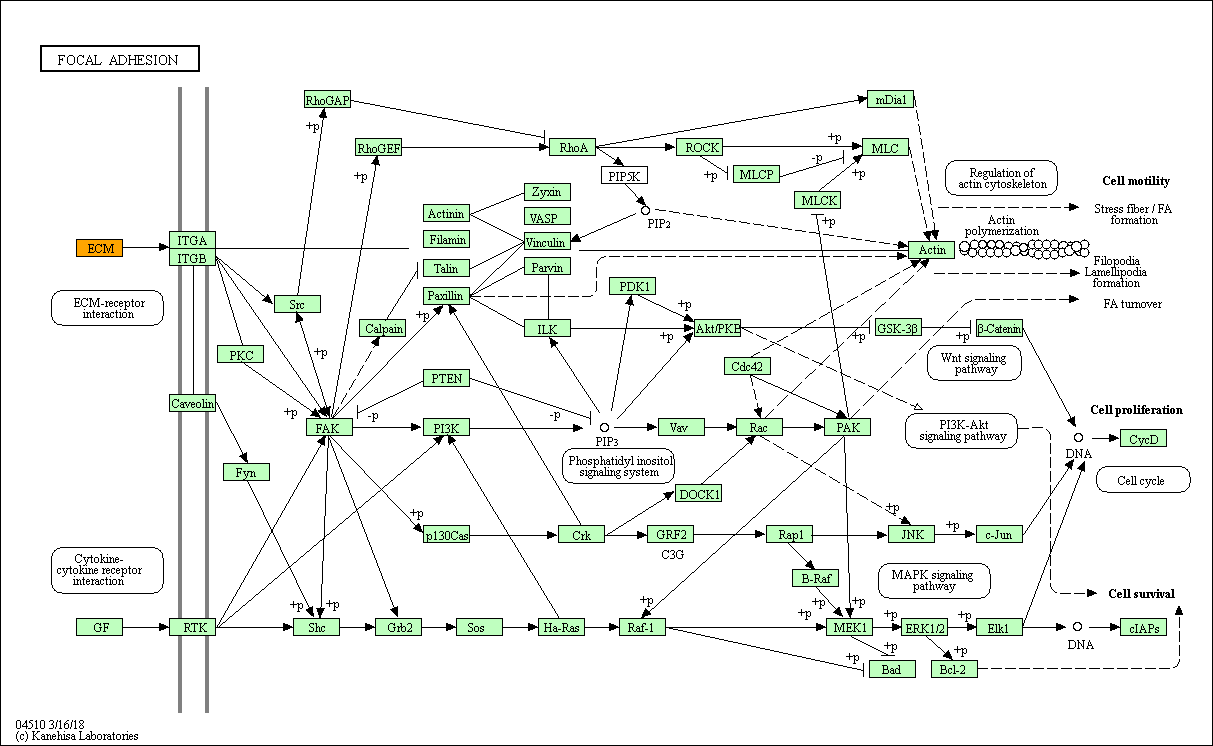

Supplement: Supplementary file 5 [file Data_Sheet_2.zip › Bioinformatics analysis related attachments/KEGG/Sample/png/bta04510.png]

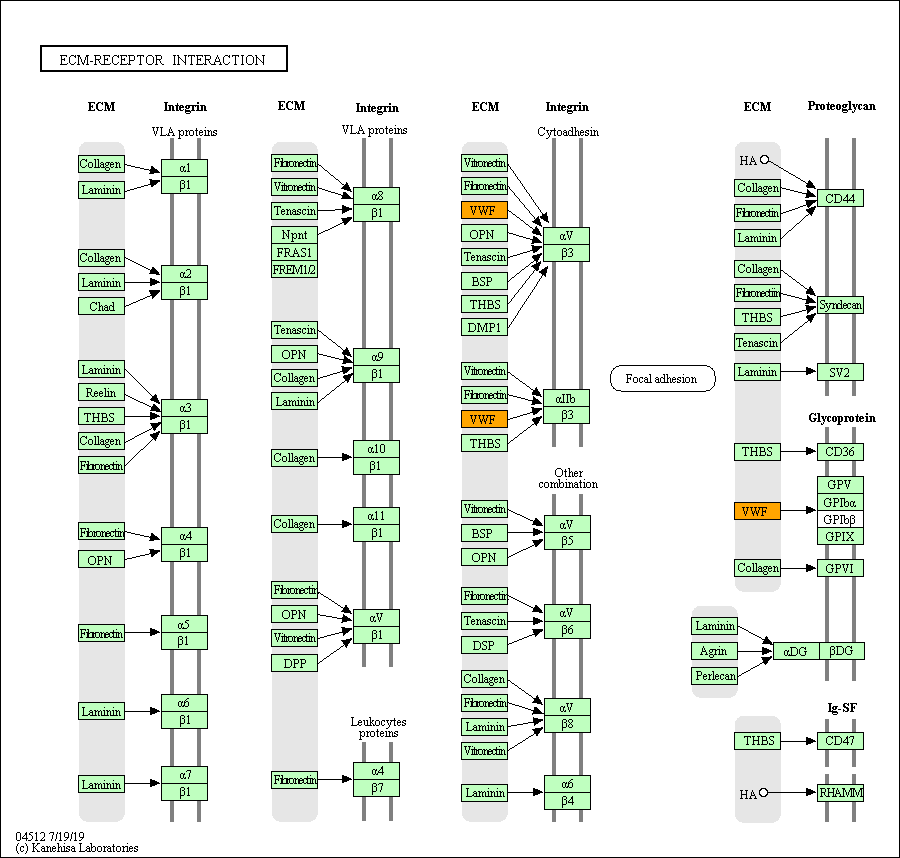

Supplement: Supplementary file 5 [file Data_Sheet_2.zip › Bioinformatics analysis related attachments/KEGG/Sample/png/bta04512.png]

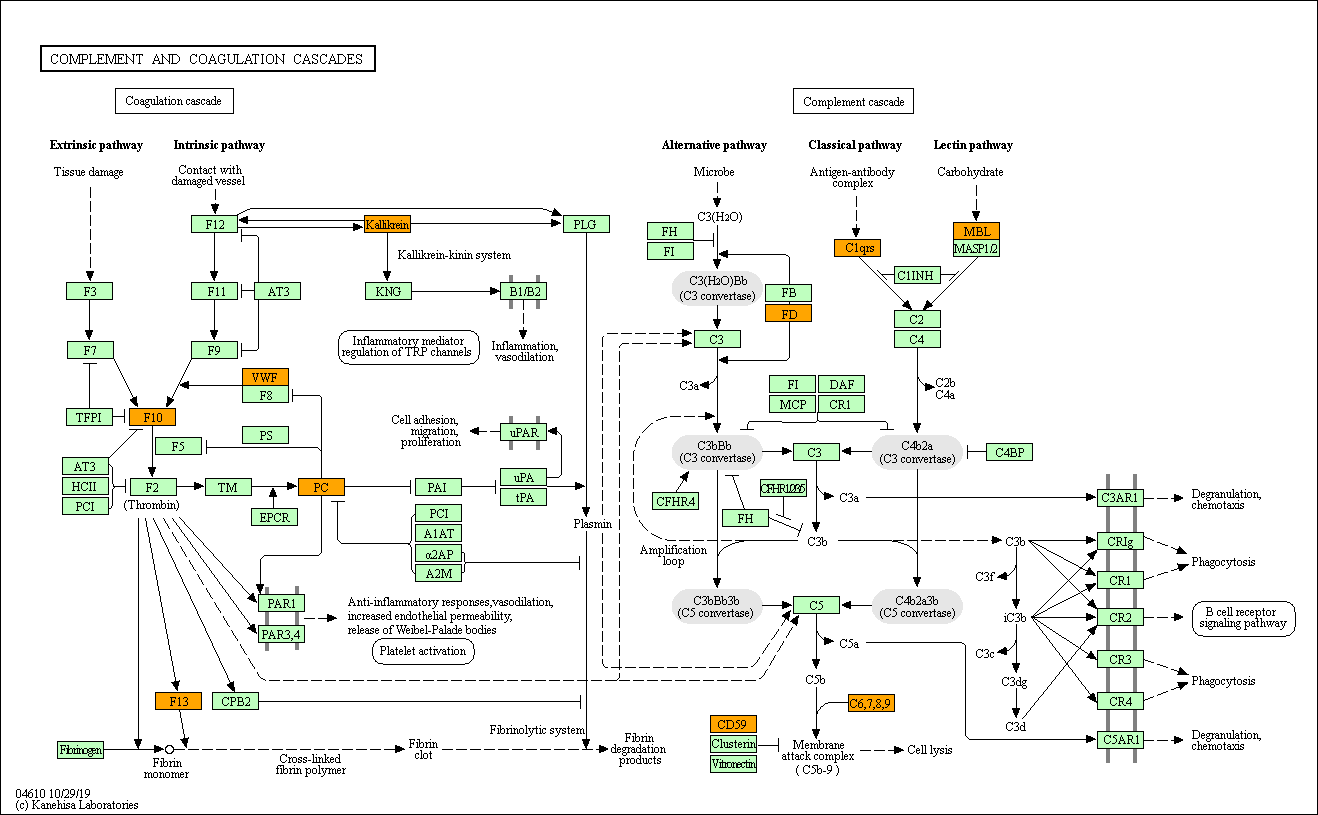

Supplement: Supplementary file 5 [file Data_Sheet_2.zip › Bioinformatics analysis related attachments/KEGG/Sample/png/bta04610.png]

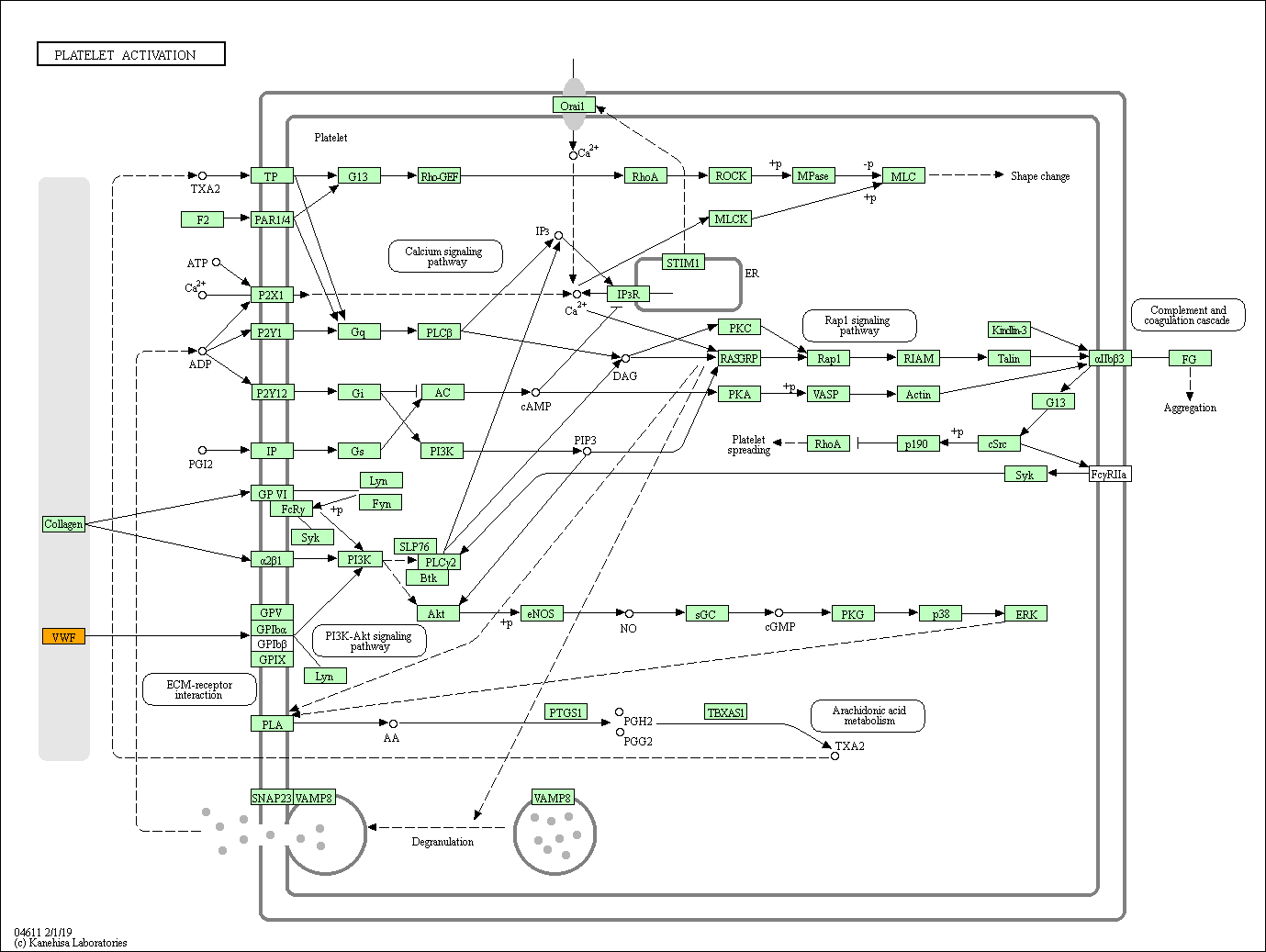

Supplement: Supplementary file 5 [file Data_Sheet_2.zip › Bioinformatics analysis related attachments/KEGG/Sample/png/bta04611.png]

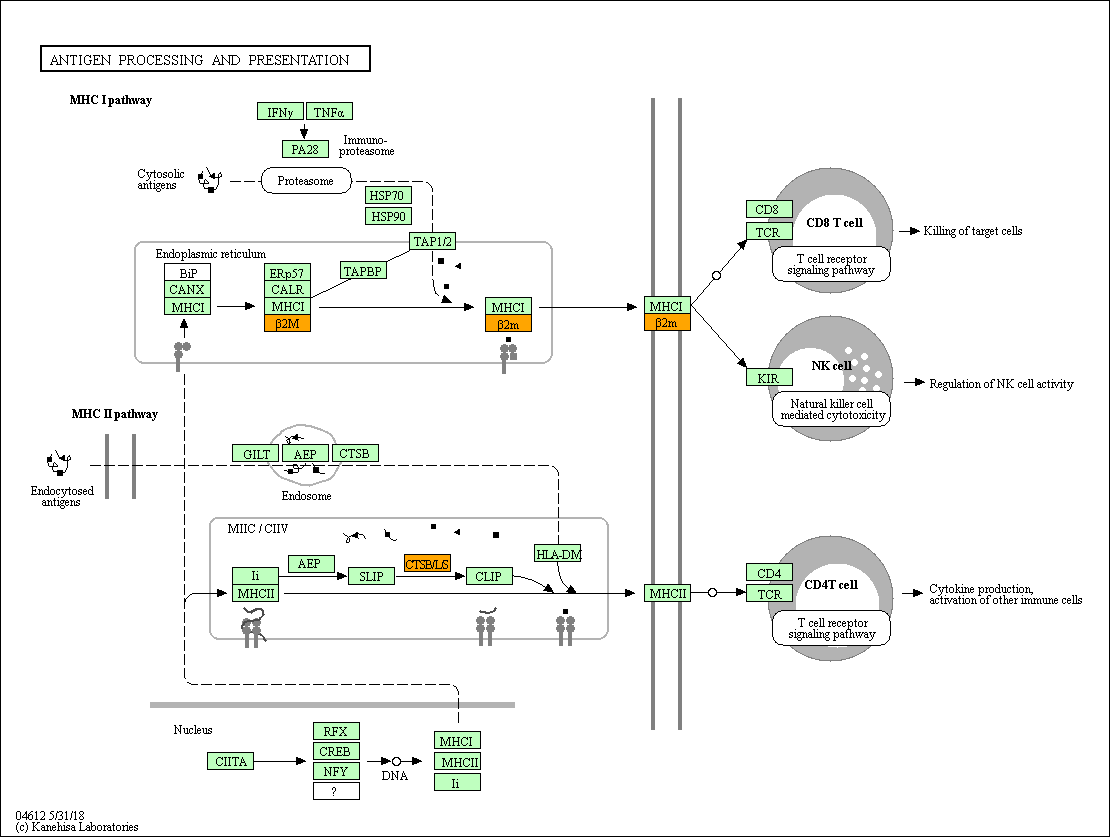

Supplement: Supplementary file 5 [file Data_Sheet_2.zip › Bioinformatics analysis related attachments/KEGG/Sample/png/bta04612.png]

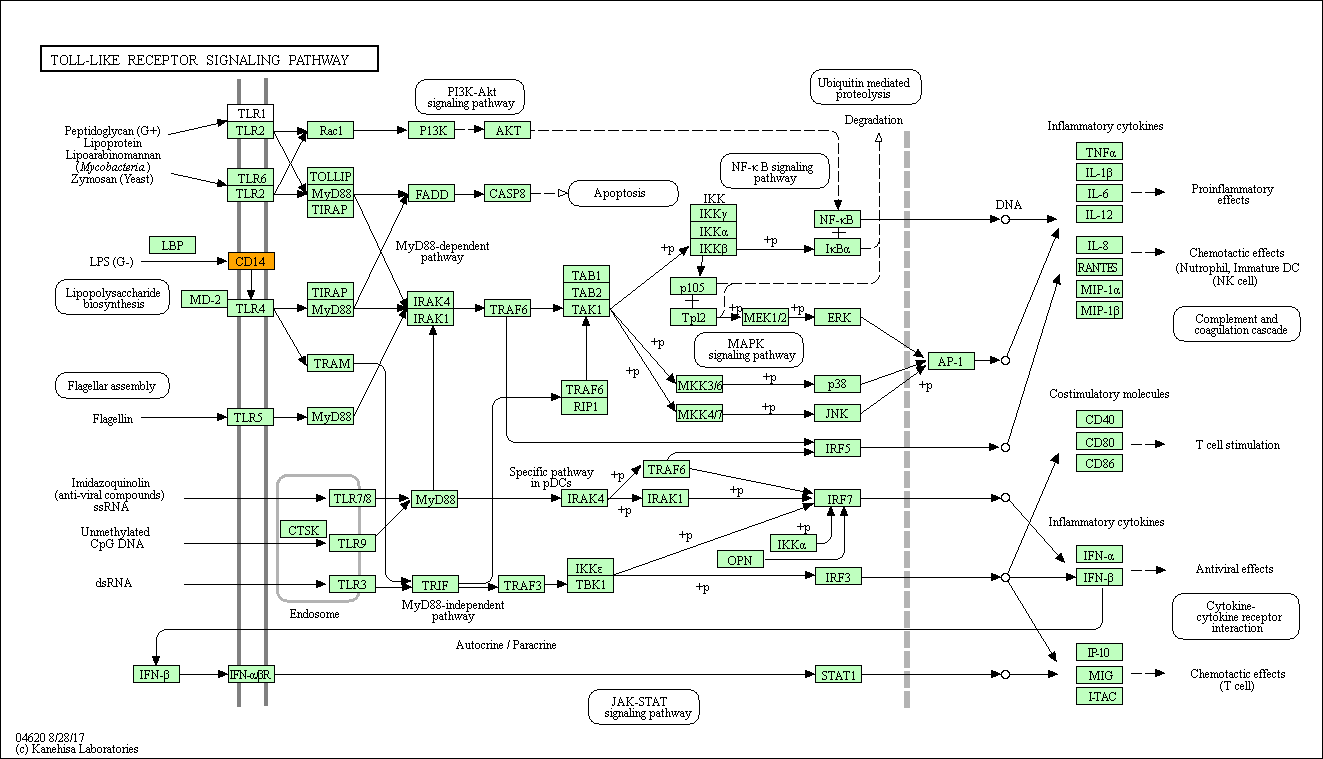

Supplement: Supplementary file 5 [file Data_Sheet_2.zip › Bioinformatics analysis related attachments/KEGG/Sample/png/bta04620.png]

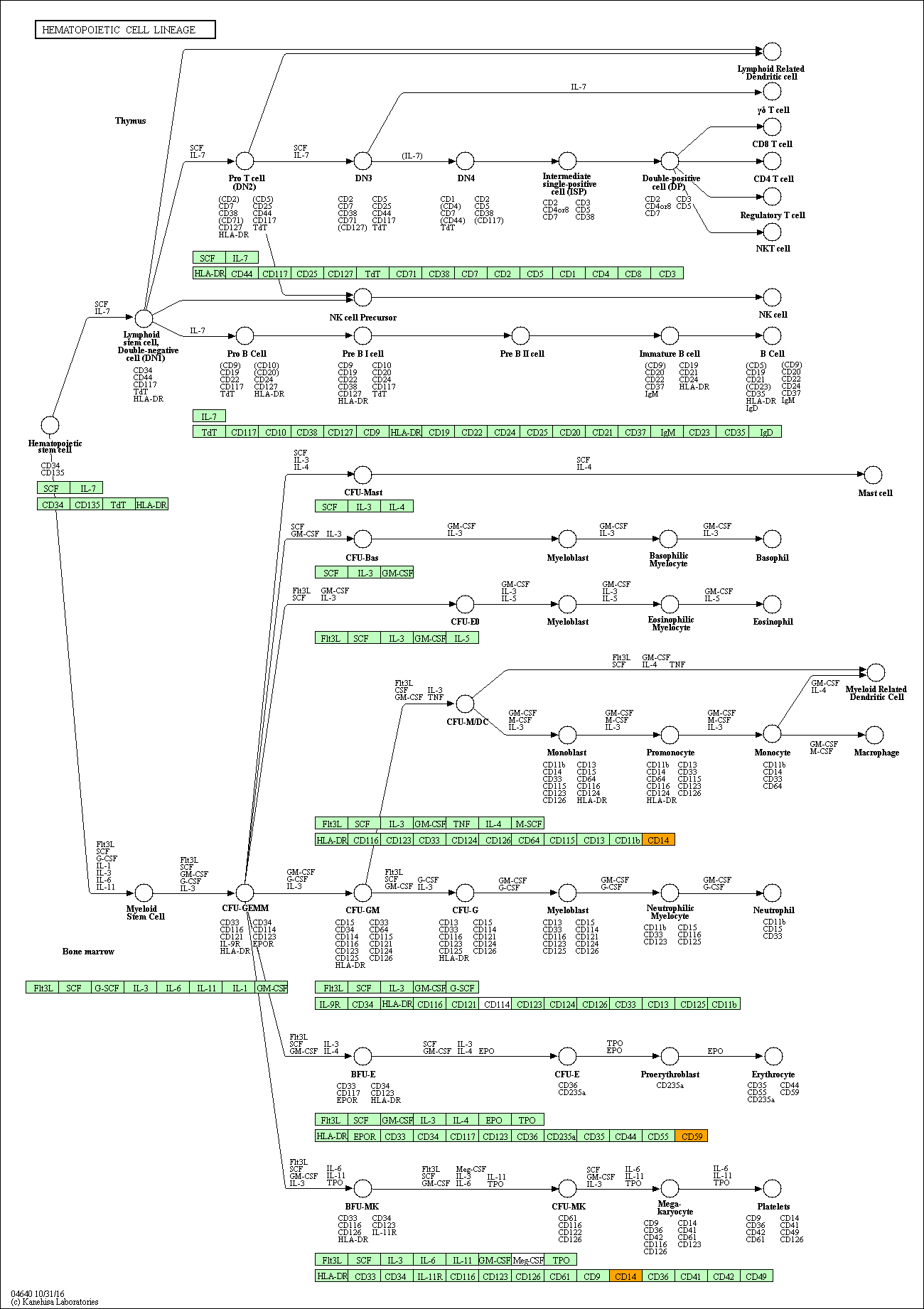

Supplement: Supplementary file 5 [file Data_Sheet_2.zip › Bioinformatics analysis related attachments/KEGG/Sample/png/bta04640.png]

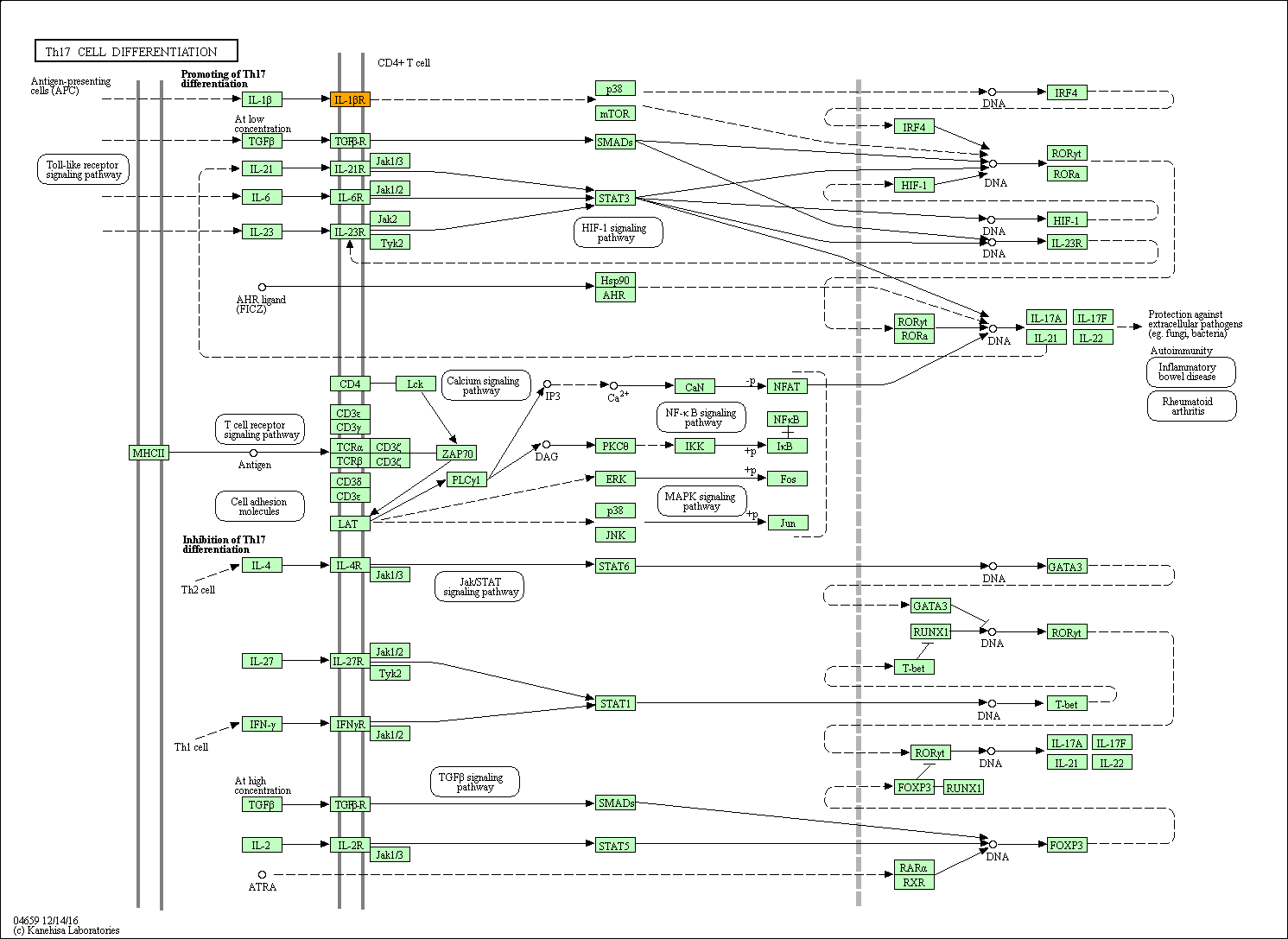

Supplement: Supplementary file 5 [file Data_Sheet_2.zip › Bioinformatics analysis related attachments/KEGG/Sample/png/bta04659.png]

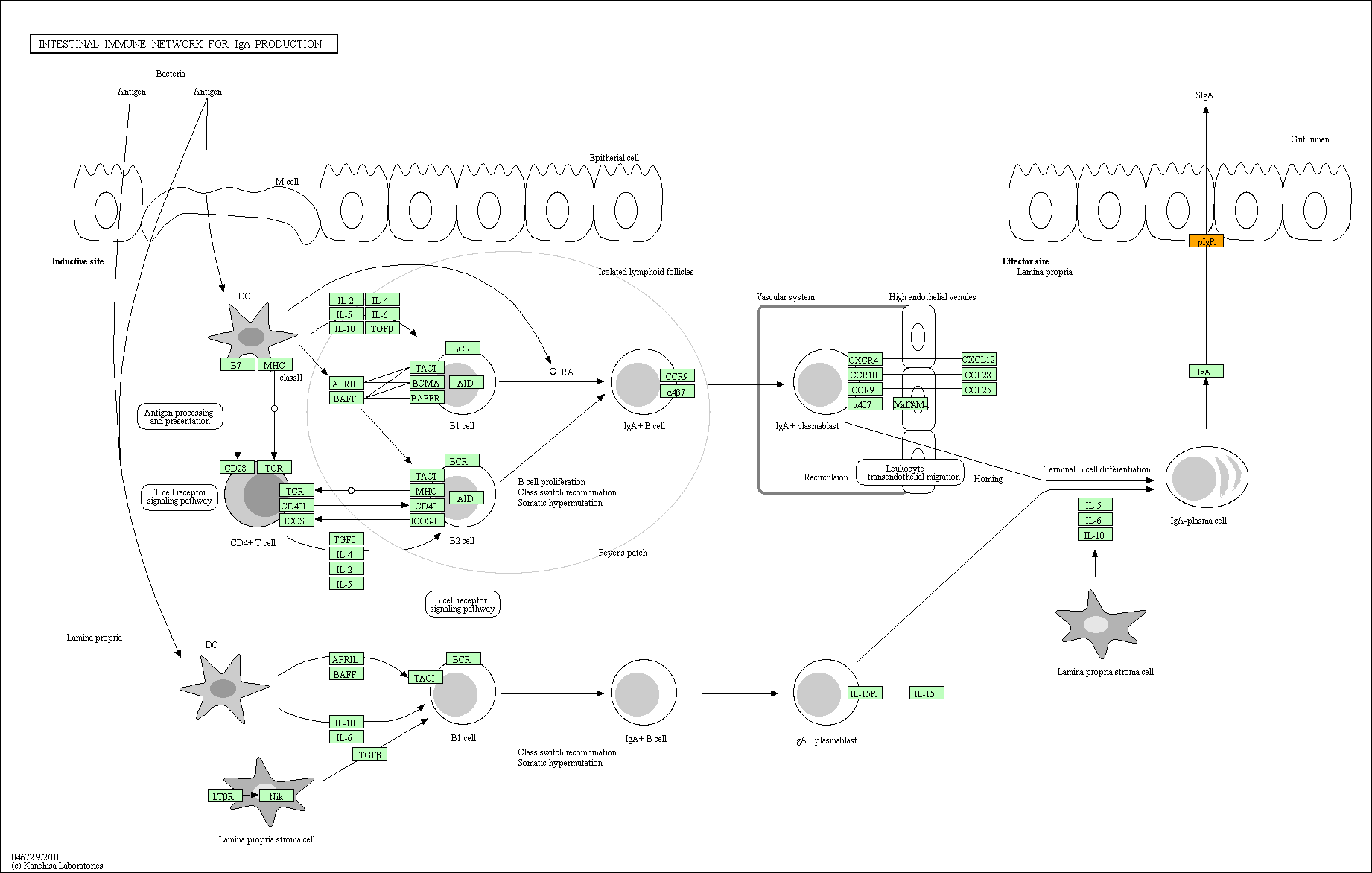

Supplement: Supplementary file 5 [file Data_Sheet_2.zip › Bioinformatics analysis related attachments/KEGG/Sample/png/bta04672.png]

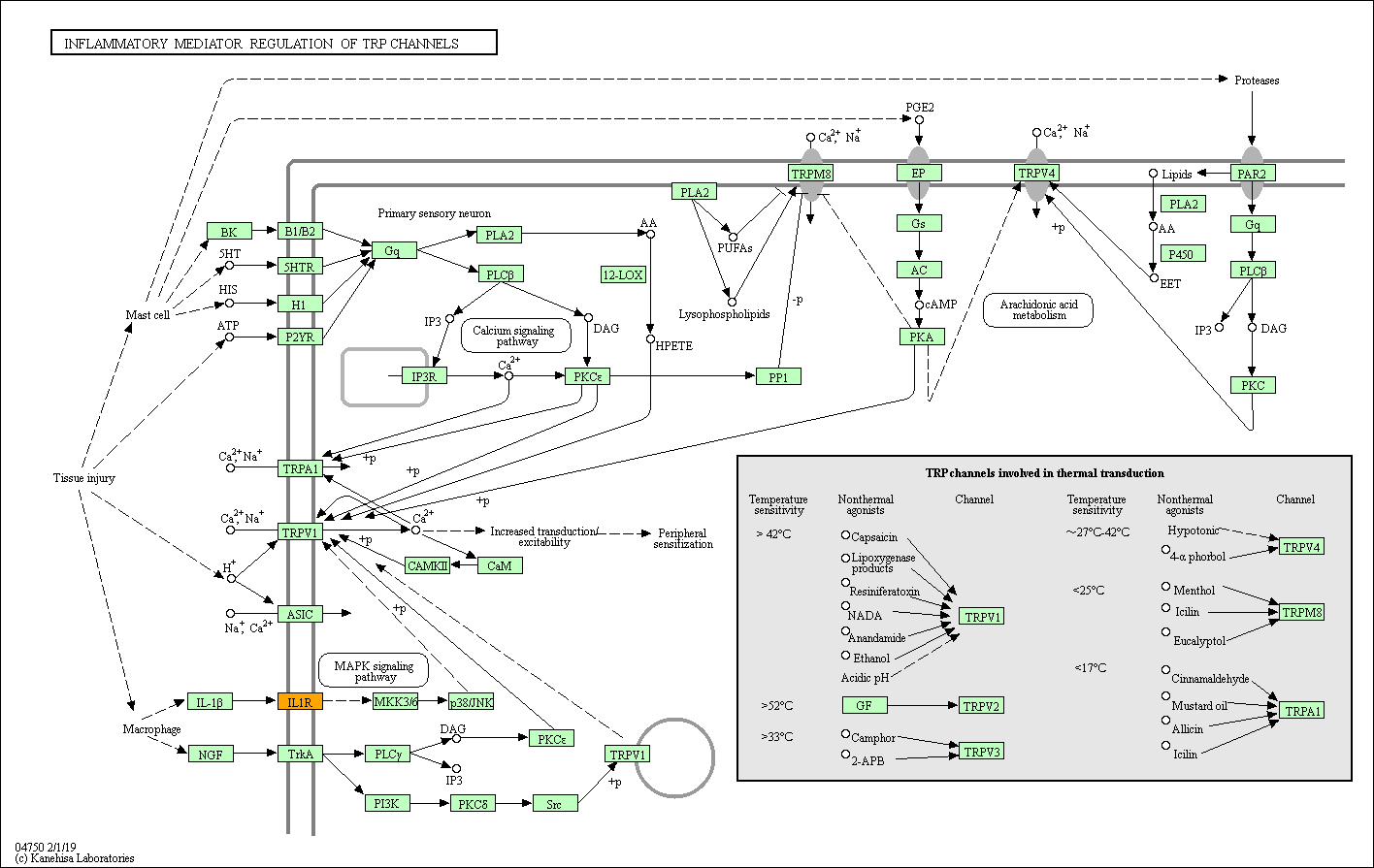

Supplement: Supplementary file 5 [file Data_Sheet_2.zip › Bioinformatics analysis related attachments/KEGG/Sample/png/bta04750.png]

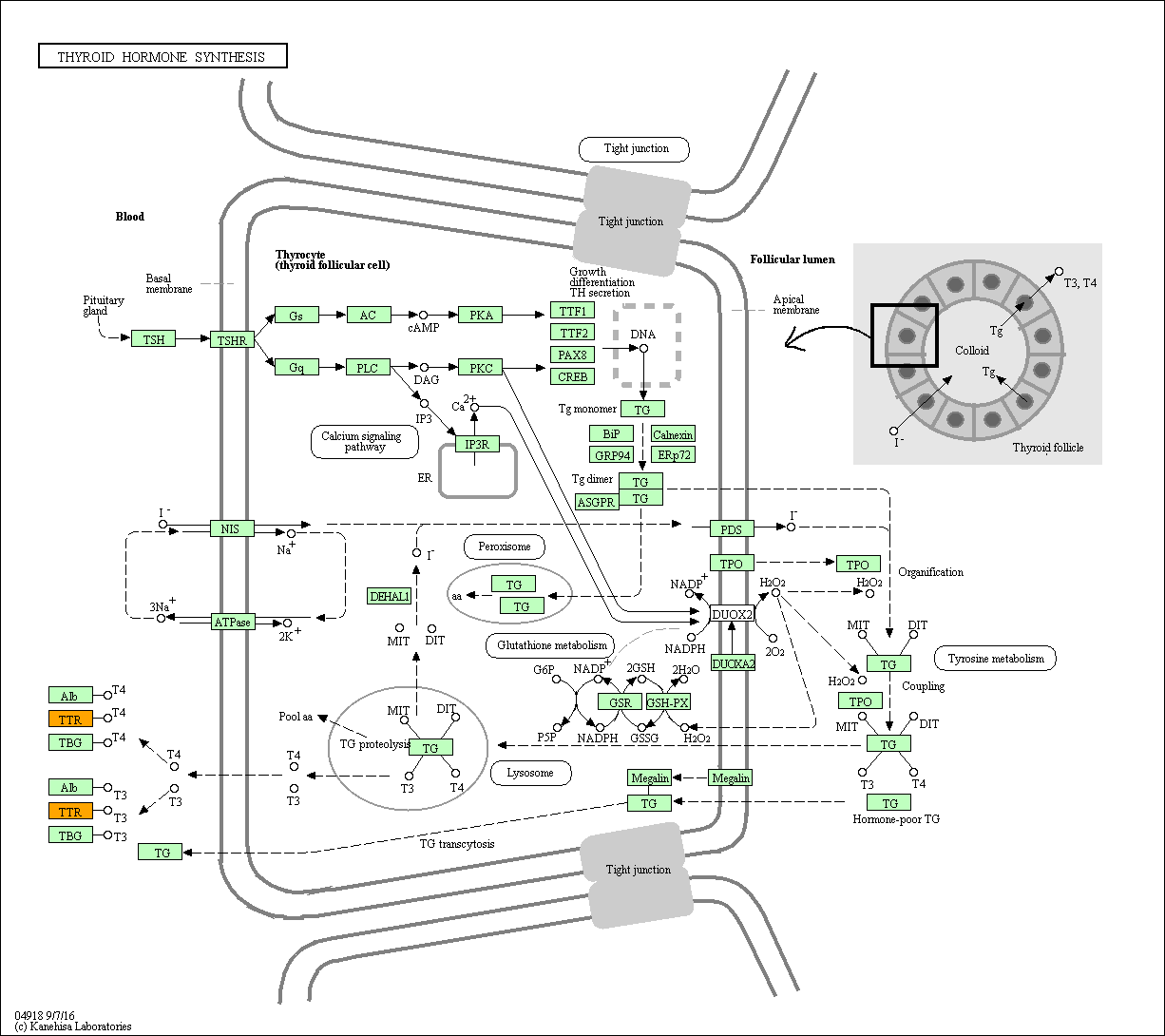

Supplement: Supplementary file 5 [file Data_Sheet_2.zip › Bioinformatics analysis related attachments/KEGG/Sample/png/bta04918.png]

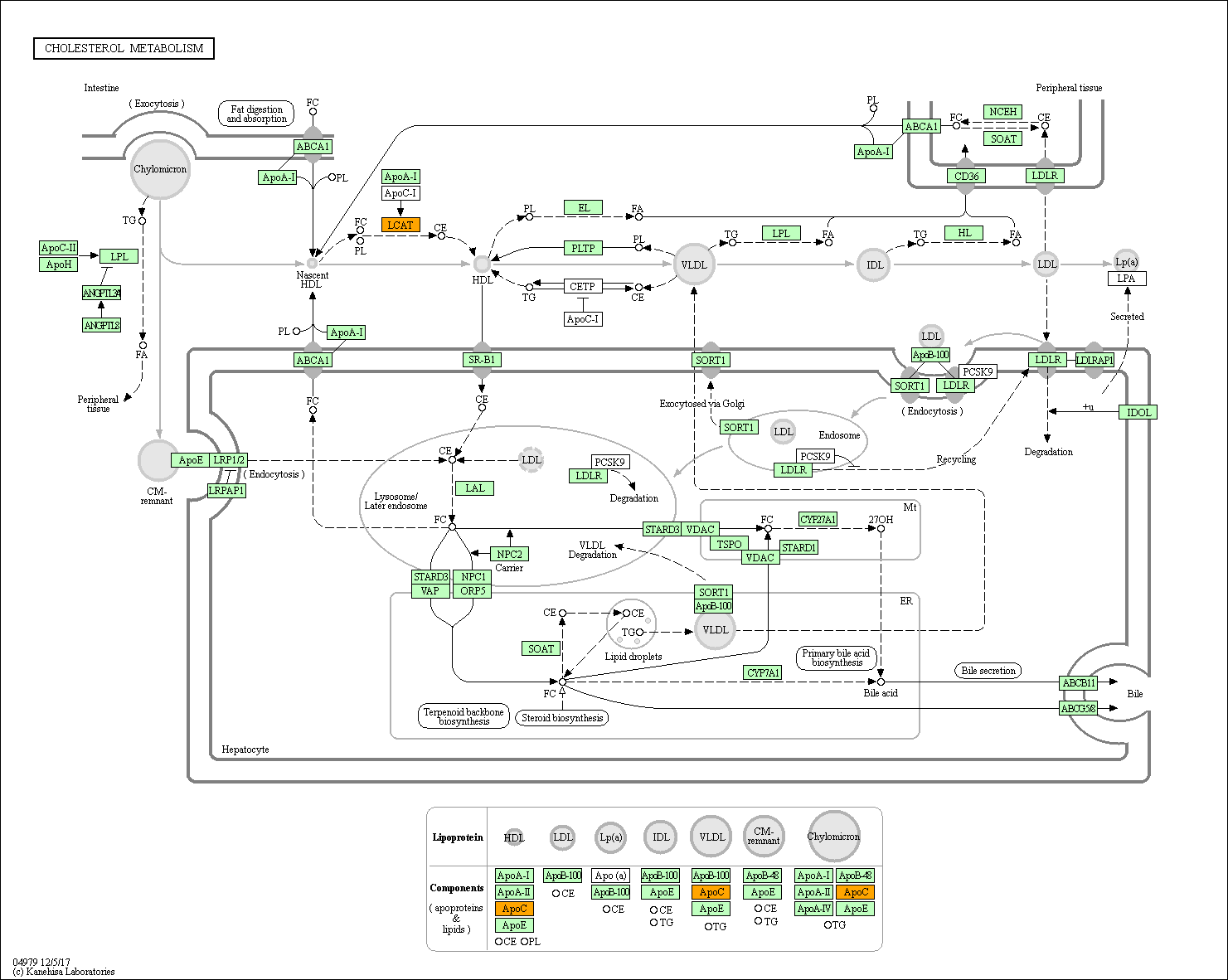

Supplement: Supplementary file 5 [file Data_Sheet_2.zip › Bioinformatics analysis related attachments/KEGG/Sample/png/bta04979.png]

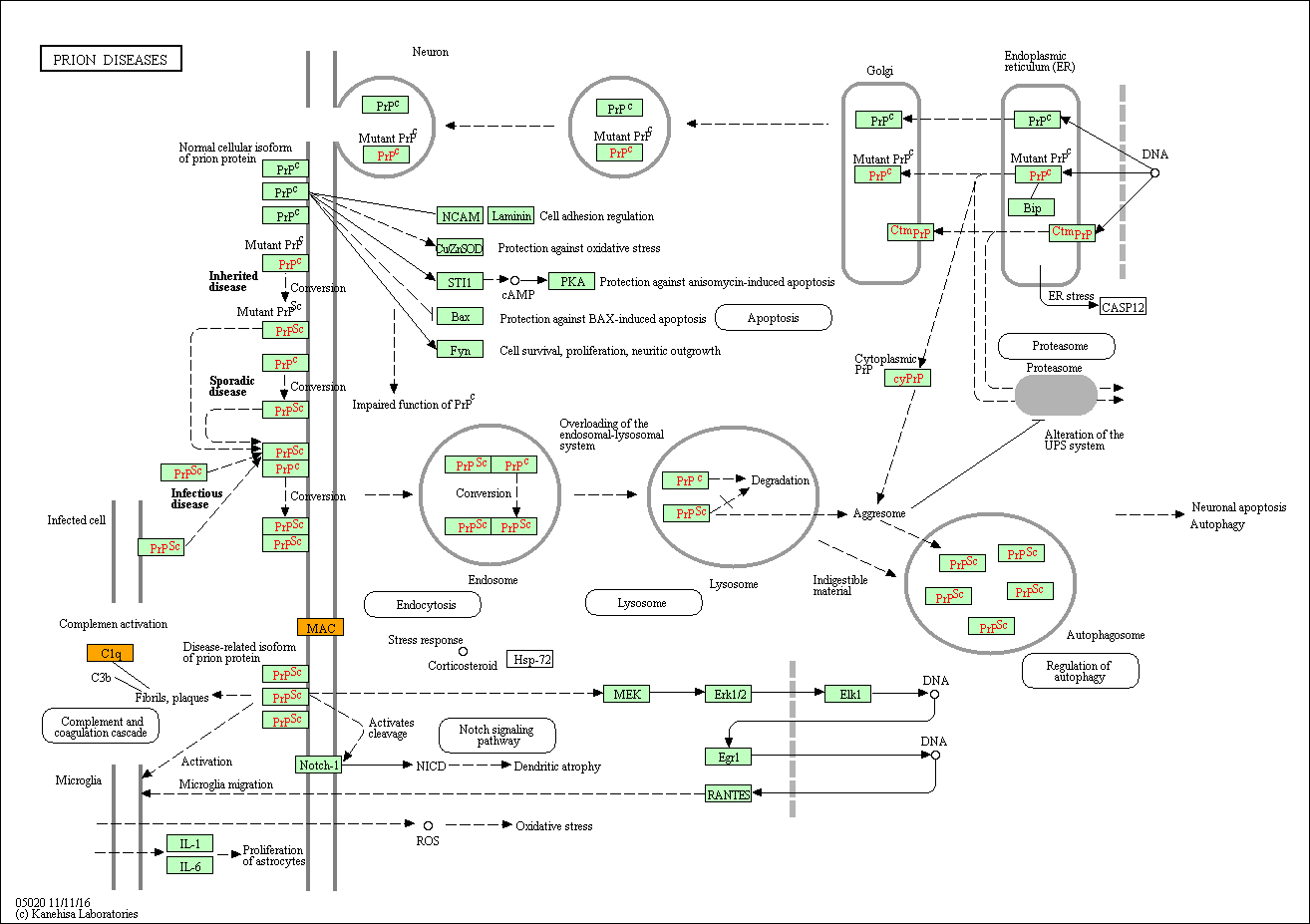

Supplement: Supplementary file 5 [file Data_Sheet_2.zip › Bioinformatics analysis related attachments/KEGG/Sample/png/bta05020.png]

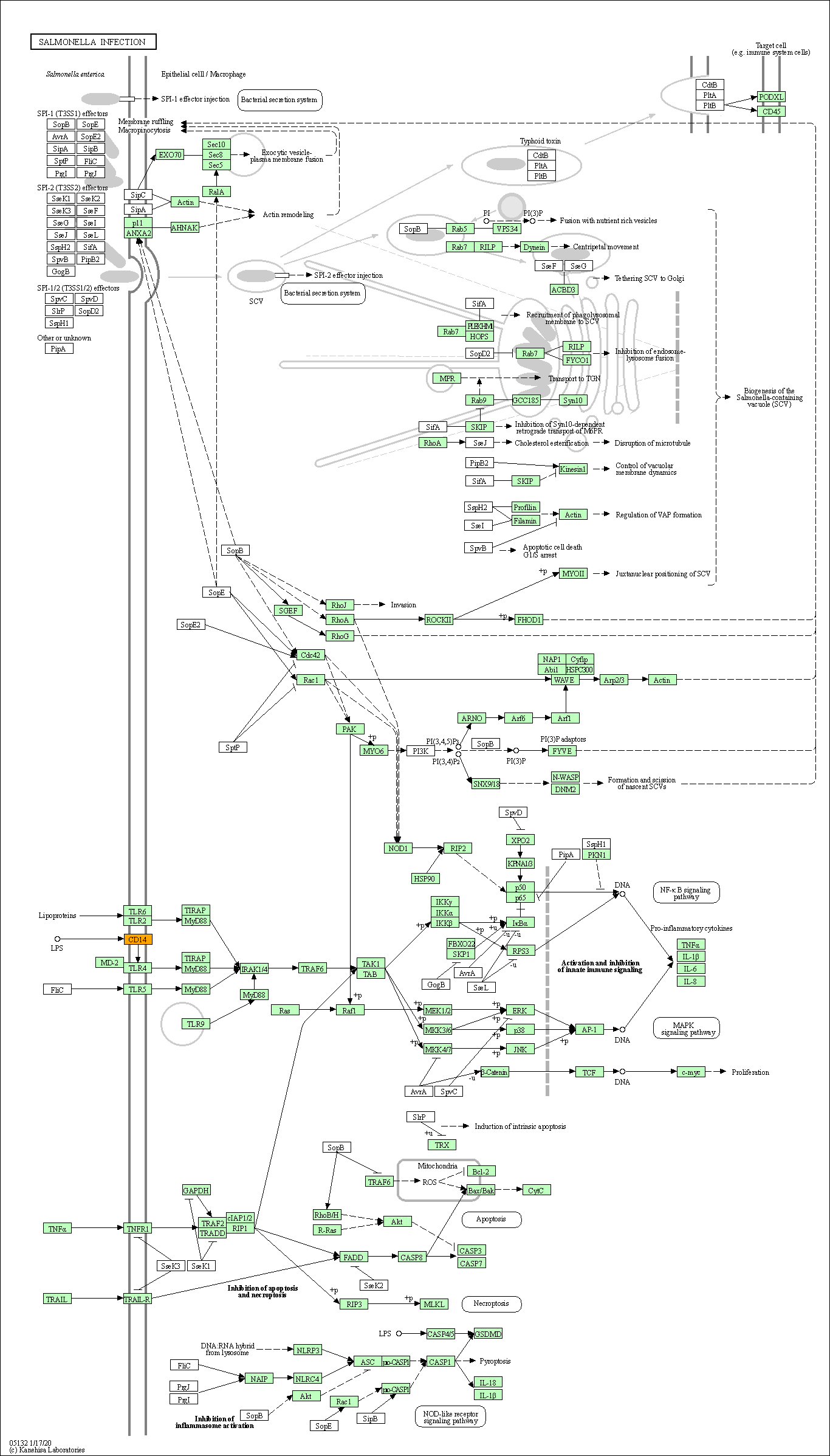

Supplement: Supplementary file 5 [file Data_Sheet_2.zip › Bioinformatics analysis related attachments/KEGG/Sample/png/bta05132.png]

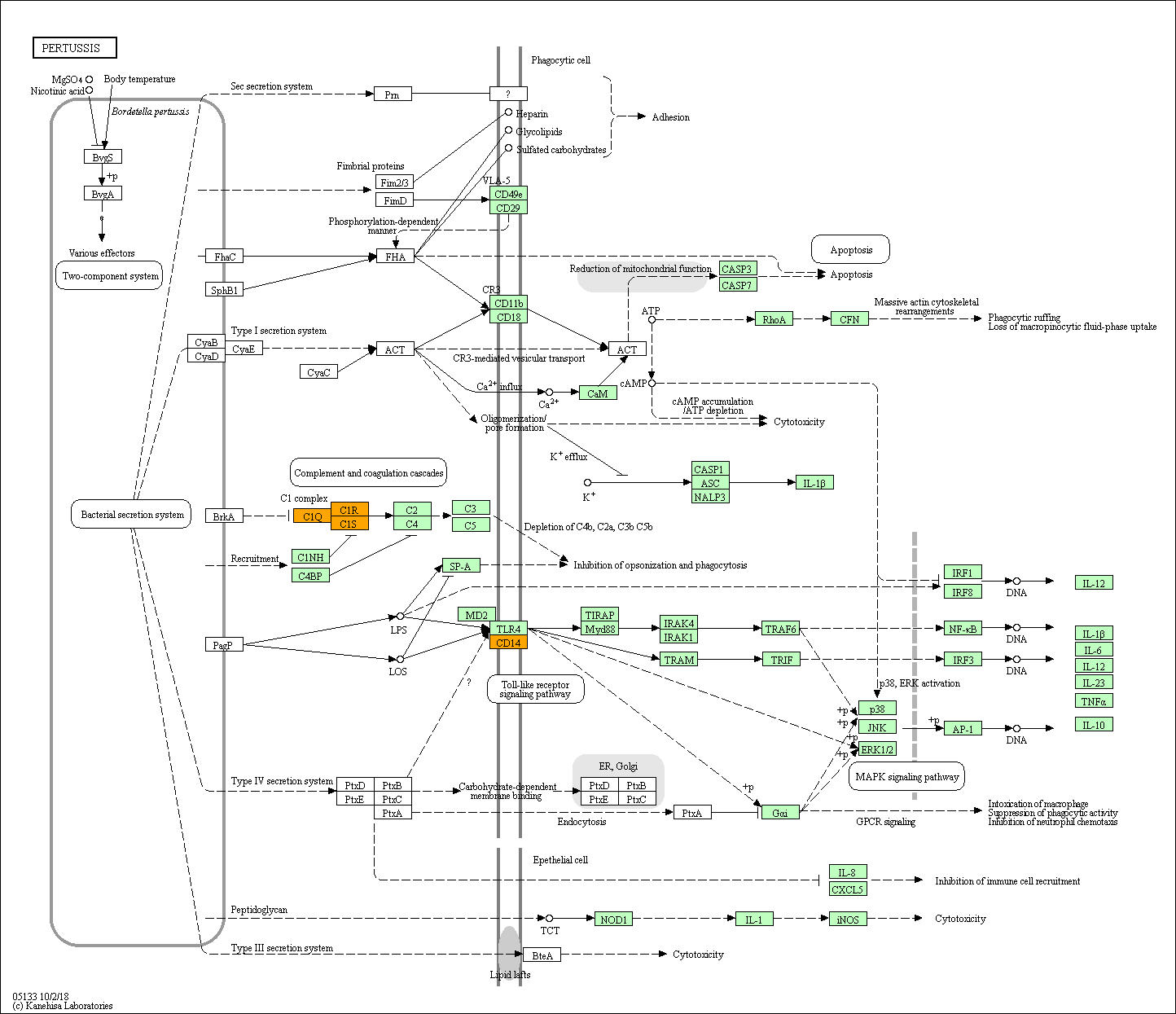

Supplement: Supplementary file 5 [file Data_Sheet_2.zip › Bioinformatics analysis related attachments/KEGG/Sample/png/bta05133.png]

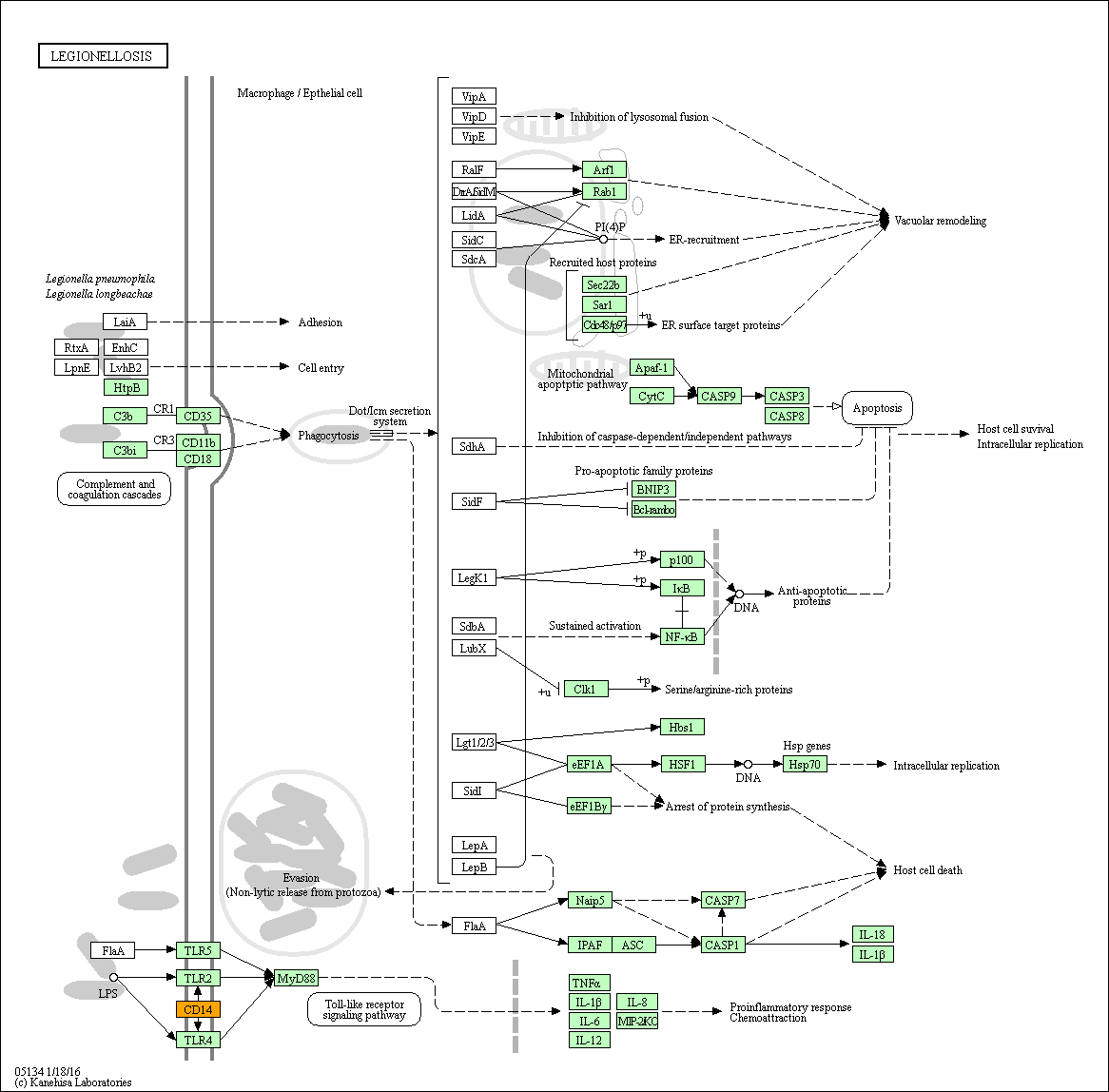

Supplement: Supplementary file 5 [file Data_Sheet_2.zip › Bioinformatics analysis related attachments/KEGG/Sample/png/bta05134.png]

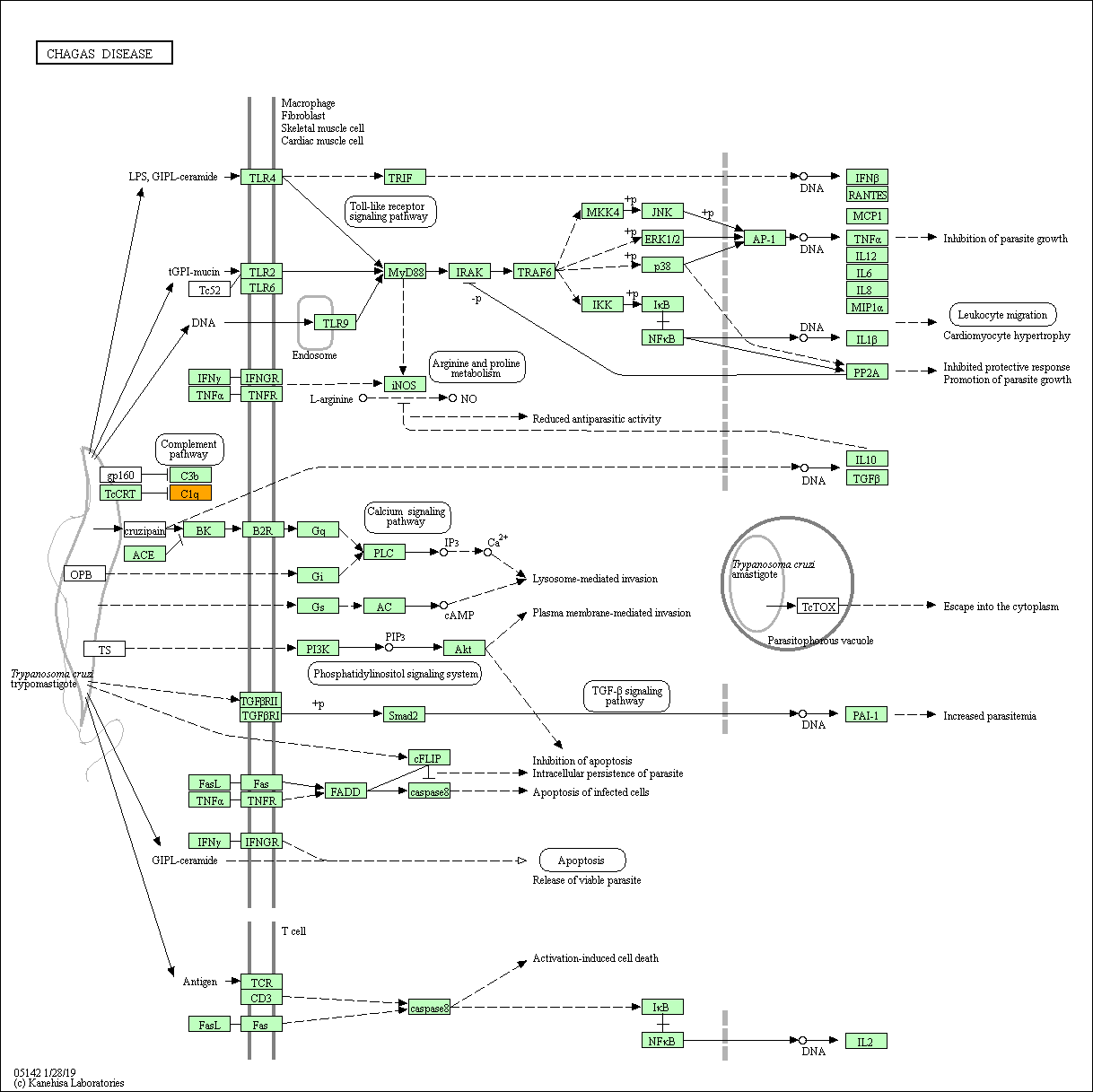

Supplement: Supplementary file 5 [file Data_Sheet_2.zip › Bioinformatics analysis related attachments/KEGG/Sample/png/bta05142.png]

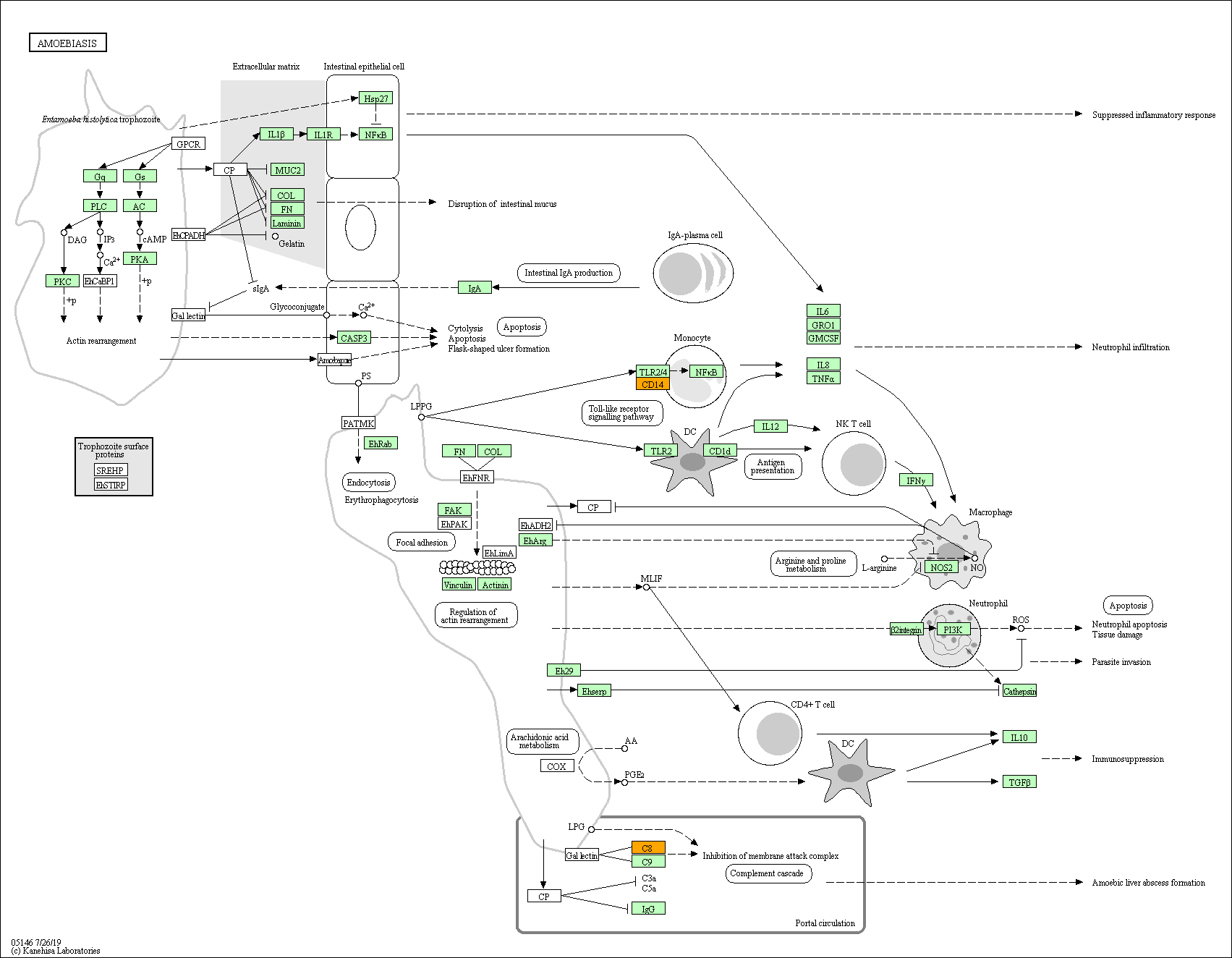

Supplement: Supplementary file 5 [file Data_Sheet_2.zip › Bioinformatics analysis related attachments/KEGG/Sample/png/bta05146.png]

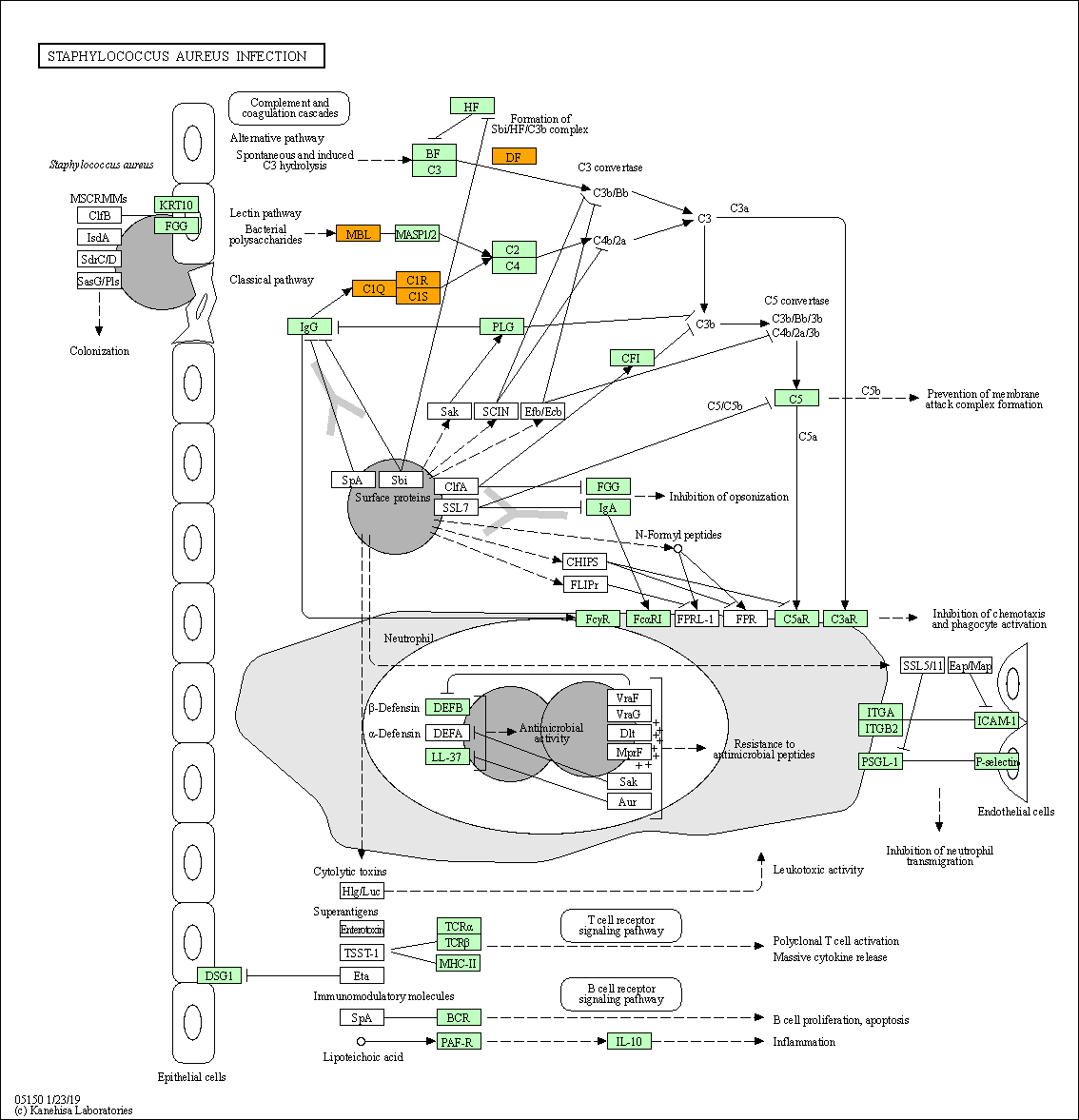

Supplement: Supplementary file 5 [file Data_Sheet_2.zip › Bioinformatics analysis related attachments/KEGG/Sample/png/bta05150.png]

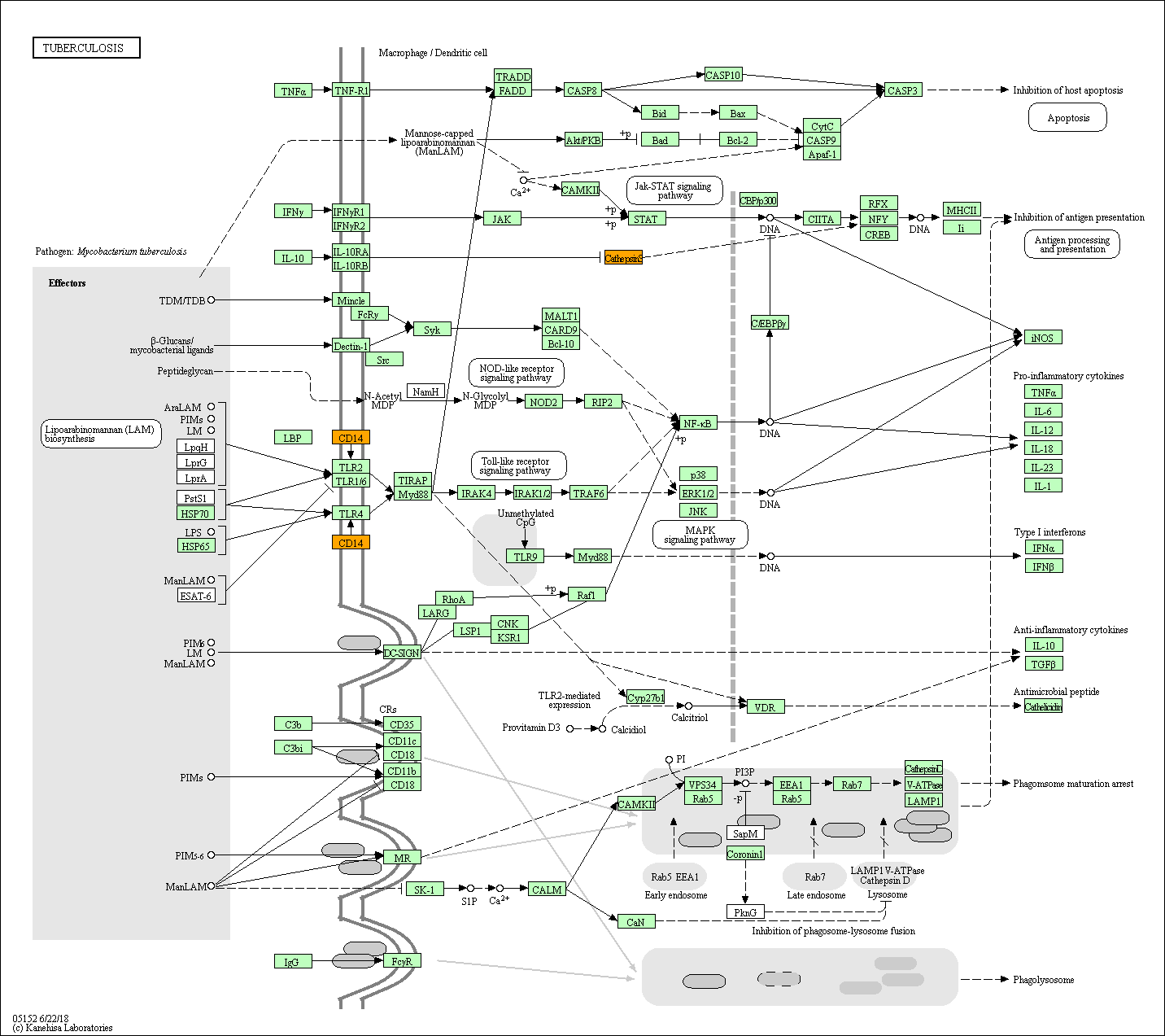

Supplement: Supplementary file 5 [file Data_Sheet_2.zip › Bioinformatics analysis related attachments/KEGG/Sample/png/bta05152.png]

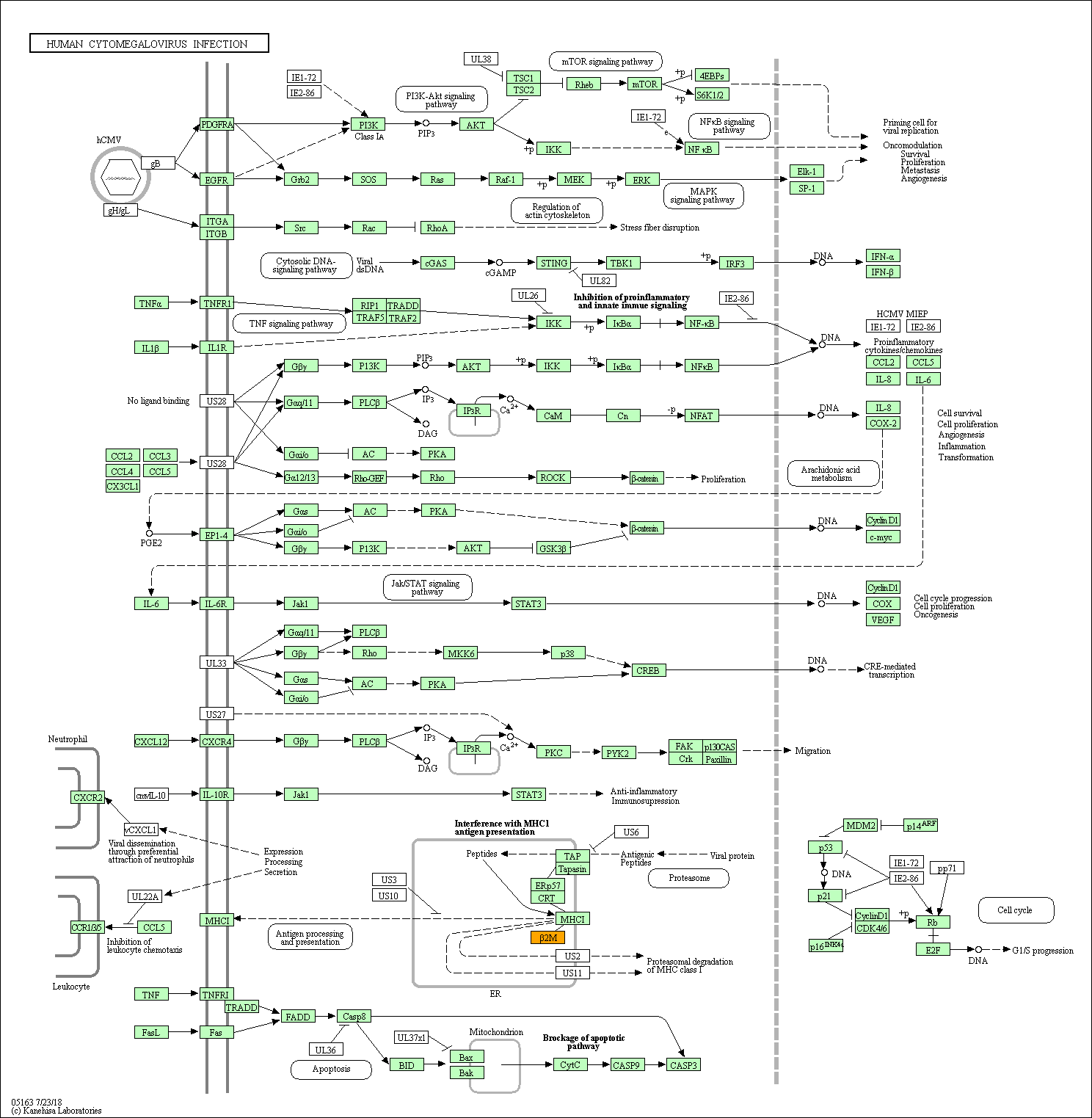

Supplement: Supplementary file 5 [file Data_Sheet_2.zip › Bioinformatics analysis related attachments/KEGG/Sample/png/bta05163.png]

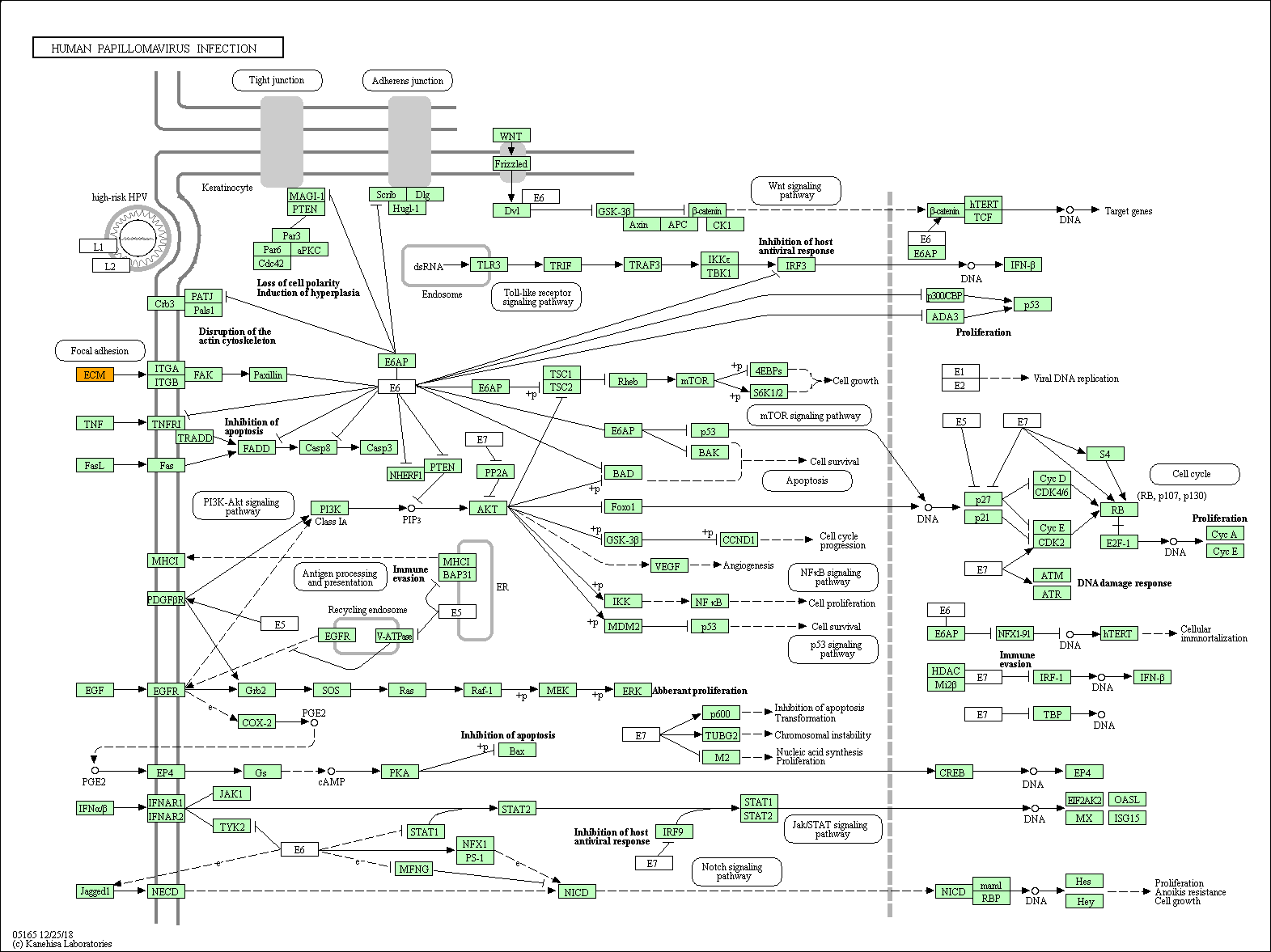

Supplement: Supplementary file 5 [file Data_Sheet_2.zip › Bioinformatics analysis related attachments/KEGG/Sample/png/bta05165.png]

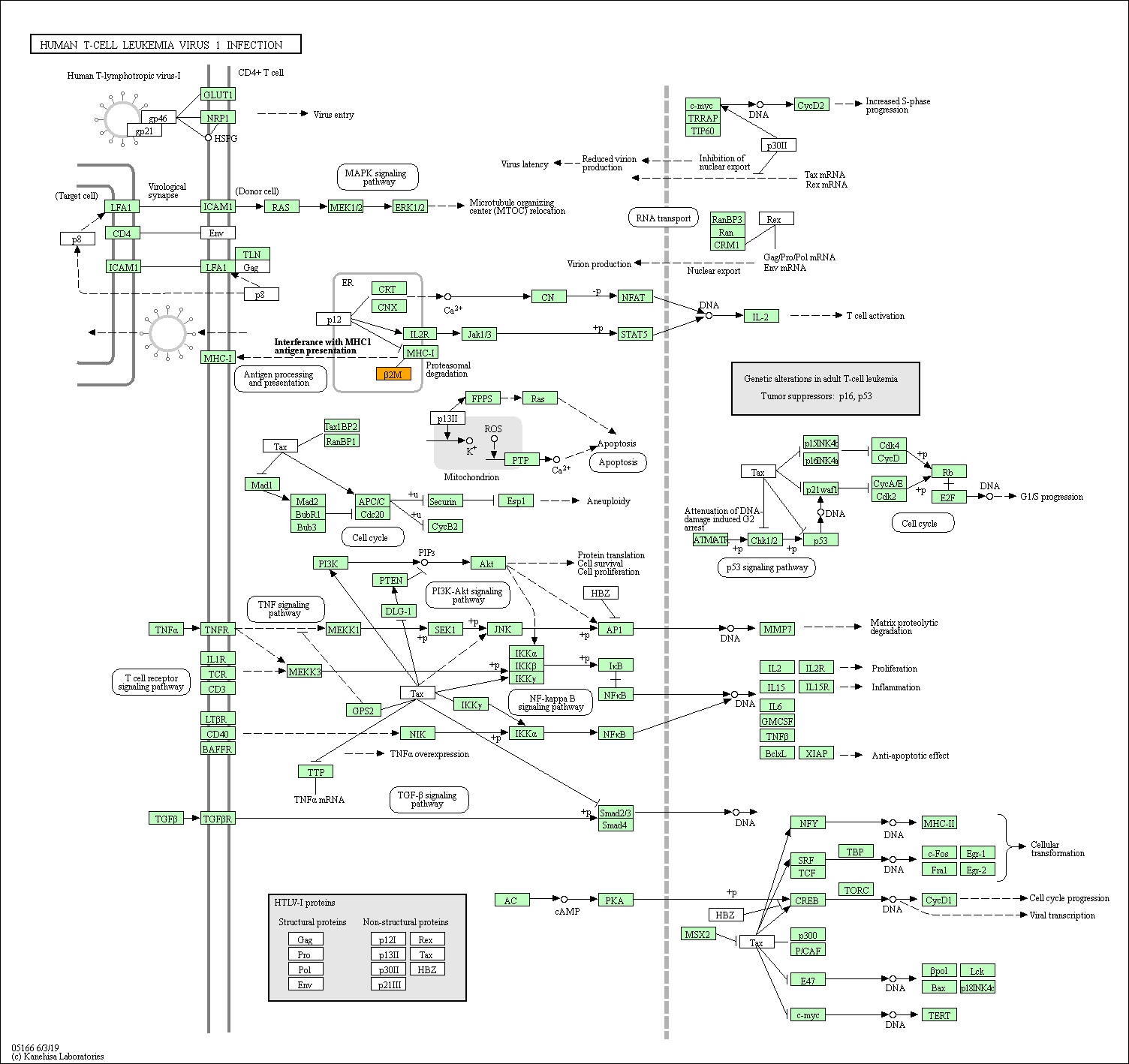

Supplement: Supplementary file 5 [file Data_Sheet_2.zip › Bioinformatics analysis related attachments/KEGG/Sample/png/bta05166.png]

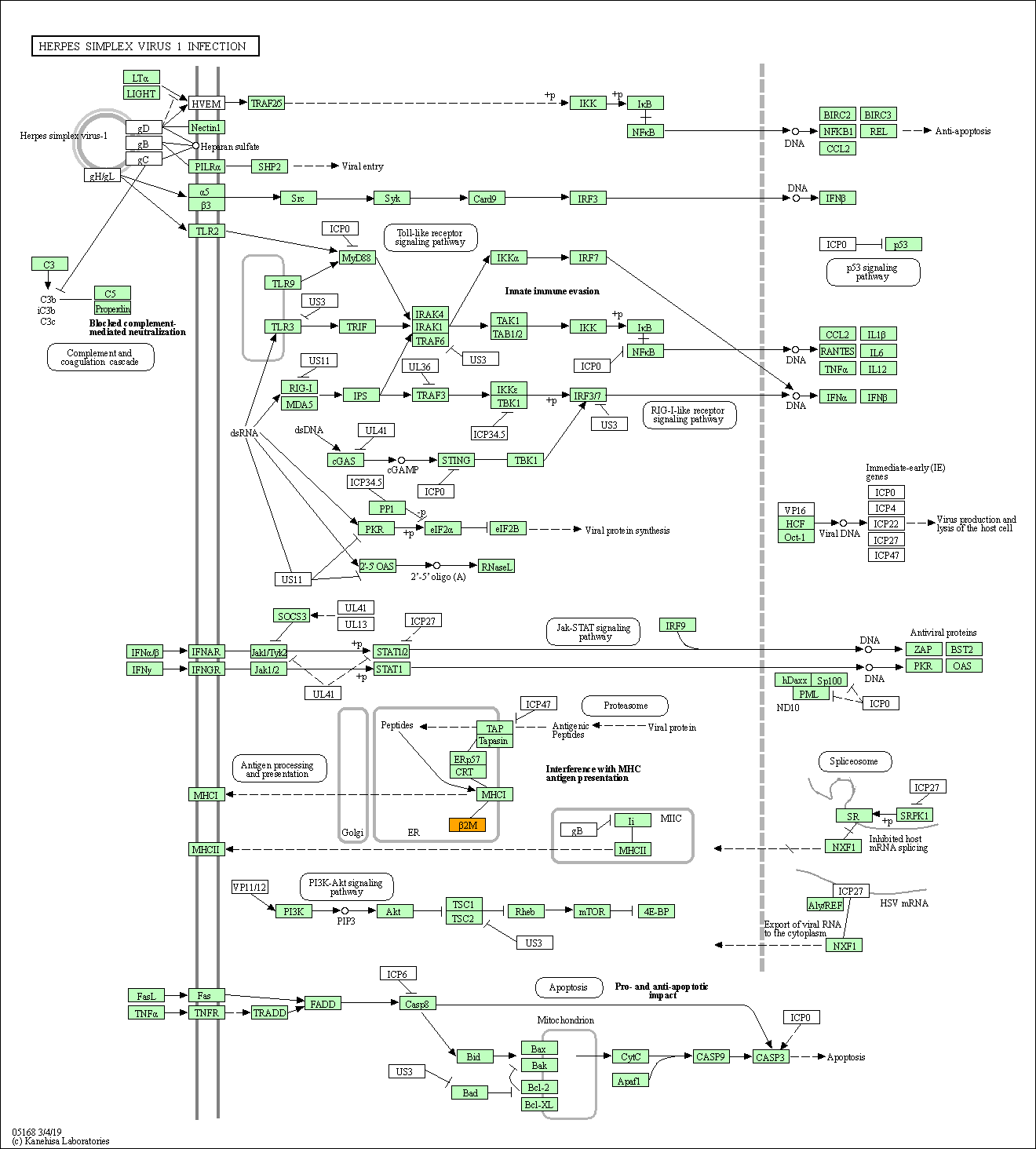

Supplement: Supplementary file 5 [file Data_Sheet_2.zip › Bioinformatics analysis related attachments/KEGG/Sample/png/bta05168.png]

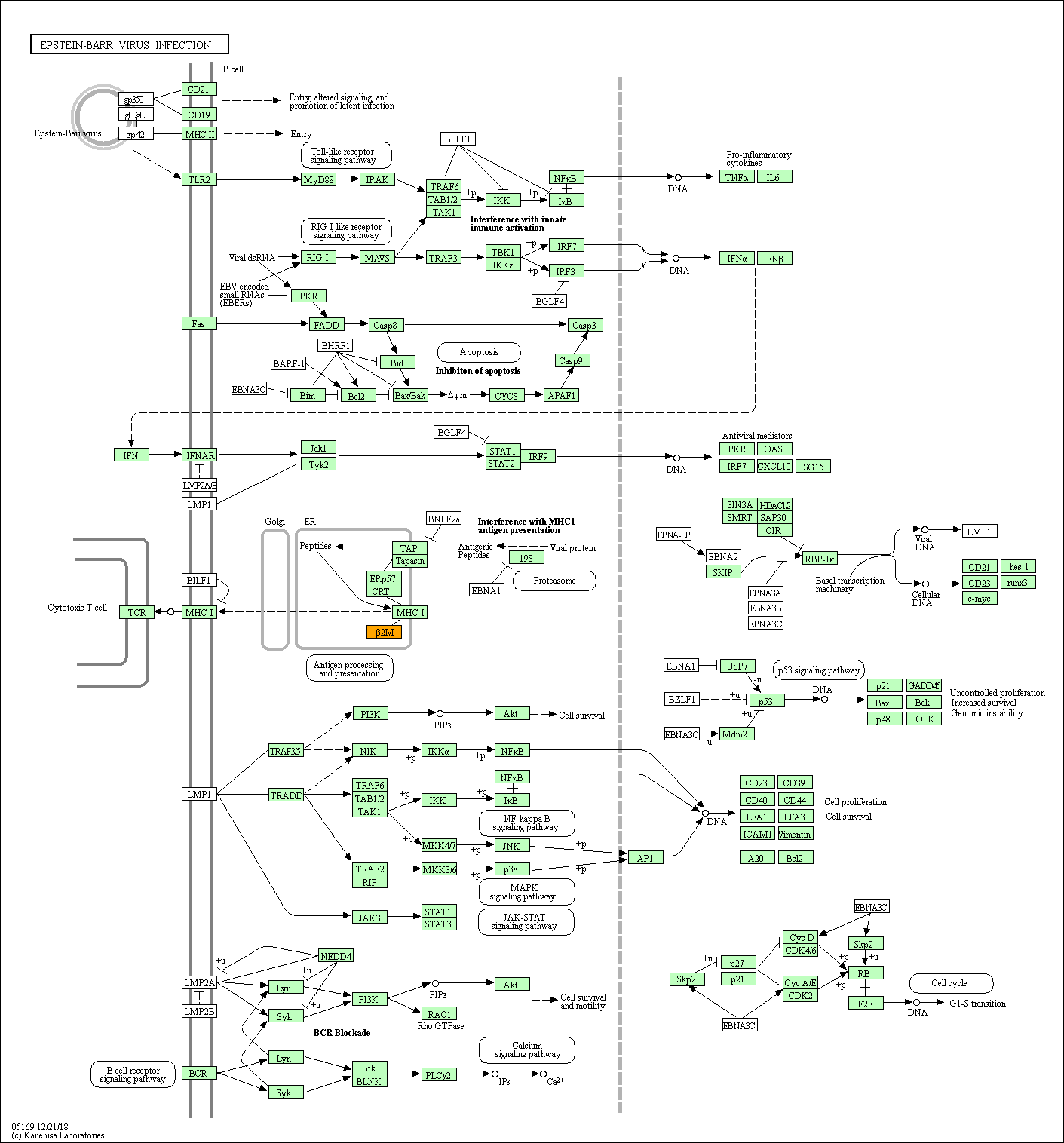

Supplement: Supplementary file 5 [file Data_Sheet_2.zip › Bioinformatics analysis related attachments/KEGG/Sample/png/bta05169.png]

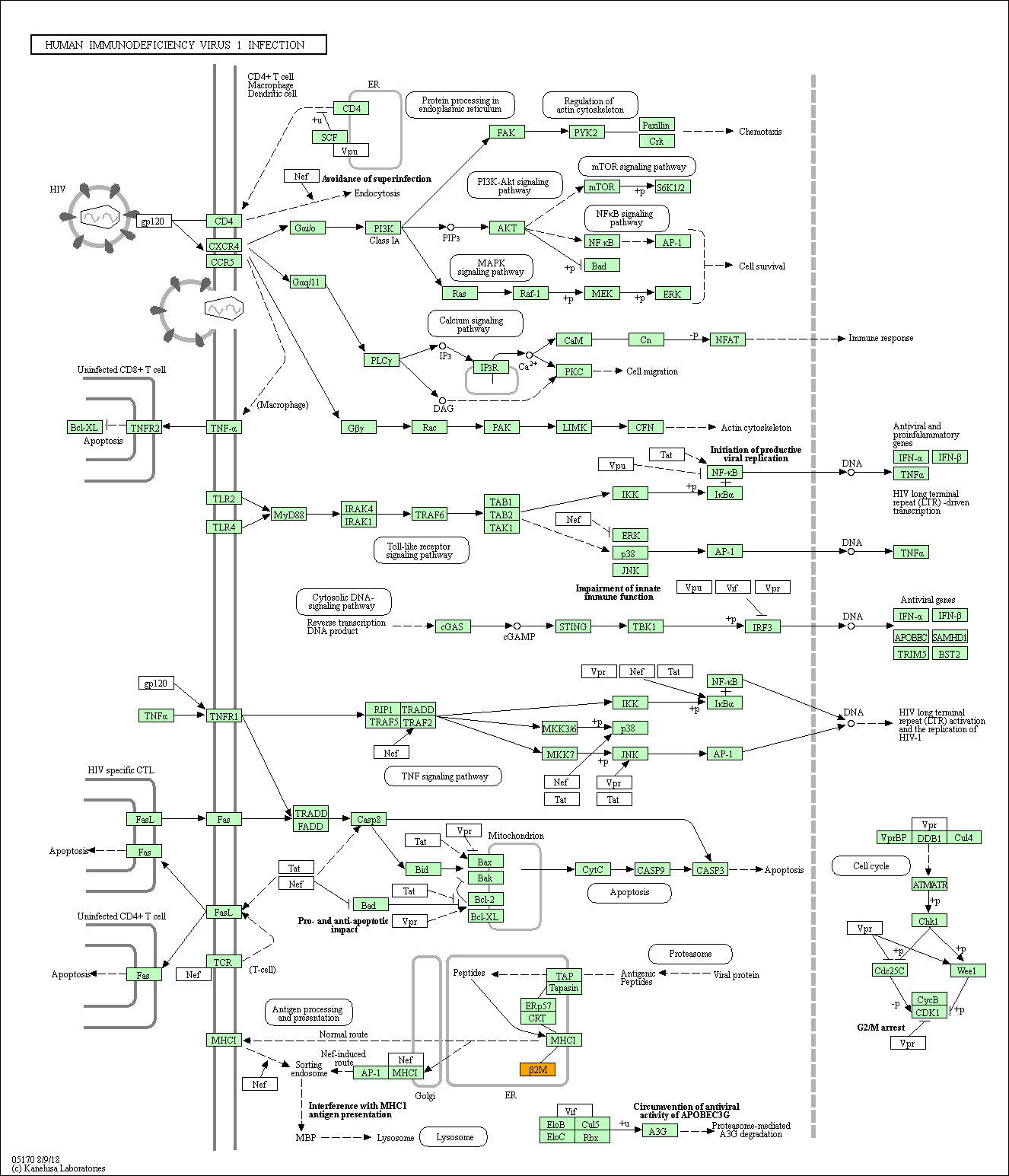

Supplement: Supplementary file 5 [file Data_Sheet_2.zip › Bioinformatics analysis related attachments/KEGG/Sample/png/bta05170.png]

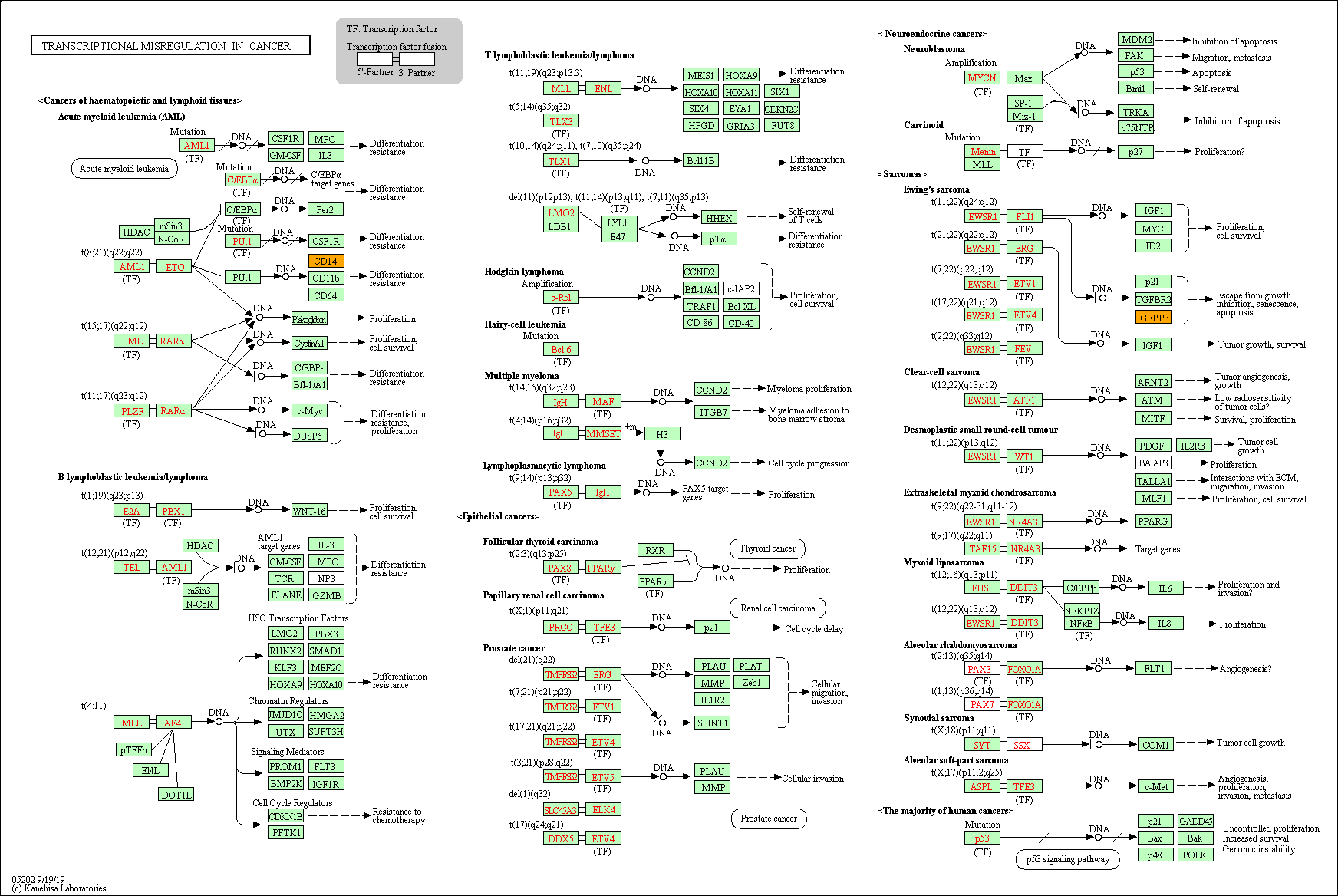

Supplement: Supplementary file 5 [file Data_Sheet_2.zip › Bioinformatics analysis related attachments/KEGG/Sample/png/bta05202.png]

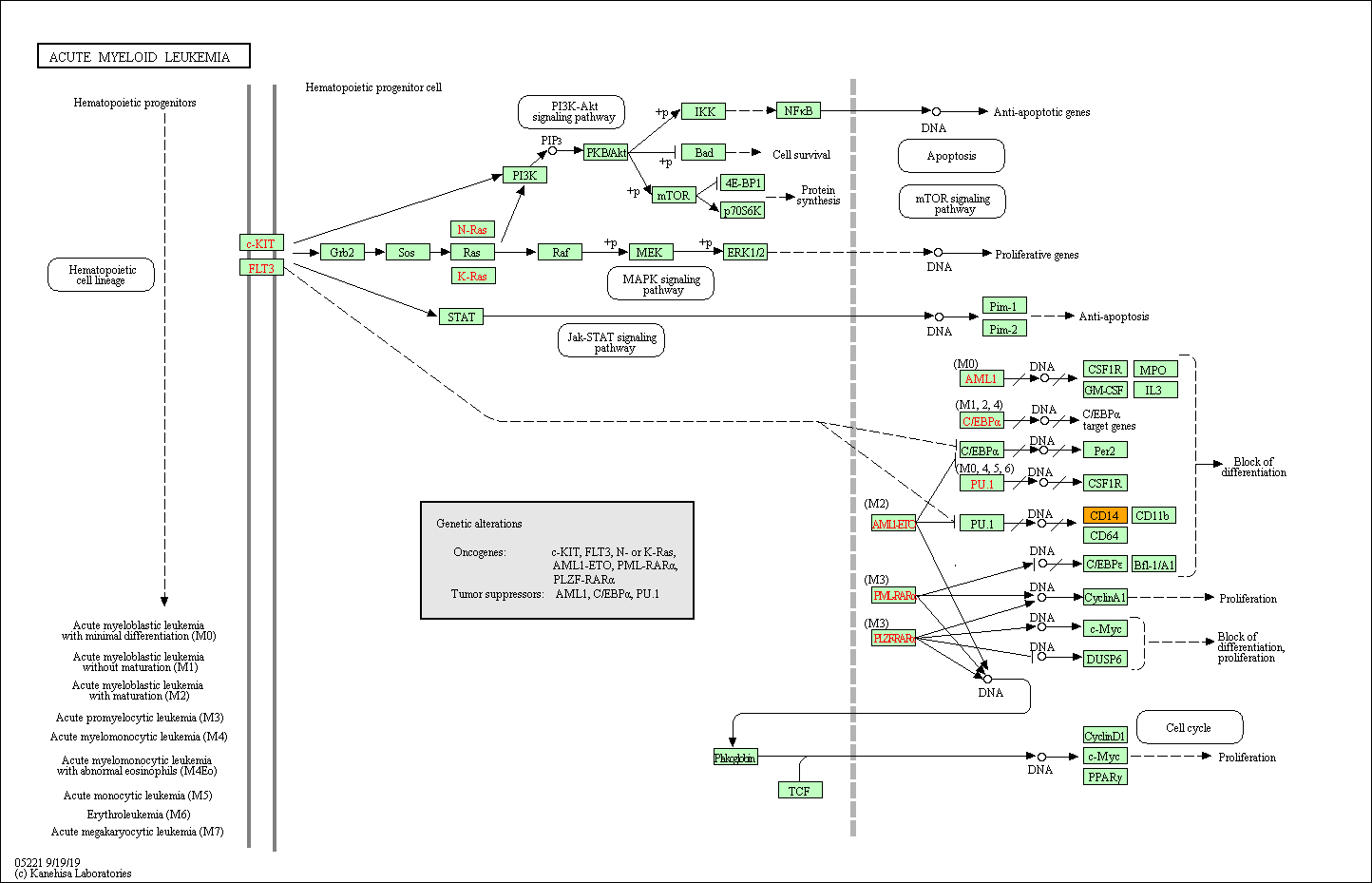

Supplement: Supplementary file 5 [file Data_Sheet_2.zip › Bioinformatics analysis related attachments/KEGG/Sample/png/bta05221.png]

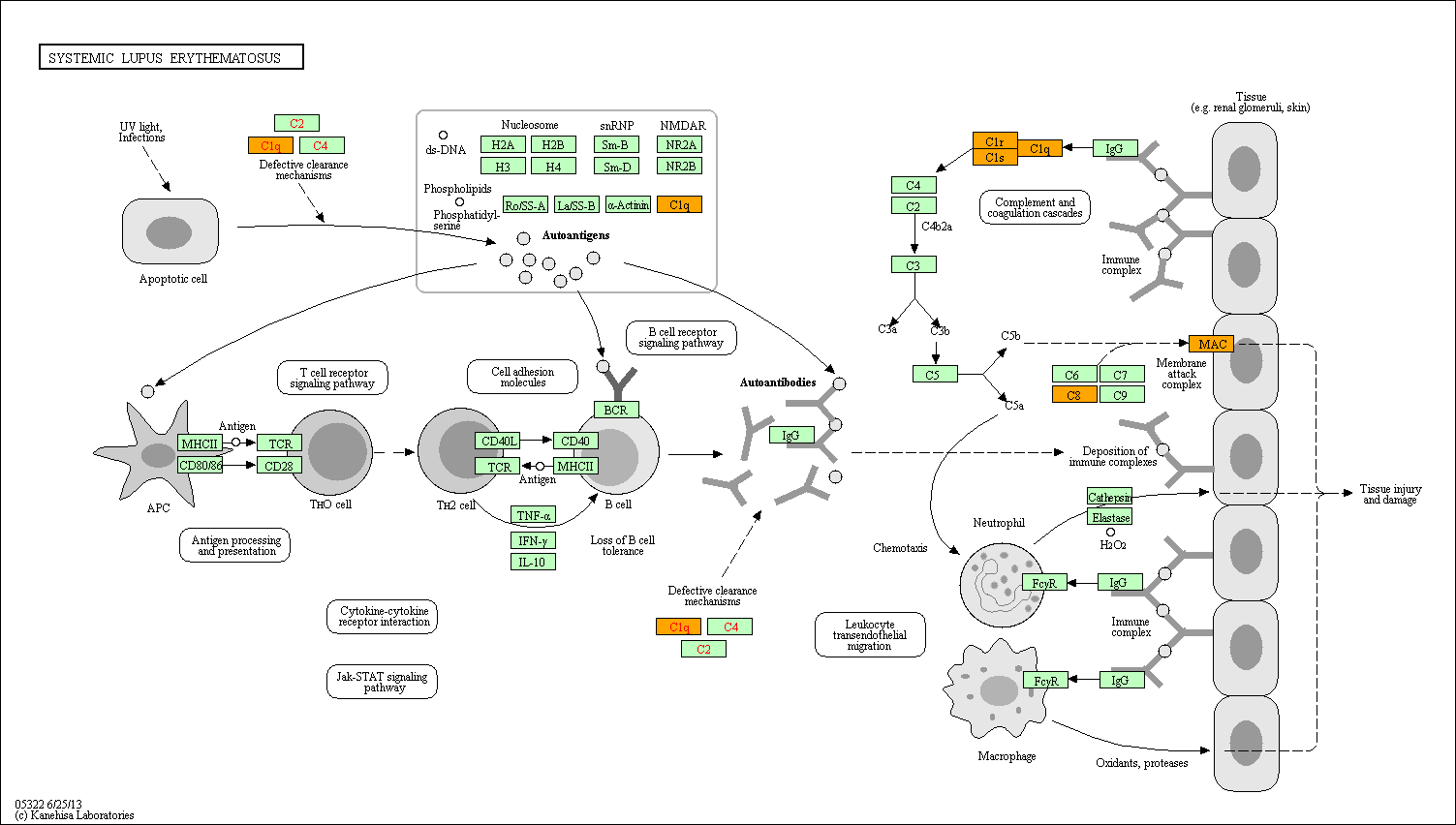

Supplement: Supplementary file 5 [file Data_Sheet_2.zip › Bioinformatics analysis related attachments/KEGG/Sample/png/bta05322.png]

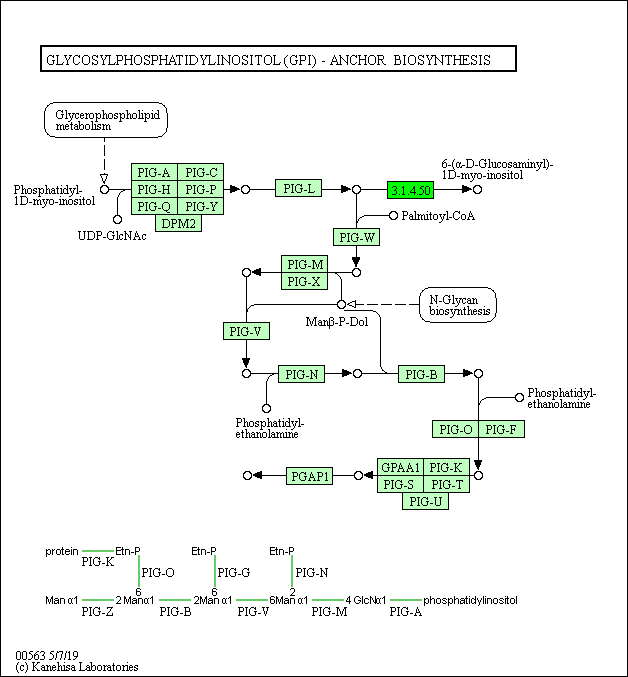

Supplement: Supplementary file 5 [file Data_Sheet_2.zip › Bioinformatics analysis related attachments/KEGG/Sig(QF_BQF)/png/bta00563.png]

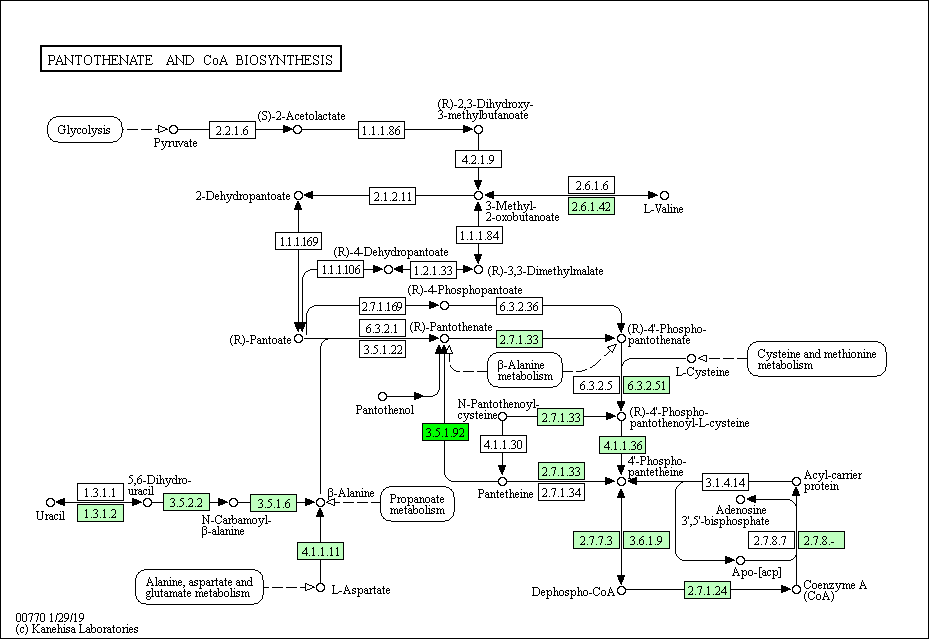

Supplement: Supplementary file 5 [file Data_Sheet_2.zip › Bioinformatics analysis related attachments/KEGG/Sig(QF_BQF)/png/bta00770.png]

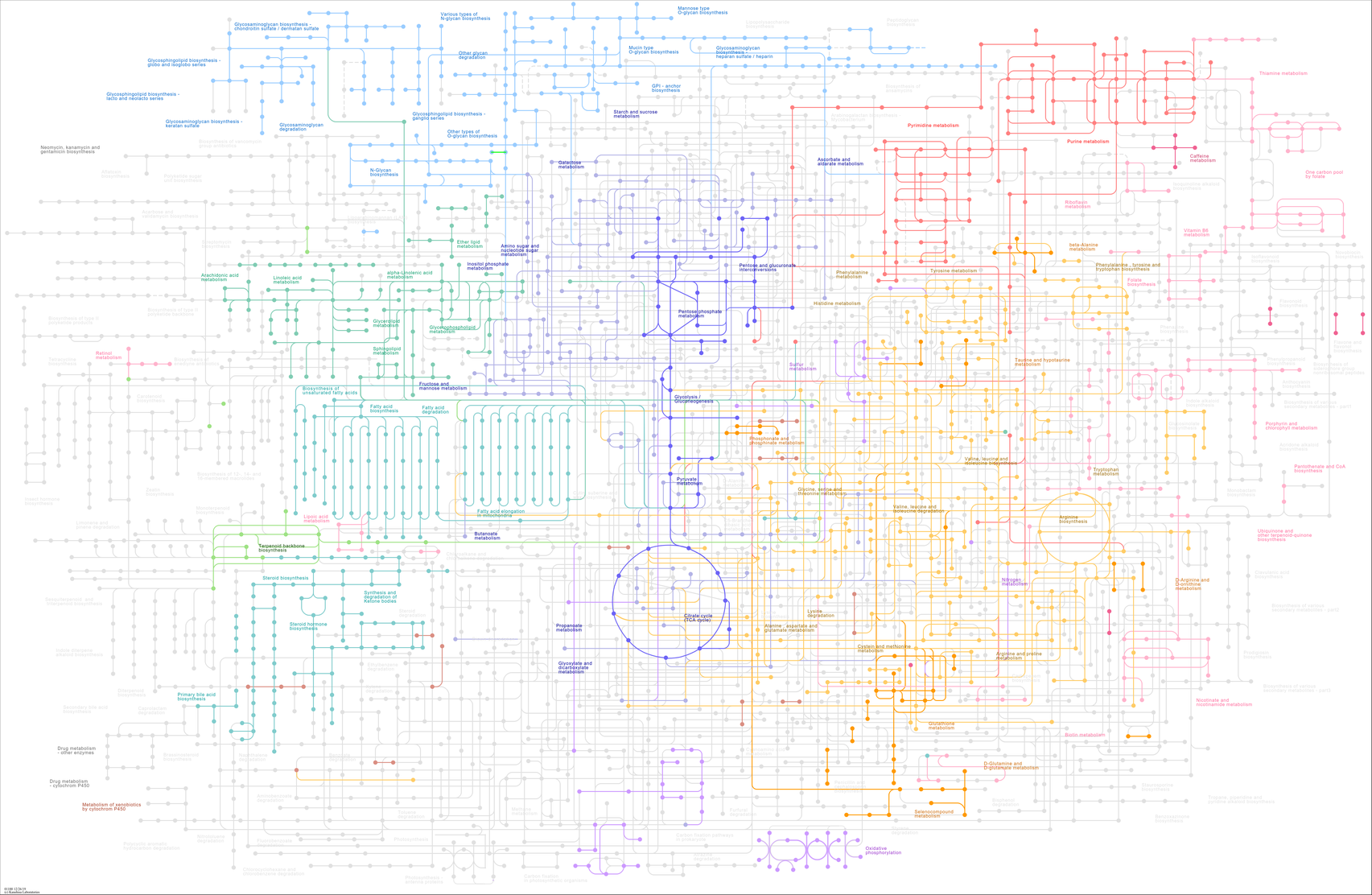

Supplement: Supplementary file 5 [file Data_Sheet_2.zip › Bioinformatics analysis related attachments/KEGG/Sig(QF_BQF)/png/bta01100.png]

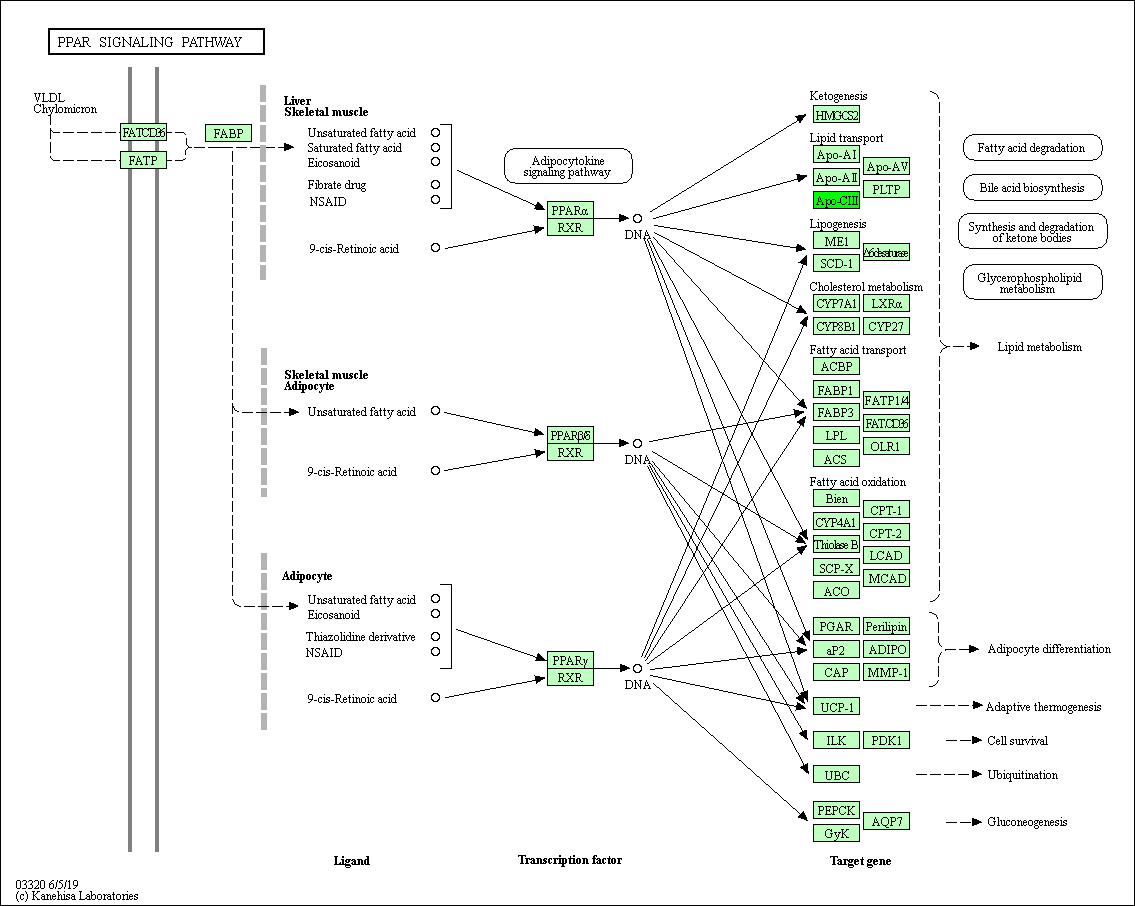

Supplement: Supplementary file 5 [file Data_Sheet_2.zip › Bioinformatics analysis related attachments/KEGG/Sig(QF_BQF)/png/bta03320.png]

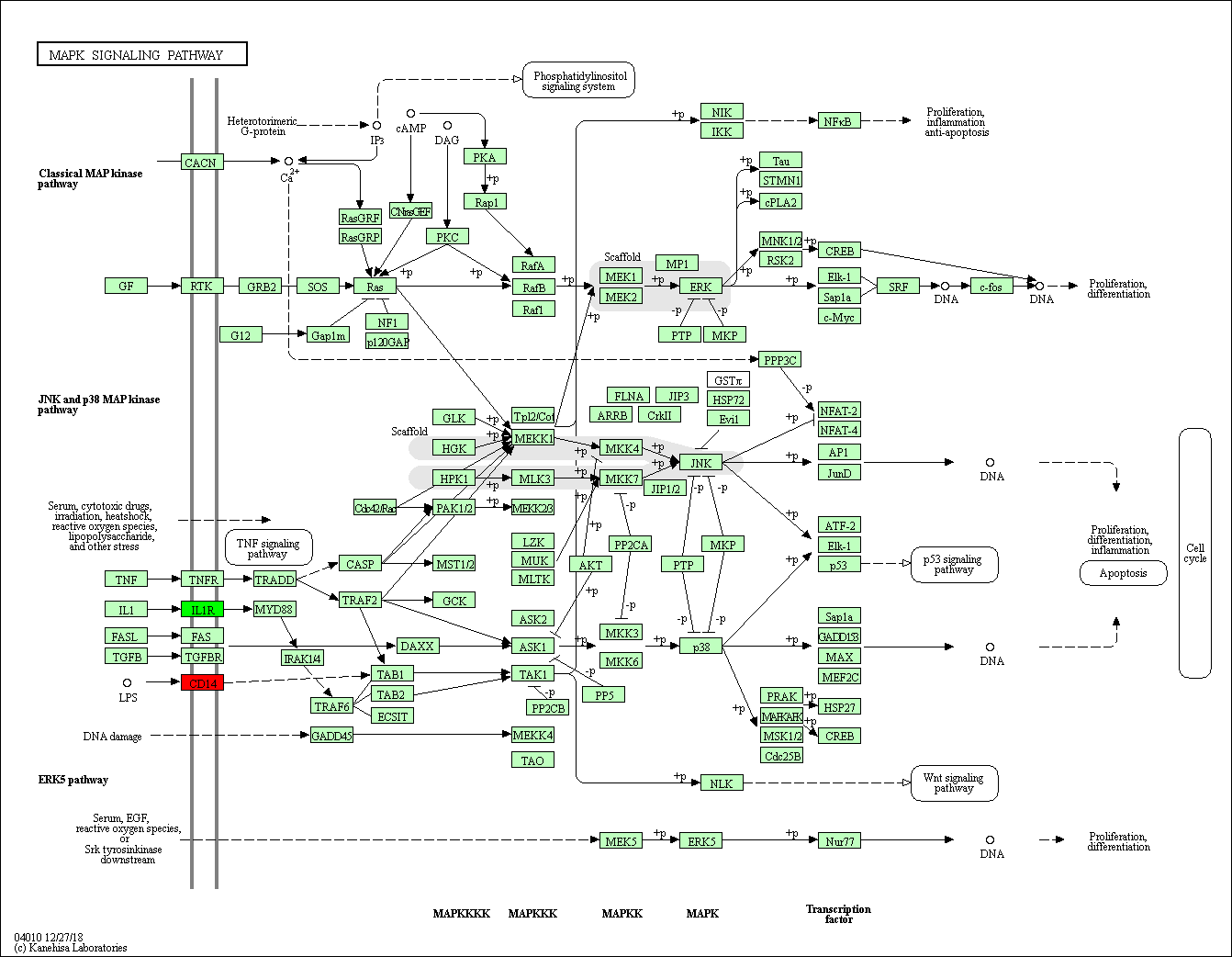

Supplement: Supplementary file 5 [file Data_Sheet_2.zip › Bioinformatics analysis related attachments/KEGG/Sig(QF_BQF)/png/bta04010.png]

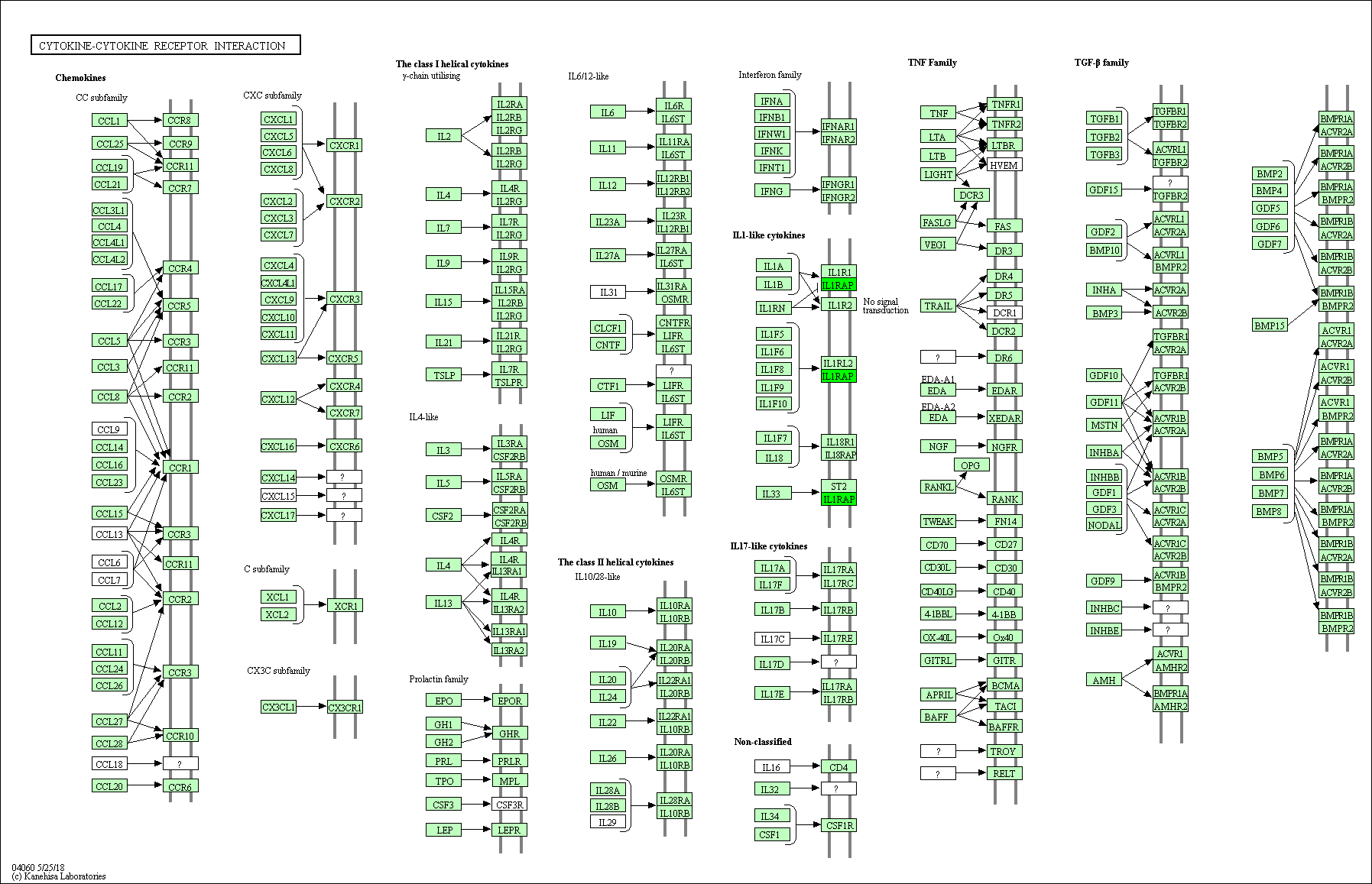

Supplement: Supplementary file 5 [file Data_Sheet_2.zip › Bioinformatics analysis related attachments/KEGG/Sig(QF_BQF)/png/bta04060.png]

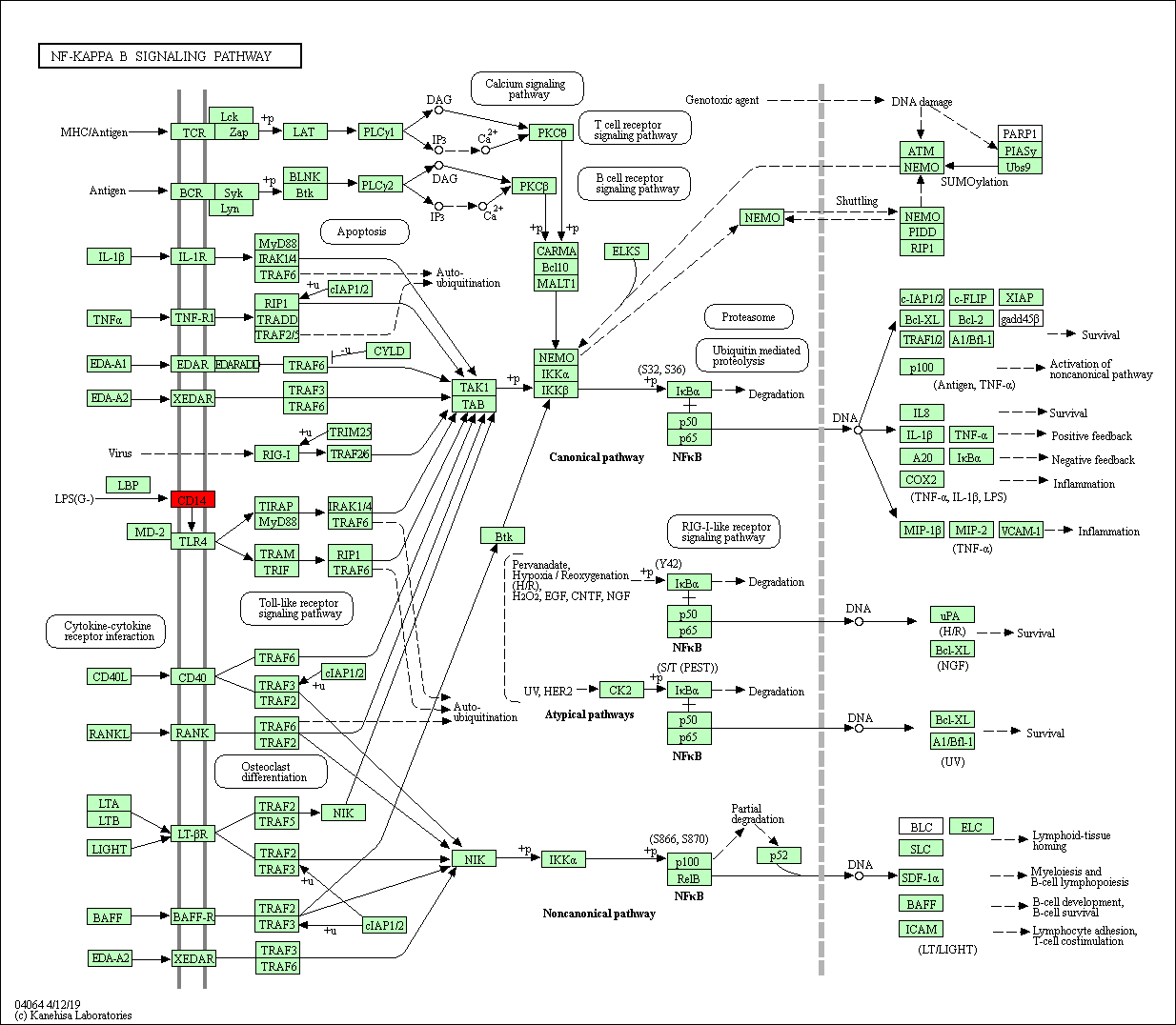

Supplement: Supplementary file 5 [file Data_Sheet_2.zip › Bioinformatics analysis related attachments/KEGG/Sig(QF_BQF)/png/bta04064.png]

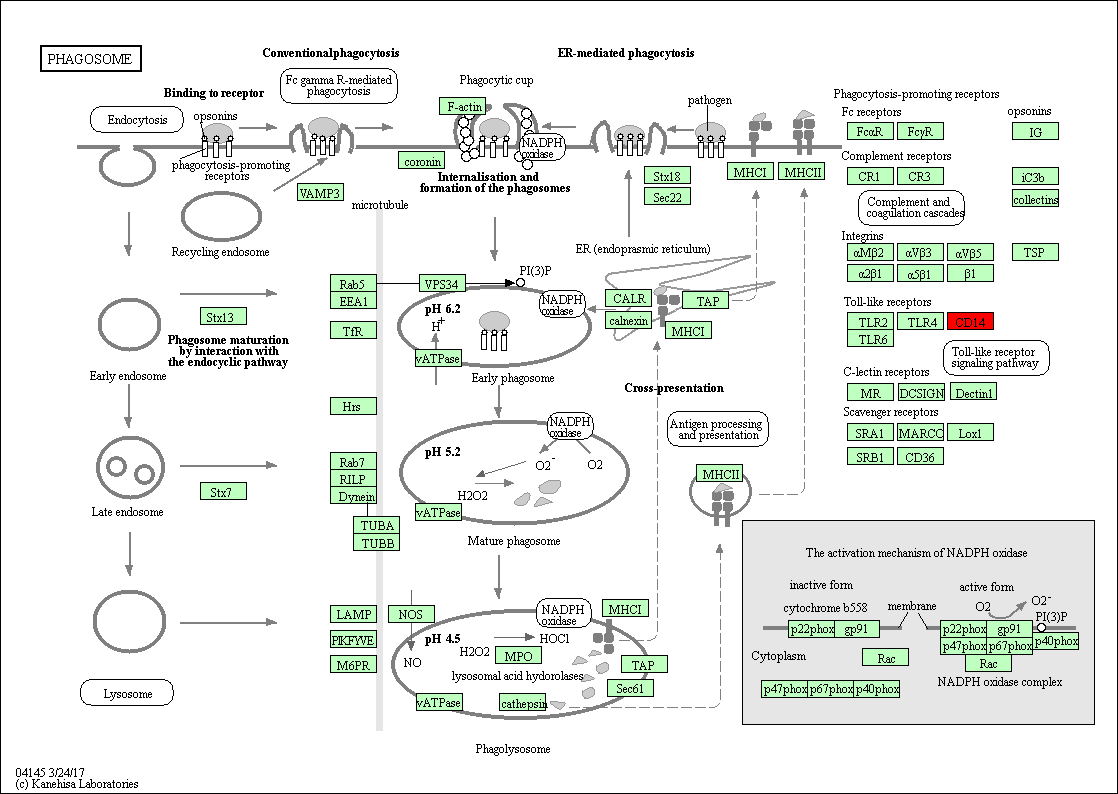

Supplement: Supplementary file 5 [file Data_Sheet_2.zip › Bioinformatics analysis related attachments/KEGG/Sig(QF_BQF)/png/bta04145.png]

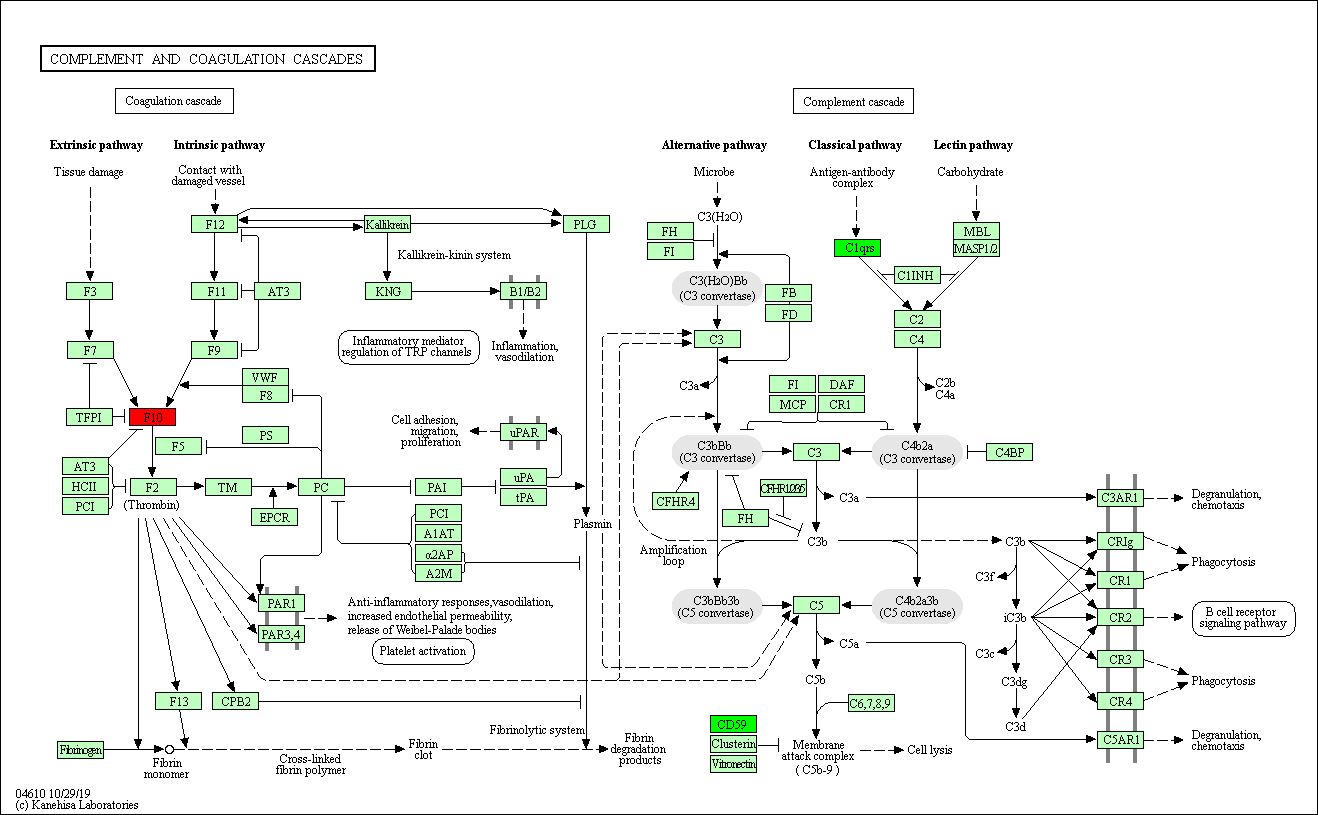

Supplement: Supplementary file 5 [file Data_Sheet_2.zip › Bioinformatics analysis related attachments/KEGG/Sig(QF_BQF)/png/bta04610.png]

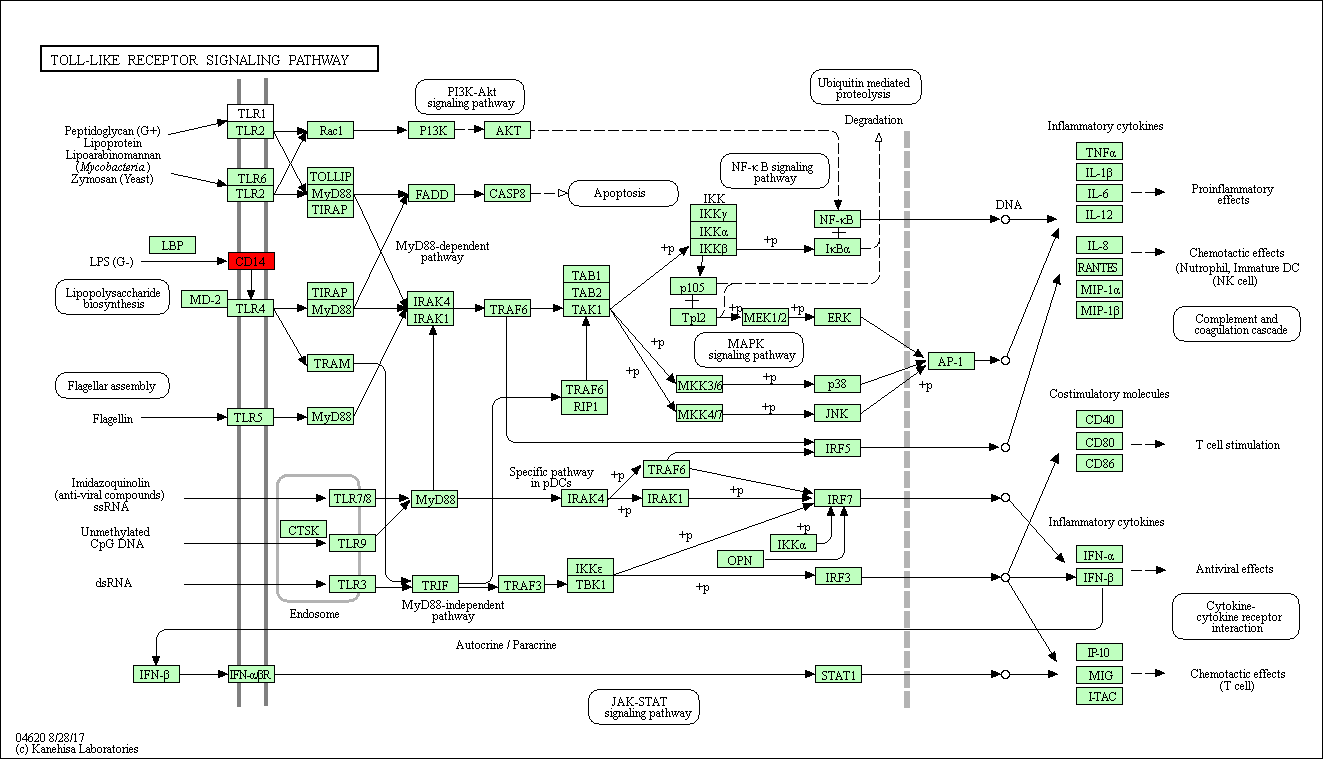

Supplement: Supplementary file 5 [file Data_Sheet_2.zip › Bioinformatics analysis related attachments/KEGG/Sig(QF_BQF)/png/bta04620.png]

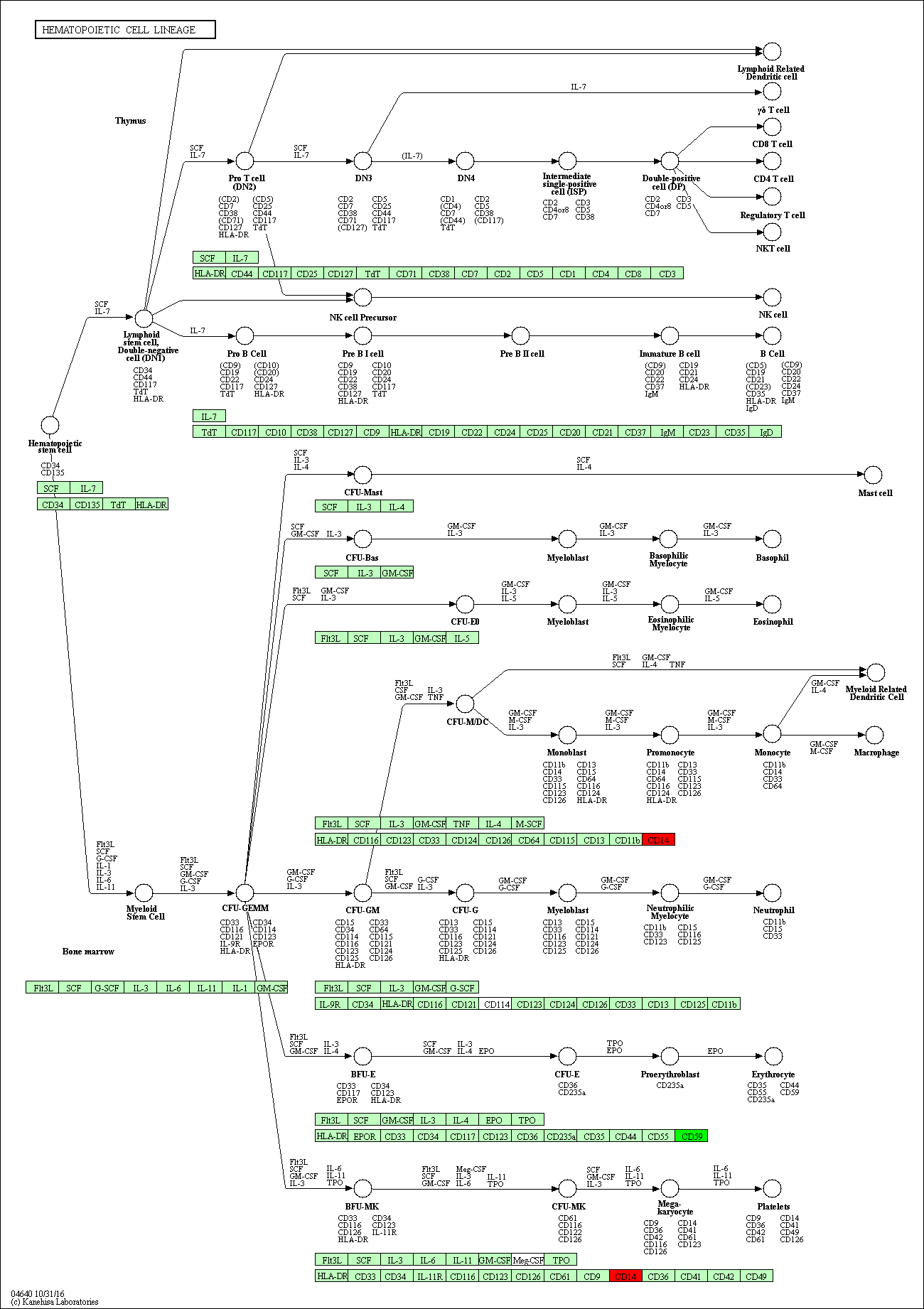

Supplement: Supplementary file 5 [file Data_Sheet_2.zip › Bioinformatics analysis related attachments/KEGG/Sig(QF_BQF)/png/bta04640.png]

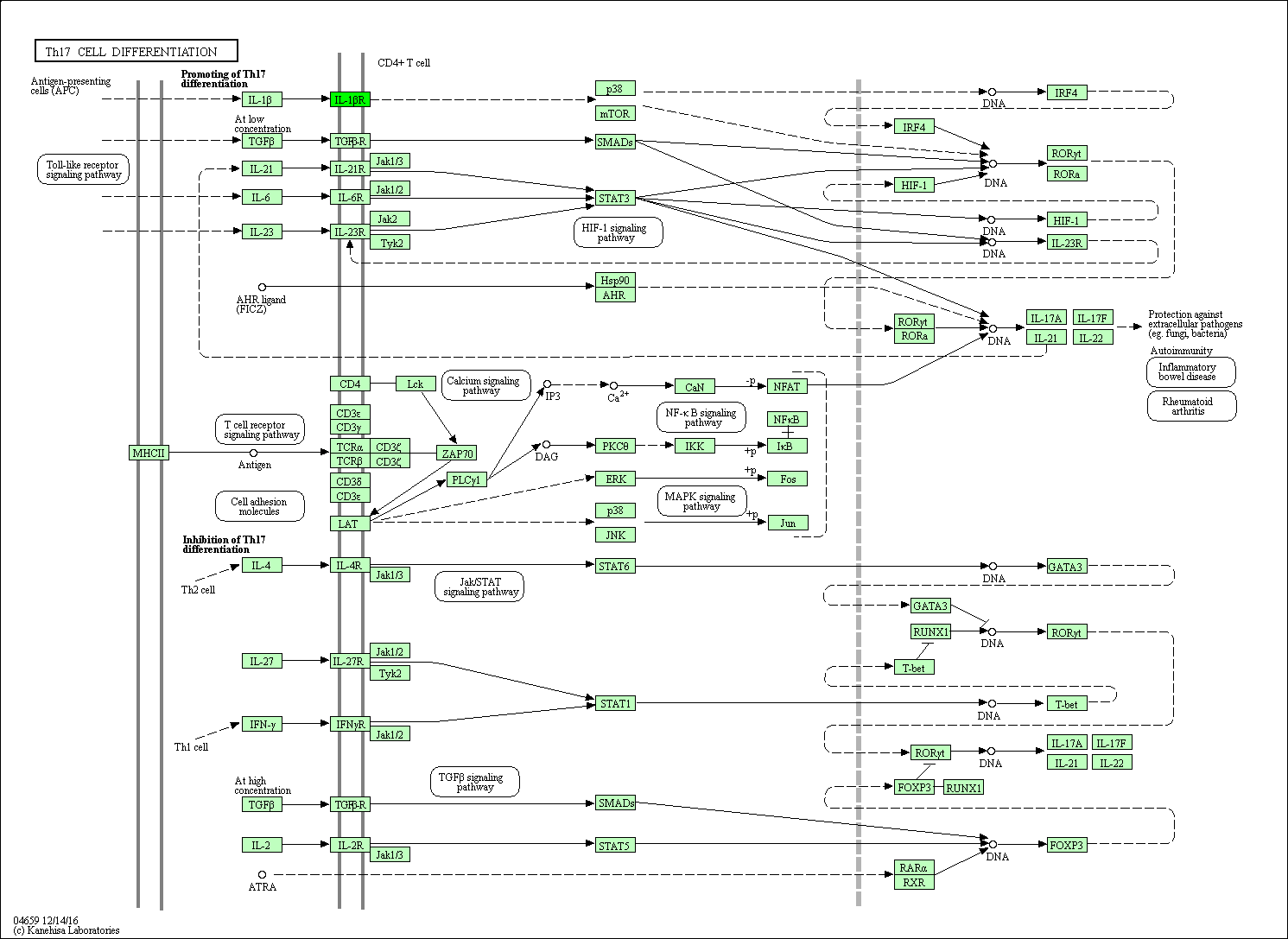

Supplement: Supplementary file 5 [file Data_Sheet_2.zip › Bioinformatics analysis related attachments/KEGG/Sig(QF_BQF)/png/bta04659.png]

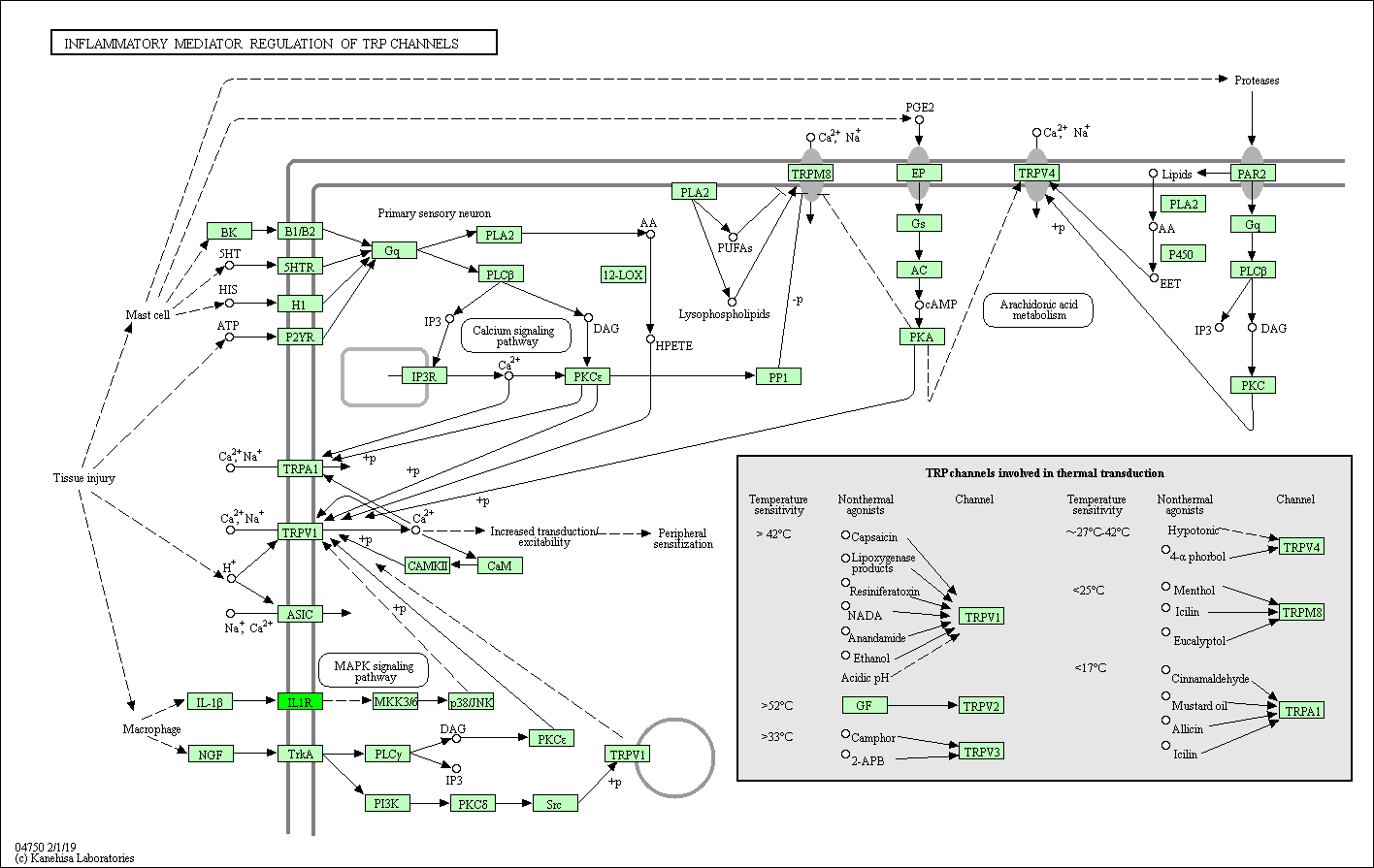

Supplement: Supplementary file 5 [file Data_Sheet_2.zip › Bioinformatics analysis related attachments/KEGG/Sig(QF_BQF)/png/bta04750.png]

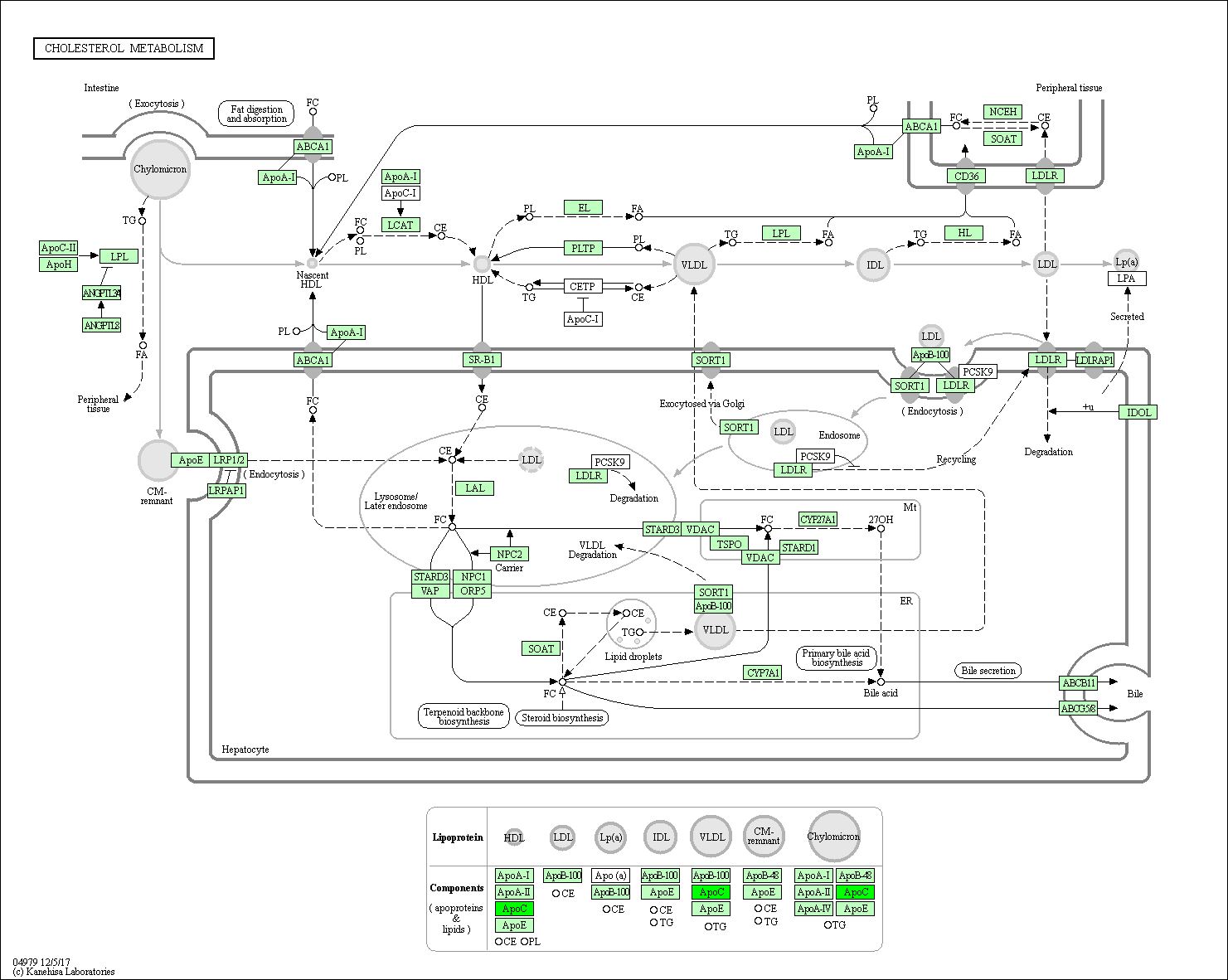

Supplement: Supplementary file 5 [file Data_Sheet_2.zip › Bioinformatics analysis related attachments/KEGG/Sig(QF_BQF)/png/bta04979.png]

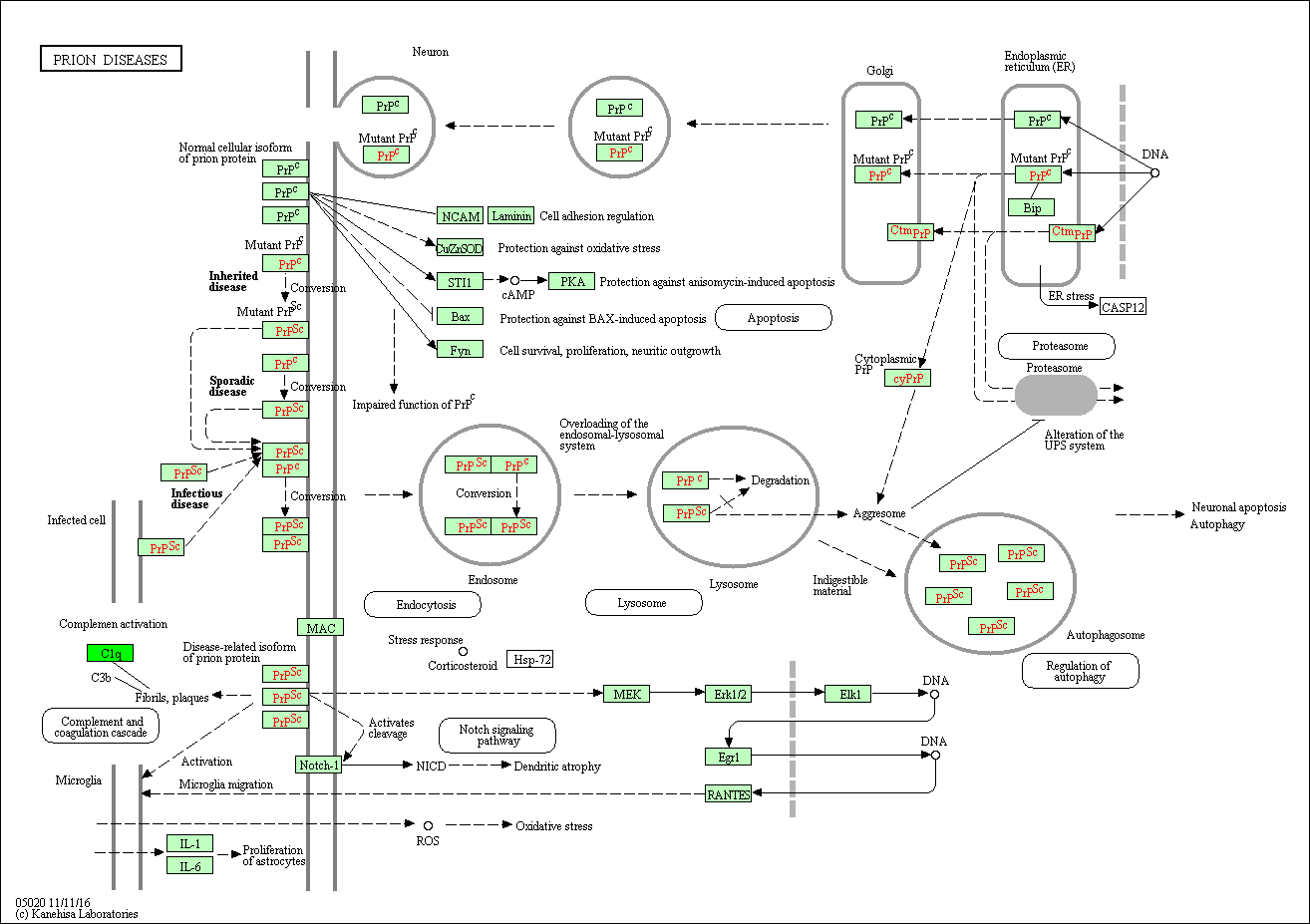

Supplement: Supplementary file 5 [file Data_Sheet_2.zip › Bioinformatics analysis related attachments/KEGG/Sig(QF_BQF)/png/bta05020.png]

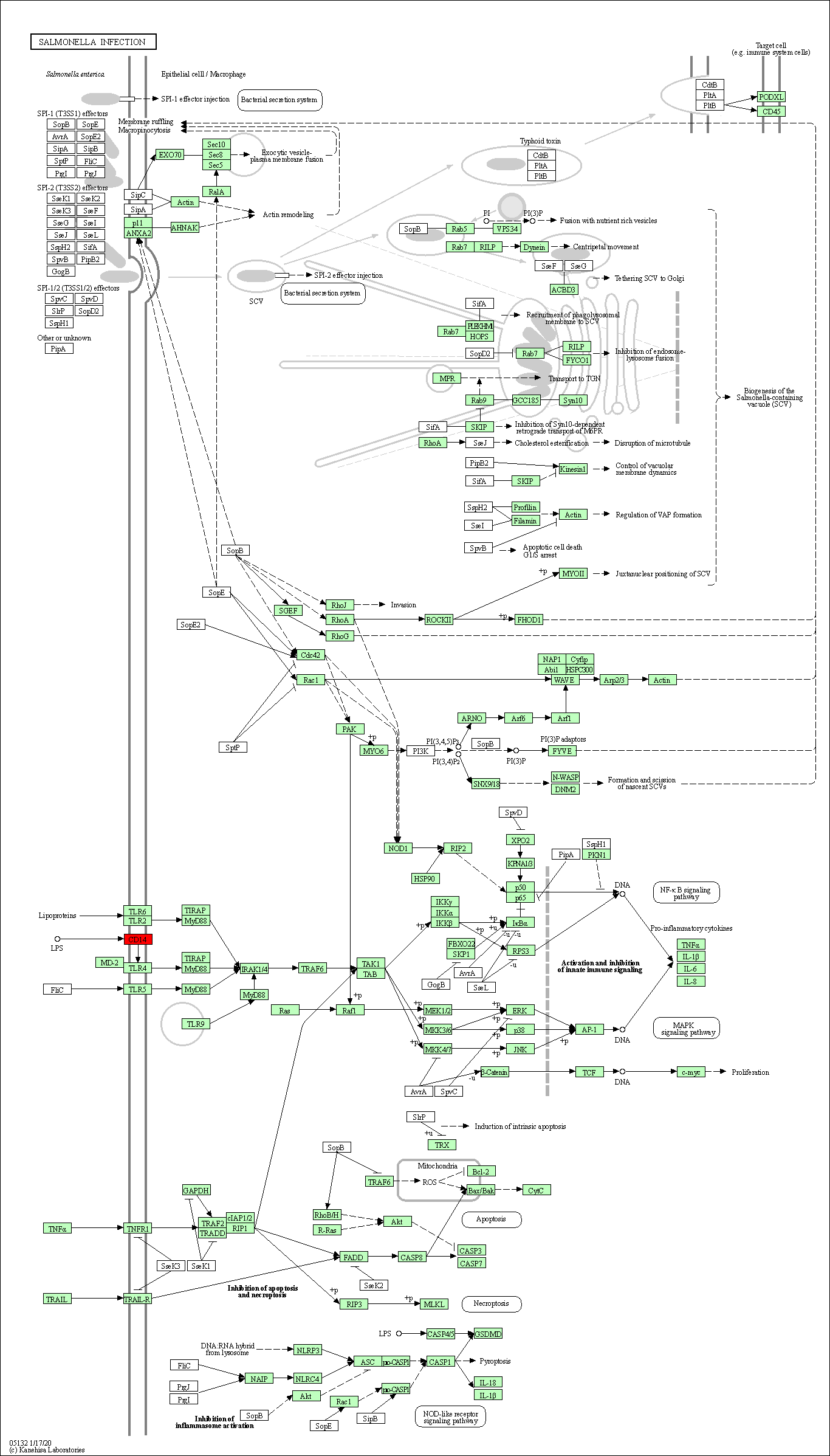

Supplement: Supplementary file 5 [file Data_Sheet_2.zip › Bioinformatics analysis related attachments/KEGG/Sig(QF_BQF)/png/bta05132.png]

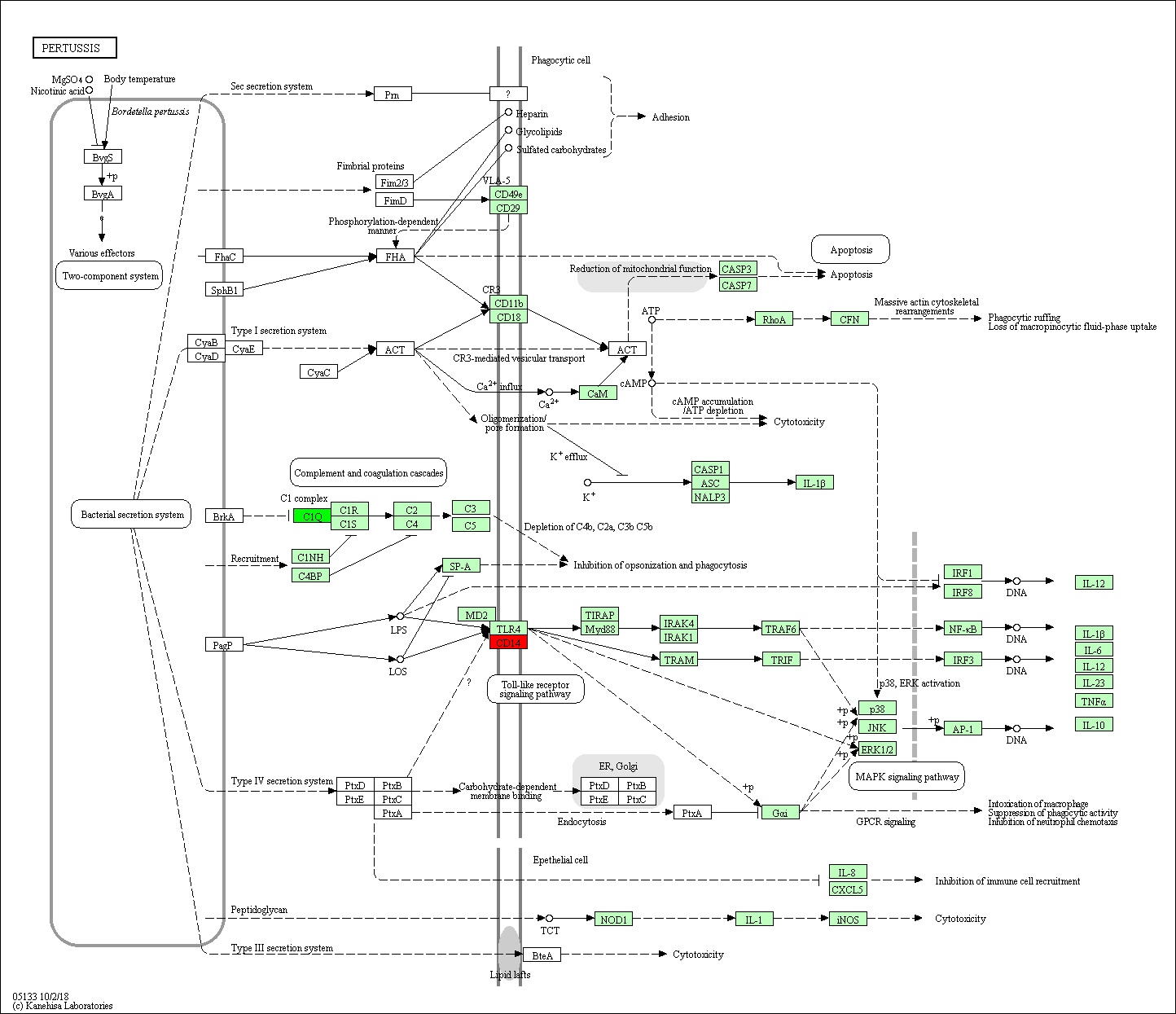

Supplement: Supplementary file 5 [file Data_Sheet_2.zip › Bioinformatics analysis related attachments/KEGG/Sig(QF_BQF)/png/bta05133.png]

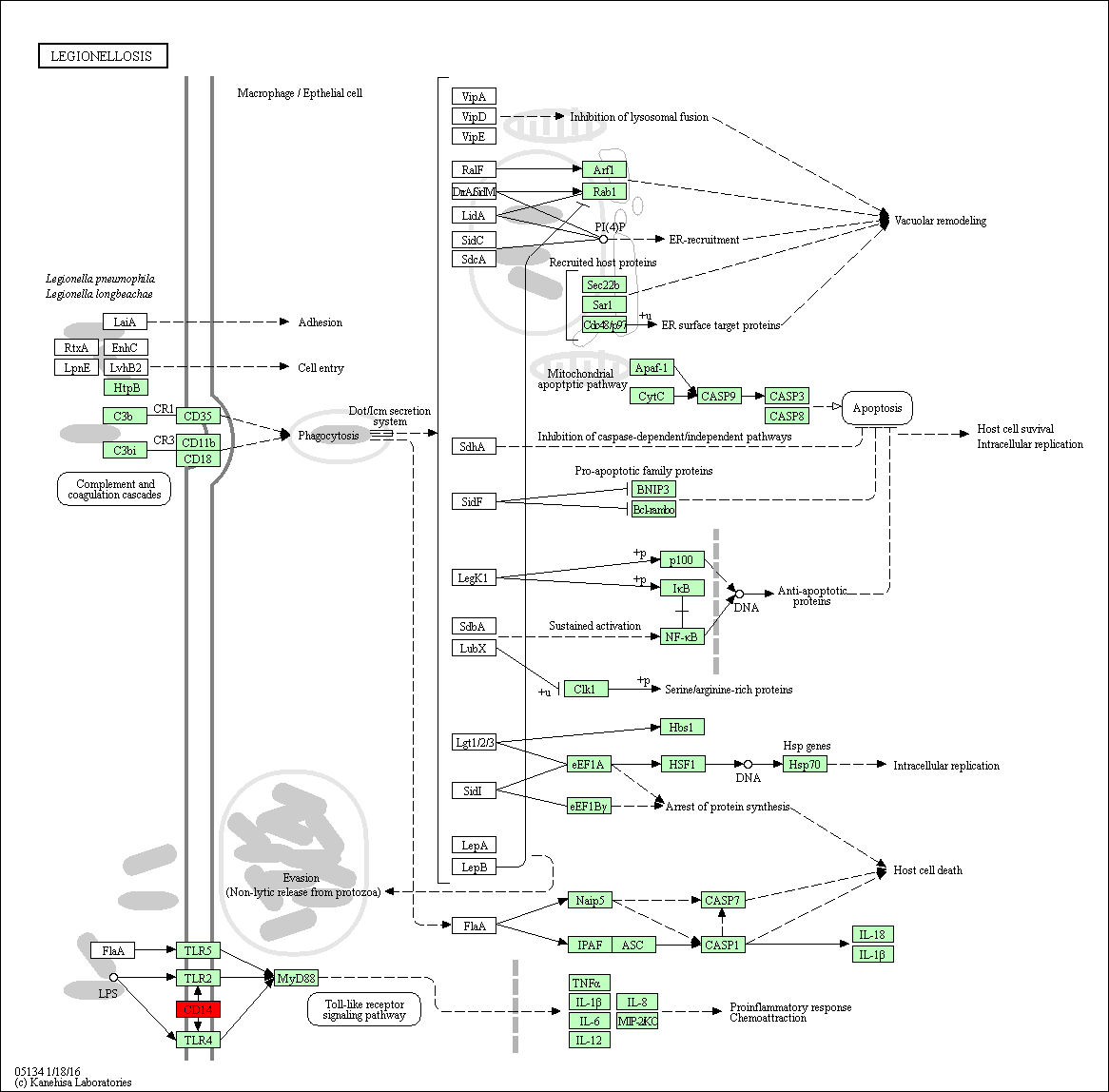

Supplement: Supplementary file 5 [file Data_Sheet_2.zip › Bioinformatics analysis related attachments/KEGG/Sig(QF_BQF)/png/bta05134.png]

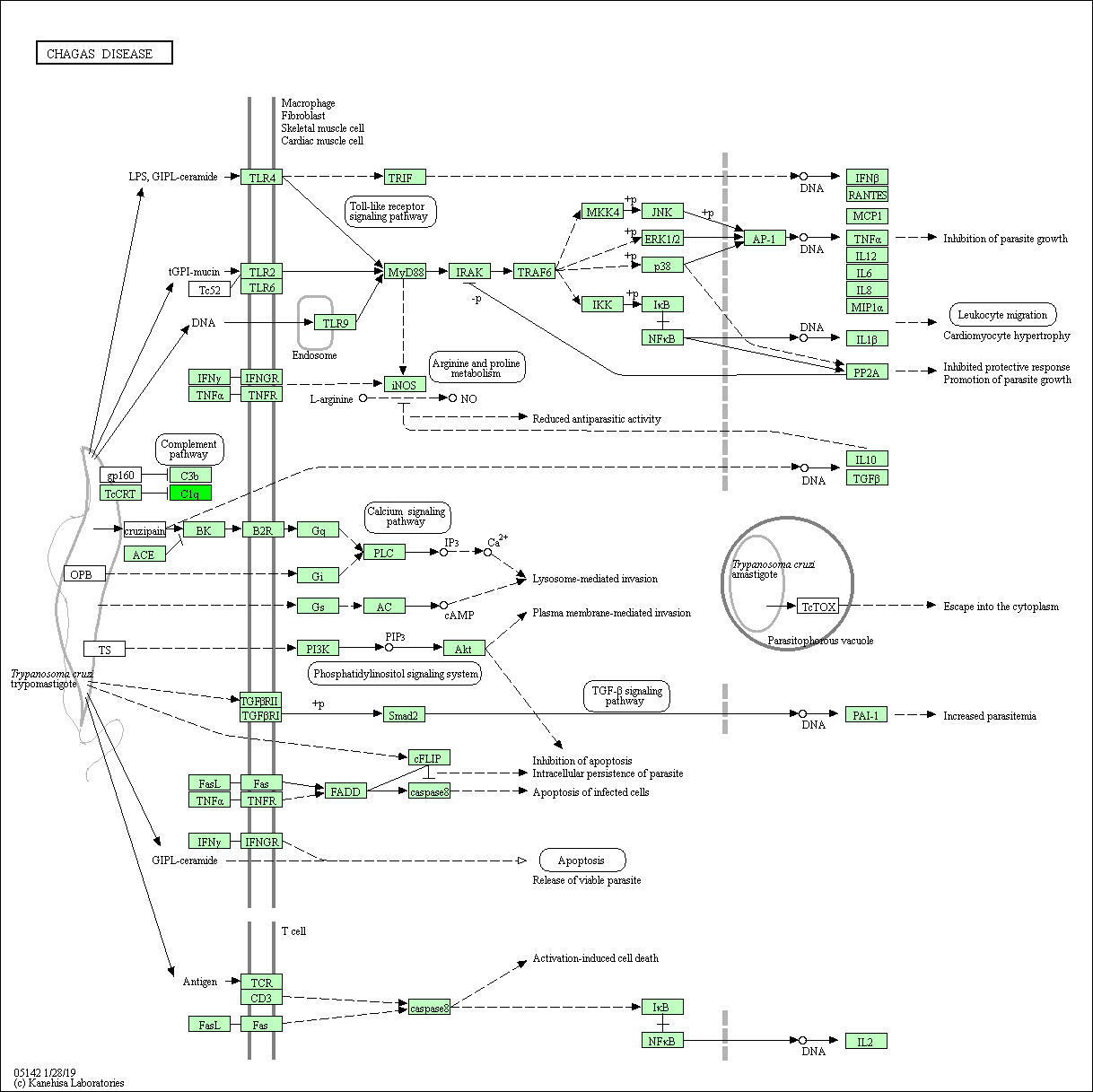

Supplement: Supplementary file 5 [file Data_Sheet_2.zip › Bioinformatics analysis related attachments/KEGG/Sig(QF_BQF)/png/bta05142.png]

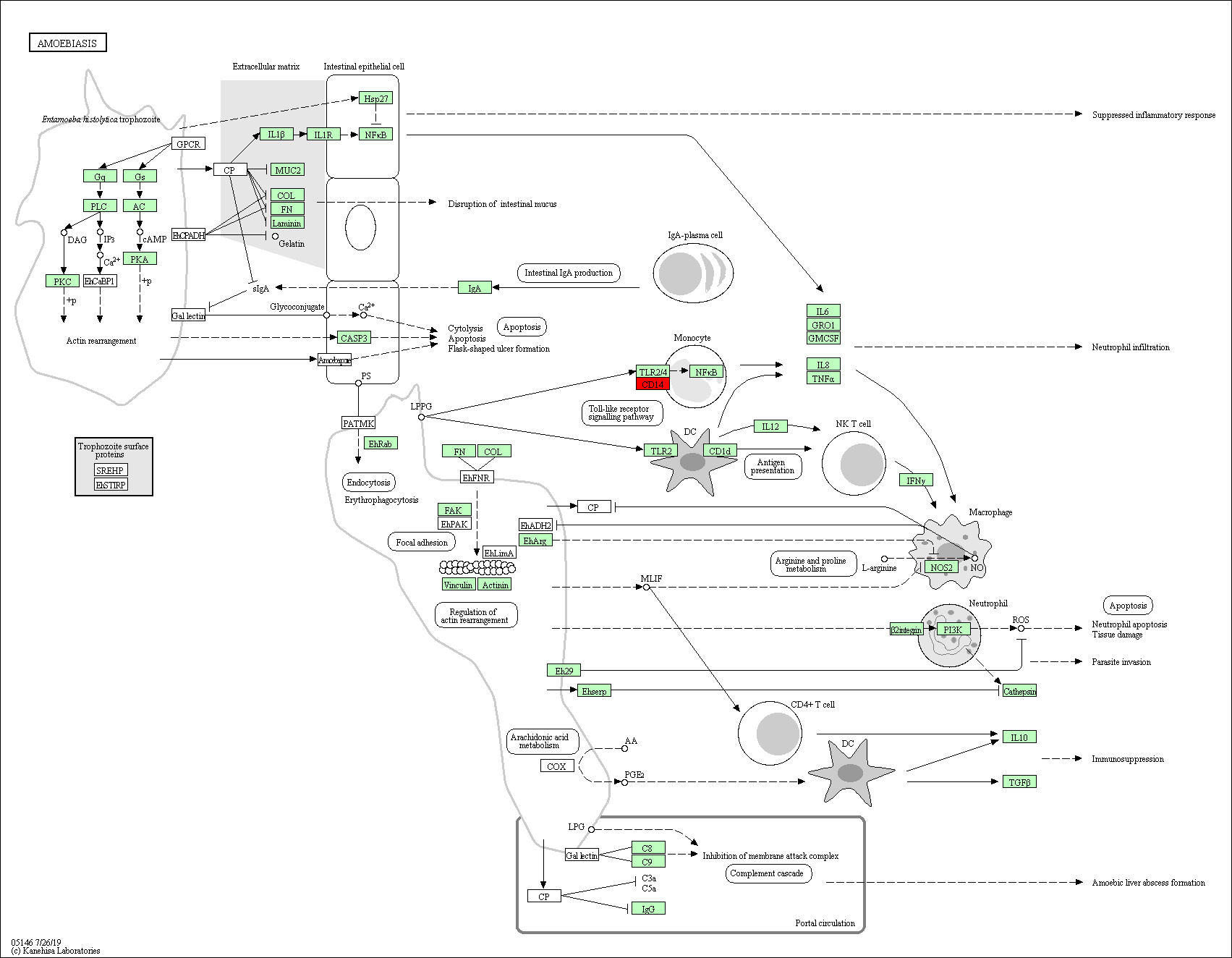

Supplement: Supplementary file 5 [file Data_Sheet_2.zip › Bioinformatics analysis related attachments/KEGG/Sig(QF_BQF)/png/bta05146.png]

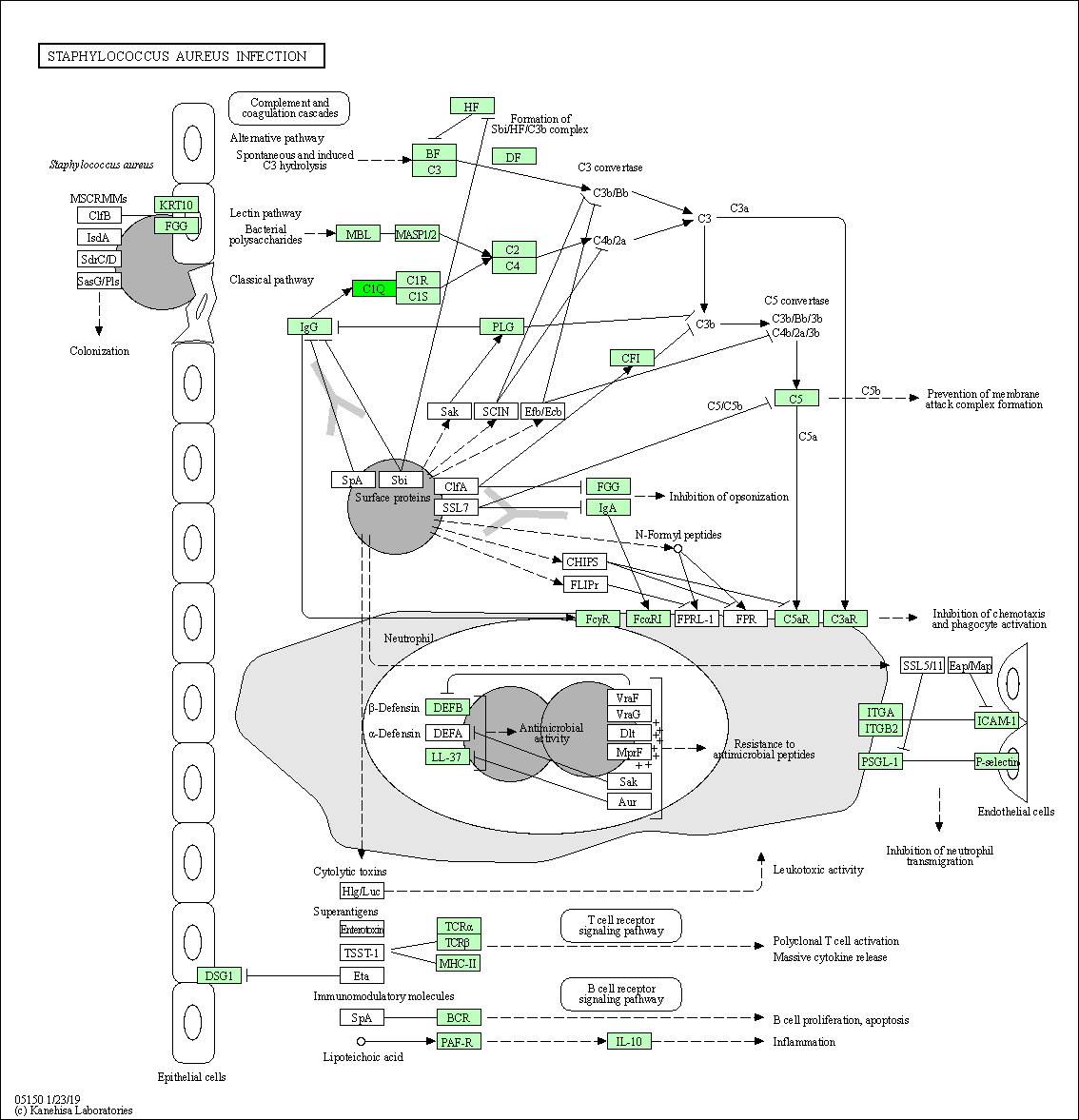

Supplement: Supplementary file 5 [file Data_Sheet_2.zip › Bioinformatics analysis related attachments/KEGG/Sig(QF_BQF)/png/bta05150.png]

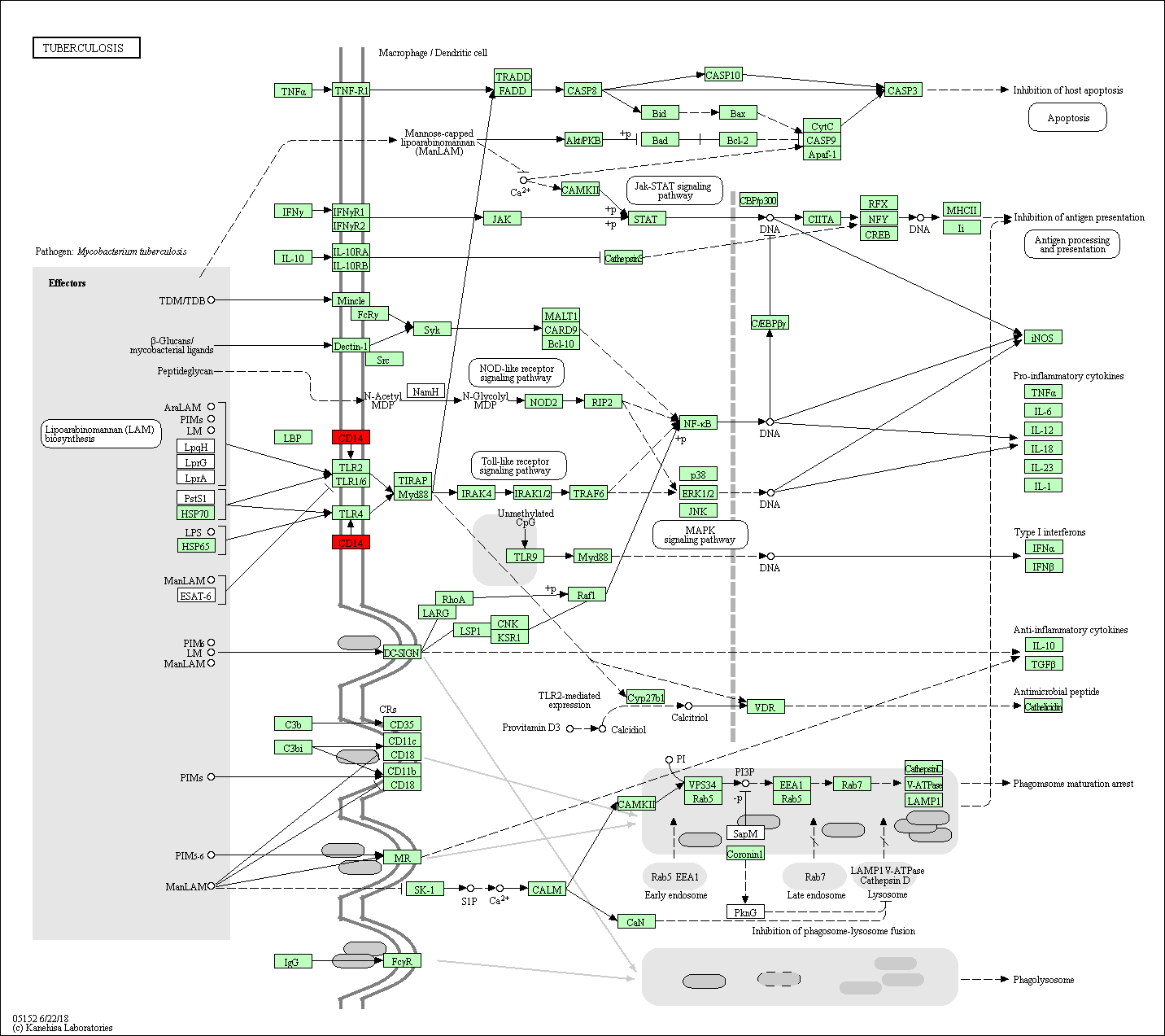

Supplement: Supplementary file 5 [file Data_Sheet_2.zip › Bioinformatics analysis related attachments/KEGG/Sig(QF_BQF)/png/bta05152.png]

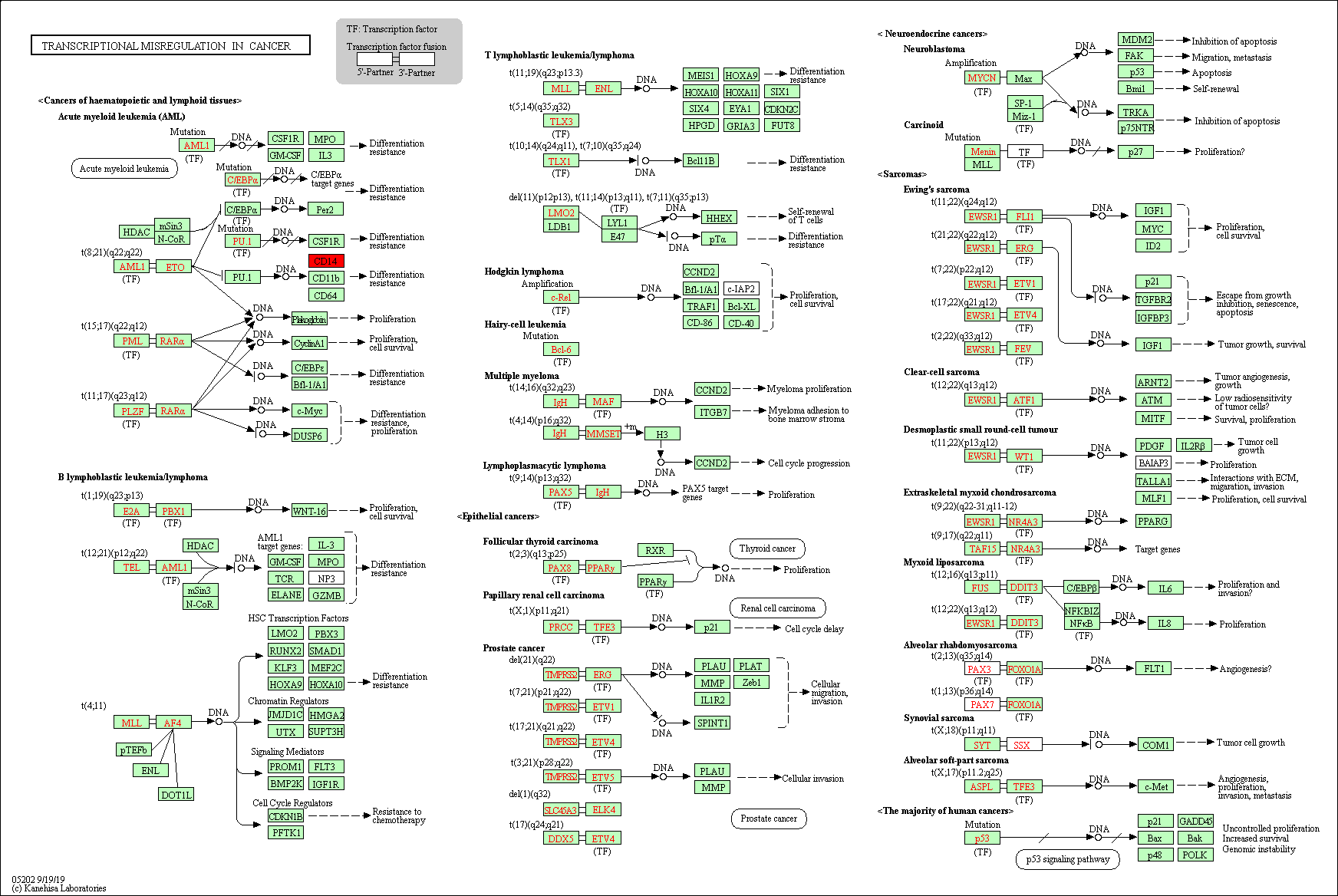

Supplement: Supplementary file 5 [file Data_Sheet_2.zip › Bioinformatics analysis related attachments/KEGG/Sig(QF_BQF)/png/bta05202.png]

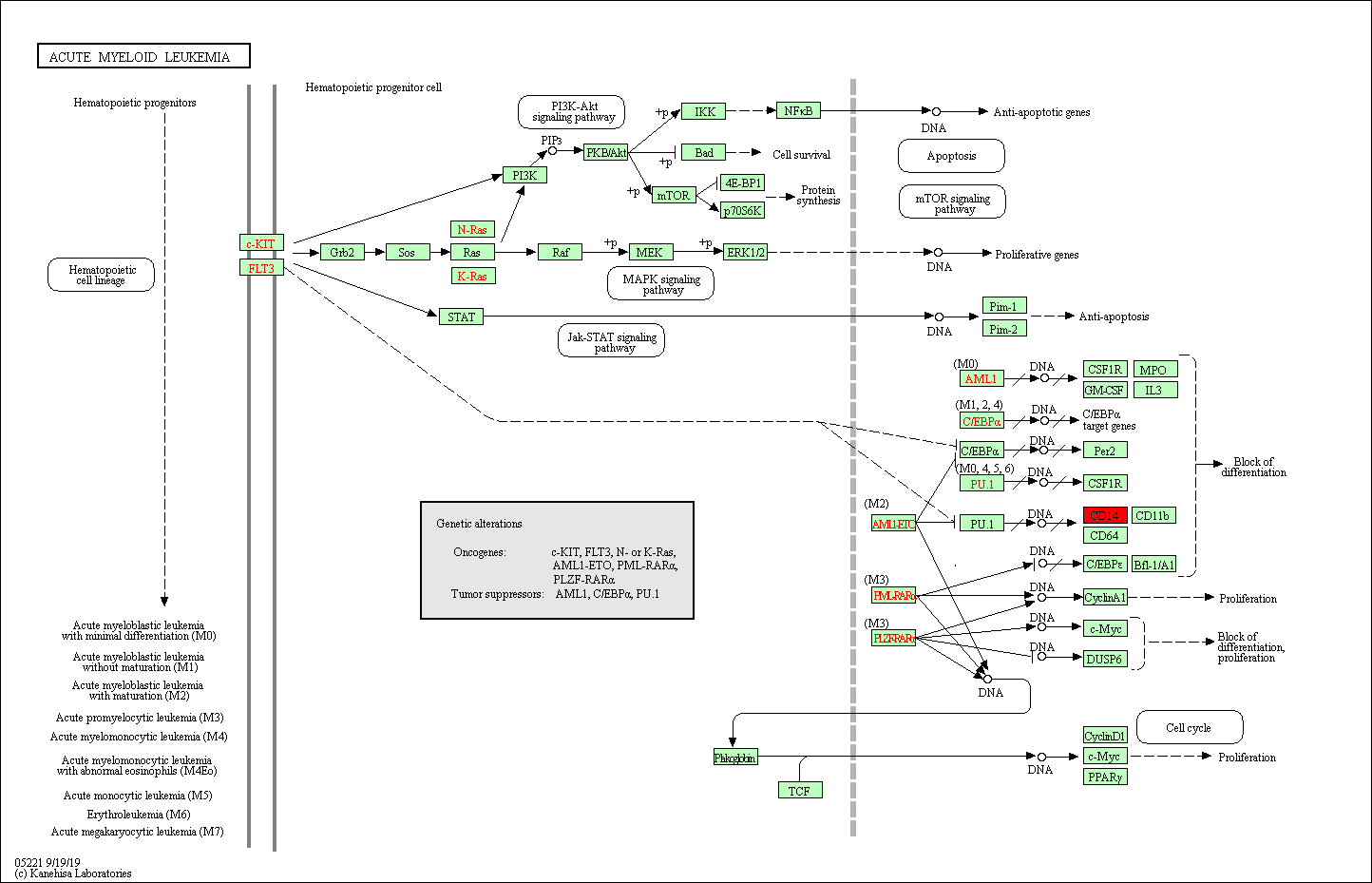

Supplement: Supplementary file 5 [file Data_Sheet_2.zip › Bioinformatics analysis related attachments/KEGG/Sig(QF_BQF)/png/bta05221.png]

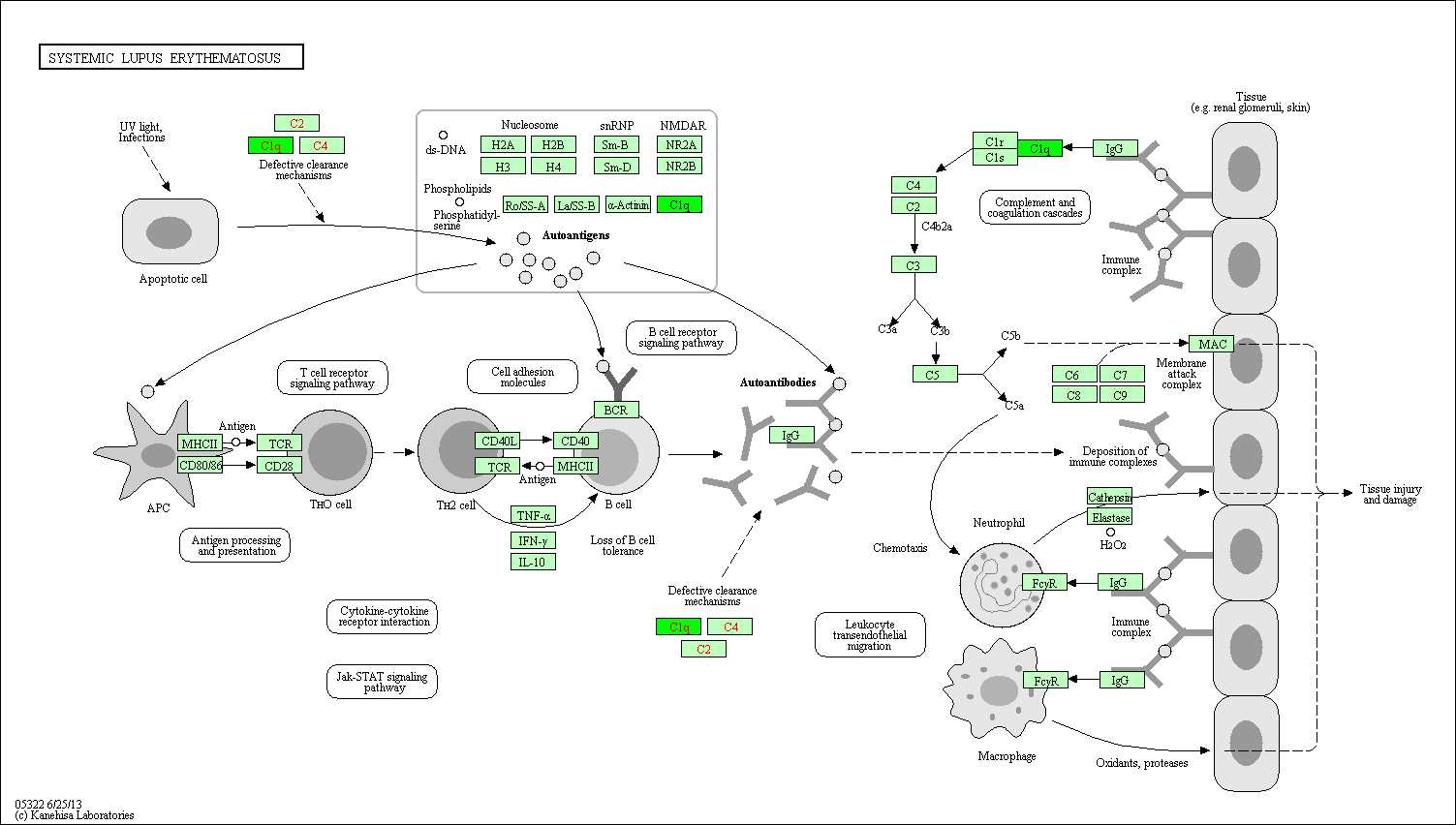

Supplement: Supplementary file 5 [file Data_Sheet_2.zip › Bioinformatics analysis related attachments/KEGG/Sig(QF_BQF)/png/bta05322.png]
